# Distinct dopaminergic spike-timing-dependent plasticity rules are suited to different functional roles

**DOI:** 10.1101/2024.06.24.600372

**Published:** 2024-06-27

**Authors:** Baram Sosis, Jonathan E. Rubin

**Affiliations:** 1 Department of Mathematics, University of Pittsburgh, PA, USA; 2 Center for Neuroscience and Center for the Neural Basis of Cognition, University of Pittsburgh, PA USA

## Abstract

Various mathematical models have been formulated to describe the changes in synaptic strengths resulting from spike-timing-dependent plasticity (STDP). A subset of these models include a third factor, dopamine, which interacts with the timing of pre- and postsynaptic spiking to contribute to plasticity at specific synapses, notably those from cortex to striatum at the input layer of the basal ganglia. Theoretical work to analyze these plasticity models has largely focused on abstract issues, such as the conditions under which they may promote synchronization and the weight distributions induced by inputs with simple correlation structures, rather than on scenarios associated with specific tasks, and has generally not considered dopamine-dependent forms of STDP. In this paper, we analyze, mathematically and with simulations, three forms of dopamine-modulated STDP in three scenarios that are relevant to corticostriatal synapses. Two of the models considered comprise previously proposed STDP rules with modifications to incorporate dopamine, while the third is a corticostriatal dopamine-dependent STDP rule adapted from a similar one already in the literature. We test the ability of each of the three models to maintain its weights in the face of noise and to complete simple reward prediction and action selection tasks, studying the learned weight distributions and corresponding task performance in each setting. Interestingly, we find that each of the three plasticity rules is well suited to a subset of the scenarios studied but falls short in others. These results show that different tasks may require different forms of synaptic plasticity, yielding the prediction that the precise form of the STDP mechanism may vary across regions of the striatum, and other brain areas impacted by dopamine, that are involved in distinct computational functions.

## Author summary

Learning from feedback is a crucial ability that allows humans and other animals to respond and adapt to their environments. One important locus for such learning is the basal ganglia, where dopamine-modulated corticostriatal plasticity shapes the dynamics of the cortico-basal ganglia-thalamic network in response to feedback signals to promote adaptive behavior. In this paper we ask, what learning rule is best suited to modeling this dopamine-modulated plasticity? To that end we investigate three learning rules that incorporate spike-timing-dependent plasticity as well as dopaminergic modulation. We study their performance in several settings meant to model the kinds of tasks and scenarios that striatal neurons are likely to be involved in. Each plasticity rule we examined performs well in some settings but fails in others. Different plasticity mechanisms may therefore be better suited to different functional roles and potentially to different regions of the brain.

## Introduction

Learning and memory are critical features of cognition in both humans and non-human animals, and a number of neural learning mechanisms have been described. One important mechanism is spike-timing-dependent plasticity (STDP) [1], a class of Hebbian plasticity rules in which the relative timing of pre- and postsynaptic spikes determines the changes in synaptic connection strength. Typically, a presynaptic spike before a postsynaptic spike – that is, a causal ordering of the spikes – leads to synaptic potentiation, whereas the reverse order leads to depression. In many cases, though, synaptic plasticity depends not just on the timing of pre- and postsynaptic spikes but also on some third factor, such as a neuromodulatory signal or other input [2, 3]. These additional factors may act as gating signals, and their strength and timing may impact both the magnitude and the direction of synaptic changes.

A prominent example of neuromodulatory impact on synaptic plasticity occurs at the cortical inputs to the basal ganglia. The neuromodulator dopamine is released by midbrain dopamine neurons when unexpected reward is received [4, 5] and plays a crucial role in modulating plasticity of corticostriatal synapses [6]. Experimental evidence and theoretical modeling suggest that dopamine serves as a reward prediction error signal [7, 8], enabling the brain to learn to favor behaviors that lead to reward and disfavoring behaviors that do not; such findings have been recently reviewed [9]. Theoretical analysis of action selection and modulation by cortico-basal ganglia-thalamic (CBGT) circuits posits a role for these dopaminergic reward prediction error signals both in updating value estimates associated with available choices and, through their impact on corticostriatal synaptic strengths, in altering the likelihood that a particular action will be selected in the future [10–13]. These distinct functions are likely performed by different neurons in different regions of the basal ganglia, however, which raises the possibility that distinct plasticity rules are involved. Unfortunately, despite some exciting experimental investigations of long-term plasticity properties in specific striatal regions and task settings [14–16], relatively little is know about the details of these plasticity mechanisms, especially in striatal regions thought to encode value.

**Fig 1.**
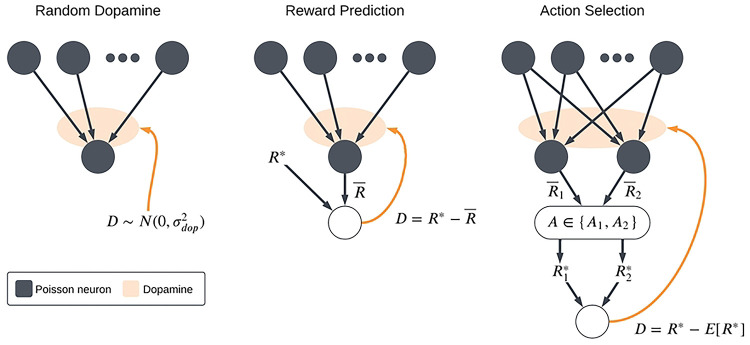
Schematic of the three main task settings. In the random dopamine setting, the neuron of interest receives stochastic cortical inputs not related to its primary function and dopamine signals resulting from activity elsewhere in the basal ganglia. In the reward prediction setting, the output firing rate is interpreted as a predicted reward and the dopamine signal is the reward prediction error. In the action selection setting, an action is chosen based on which of two competing channels has a higher output firing rate. A reward is then received based on the action taken and the dopamine again represents reward prediction error.

These considerations lead to the question of how well particular implementations of dopaminergic plasticity can perform the kinds of tasks or fulfill the kinds of roles that the corticostriatal system is believed to execute in the brain. If a plasticity model is capable of supporting biologically-relevant tasks, then that serves as some evidence in favor of the model; conversely, if it fails to do so, then we may want to modify it or look for other alternatives. To that end, we describe three models of dopaminergic plasticity, two derived by extending more general models of STDP learning to incorporate dopaminergic modulation and one designed specifically to model corticostriatal plasticity, as well as some variations on these models. We consider their performance in several different task settings relevant to the striatum, illustrated in Fig 1. As a baseline, we study synaptic weight evolution in a neuron receiving random, uncorrelated inputs and dopamine; this is meant to model a neuron uninvolved in whatever task the animal is performing. We also study simple models of reward prediction and action selection, two tasks in which the basal ganglia are believed to play major roles [11, 17–24]. Finally, we examine some more complex variants of these settings in which the reward contingencies or the task changes periodically. We find that although each model does well in some, no model is able to succeed in all the scenarios we consider. Thus, our results suggest that the brain may need to employ distinct, specialized plasticity mechanisms to learn different tasks.

## Models

### Plasticity Models

Here we introduce three models of dopaminergic spike-timing-dependent plasticity. The *additive* and *multiplicative models* are based on models of STDP (without dopamine) described in [25, 26] (for the additive model) and [27–29] (for the multiplicative model); we mainly follow the presentation in [30]. The *corticostriatal model* is based on a computational model of the corticostriatal connections described in [31], specifically connections onto striatal spiny projection neurons that express the D1 dopamine receptor, sometimes referred to as direct pathway SPNs.

We consider linear Poisson neurons: presynaptic spike trains are modeled as Poisson processes $\rho_{i}^{pre}(t)$ with constant rate $r_{i}={\left\langle \rho_{i}^{pre}(t)\right\rangle }_{t}$ (where i=1,2,…,N), and likewise spike trains of the single postsynaptic neuron are Poisson processes $\rho^{\text{post}}(t)$ with instantaneous firing rate functions R(t) given by a linear combination of the presynaptic spike trains:

(1) $$R\left(t\right)=\frac{1}{N}\sum_{i=1}^{N}w_{i}\left(t\right)\rho_{i}^{pre}\left(t-\epsilon \right)$$

where ϵ>0 is a small synaptic delay and $w_{i}$ are the synaptic efficacies, which we will also call weights, which we normalize to [0, 1]. (We will write the vectors of input firing rates and weights as $r,\;w\in R^{N}$.) This can be implemented by first generating input spike trains $\rho_{i}^{pre}(t)$, and then, whenever a presynaptic spike from input unit i occurs, say at time $t_{\text{pre}}$, adding a postsynaptic spike at time $t_{\text{post}}=t_{\text{pre}}+\epsilon$ to the postsynaptic spike train $\rho^{\text{post}}$ with probability $\frac{1}{N}w_{i}\left(t_{\text{pre}}\right)$. We assume that the input spike trains are uncorrelated.

Rather than modifying each synapse immediately with the occurrence every spike pair, as in a classical two-factor STDP rule, we instead assume that an eligibility trace [32, 33] for that synapse is incremented, which decays exponentially between spike pairs. Then the weight change is proportional to both the current value of the eligibility trace and the value of the dopamine signal, described below.

To implement this model we follow [13] and use a set of trace variables to track the influences of pre- and postsynaptic spikes and spike pairs. We define $A_{i}^{pre}(t)$ and $A^{\text{post}}(t)$ to track the pre- and postsynaptic spiking:

(2) $$\frac{dA_{i}^{pre}}{dt}\;=\rho_{i}^{pre}(t)-\frac{1}{\tau }A_{i}^{pre}(t)\;\frac{dA^{post}}{dt}\;=\rho^{post}(t)-\frac{1}{\tau }A^{post}(t)$$

with decay constant τ>0. We also define eligibility traces to track spike pairs. An important assumption in our analysis, made by [28, 30] and others, is that changes in weight from individual spike pairs can be summed independently. To realize this, we define two eligibility traces, $E_{i}^{+}(t)$ and $E_{i}^{-}(t)$, to track pre-before-post and post-before-pre spike pairs, respectively:

(3) $$\frac{dE_{i}^{+}}{dt}\;=\rho^{post}(t)A_{i}^{pre}(t)-\frac{1}{\tau_{eli}}E_{i}^{+}(t)\;\frac{dE_{i}^{-}}{dt}\;=\rho_{i}^{pre}(t)A^{post}(t)-\frac{1}{\tau_{eli}}E_{i}^{-}(t)$$

with decay constant $\tau_{\mathrm{eli}}>0$. We use two independent traces in part because experimental results have suggested that this independence is present in cortical synapses [34]. Moreover, using a single trace, as done previously [13], allows spike pairs to interact nonlinearly and partially cancel each other out, while using two traces ensures that different spike pairs do not interact, which is convenient for analysis. We discuss this issue further in Results, Single Eligibility Trace.

We assume that dopamine is released at fixed intervals of length $1/r^{dop}$ for constant $r_{dop}>0$; otherwise, it decays exponentially:

$$\frac{dD}{dt}=\sum_{k}D_{k}\delta \left(t-k/r^{dop}\right)-\frac{1}{\tau_{dop}}D$$

The value of the dopamine increment $D_{k}$ depends on the task setting; see Models, Task Settings. (We will treat this signal as the dopamine level *relative to some baseline*, rather than the raw dopamine concentration itself; so, in the absence of any signal, D equals zero, and we allow $D_{k}<0$.) Note that while the dopamine *concentration* may depend on the postsynaptic spike train, we assume for analytical convenience that the *timing* of dopamine delivery is independent of the spiking activity. Here we assume the dopamine is simply delivered periodically for simplicity; the precise form of the dopamine process is irrelevant as long as it has mean rate $r^{\text{dop}}$, is independent of the spike trains, and yields dopamine signals that are far enough apart that their interactions can be neglected.

Finally, the weights in Eq (1) are updated using the values of the dopamine signal and the eligibility traces in a way that depends on the choice of plasticity model. The additive and multiplicative models use the following rule:

(4) $$\frac{dw_{i}}{dt}=\lambda D(t)\left(f_{+}\left(w_{i}(t)\right)E_{i}^{+}(t)-f_{-}\left(w_{i}(t)\right)E_{i}^{-}(t)\right)$$

where $f_{+}(w)=(1-w{)}^{\mu }$ and $f_{-}(w)=\alpha w^{\mu }$ apply different scaling factors to weight updates from positive and negative eligibility. λ>0 is the learning rate, α tunes how strongly negative eligibility is weighted relative to positive eligibility (typically α≥1), and μ∈[0,1] selects from a family of different possible update functions. We will only consider the cases μ=0, known as the additive model, and μ=1, known as the multiplicative model. (See [30] for an exploration of the effects of varying μ in a simpler two-factor STDP setting.)

The corticostriatal model is broadly similar, but modifies the functional form of the weight update depending on the sign of the weight change. Rather than using the $f_{+}/f_{-}$ functions defined above, we use f(w)=1-w when the sign of the overall weight change (including the sign of the dopamine signal and the sign of the eligibility) is positive, and f(w)=αw when it is negative. This convention is described by the formula:

(5) $$\frac{dw_{i}}{dt}=\left\{\begin{array}{ll} \lambda D(t)\left(\left(1-w_{i}(t)\right)E_{i}^{+}(t)-\alpha w_{i}(t)E_{i}^{-}(t)\right) & \text{if}\;D(t)\geq 0\\ \lambda D(t)\left(\alpha w_{i}(t)E_{i}^{+}(t)-\left(1-w_{i}(t)\right)E_{i}^{-}(t)\right) & \text{if}\;D(t)<0 \end{array}\right.$$

In all three models, synapses become stronger with above-baseline dopamine signals (and weaker with below-baseline dopamine signals) when the postsynaptic neuron has recently participated in a pre-before-post spike pairing, and weights change in the opposite direction following post-before-pre spike pairs. This is to match the observed behavior of cortical synapses onto striatal spiny projection neurons expressing specifically D1 dopamine receptors [10, 12, 13, 31, 35, 36]. Neurons expressing D2 receptors show the opposite behavior, but we do not consider those here.

Table 1 shows how the scaling factors used by each of the three models depends on the signs of the dopamine and eligibility. The additive and multiplicative models only depend on the sign of the eligibility, while the corticostriatal model uses the sign of the product of dopamine and eligibility to determine which scaling factor to use. This means that when using the corticostriatal model, the scaling factor corresponds to the direction in which the weights will change: 1-w for increasing weights and αw for decreasing weights. In contrast, the scaling factors used by the additive and multiplicative models do not correspond to the direction of weight change.

While we primarily focus on the additive, multiplicative, and corticostriatal models in this paper, we will also explore some variations on these models. In Results, Single Eligibility Trace we describe versions of these three models using a single eligibility trace, rather than the two traces we use elsewhere. In Results, Symmetric Model we also explore a new model, which we term the *symmetric model*, meant to alleviate some of the issues we find with the other three plasticity rules.

For all of the models, weights are kept bounded between 0 and 1; for the additive and multiplicative models, this necessitates clipping weights that would go beyond these limits based on Eq (4) alone.

### Task Settings

The plasticity models described above are agnostic as to how exactly the dopamine signal is computed. We consider three different task settings, corresponding to three different scenarios or functional roles that may arise with striatal neurons (see Fig 1). The first is what we will refer to as the *random dopamine setting*: dopamine is sampled from a normal distribution centered at zero, $D\sim 𝒩(0,\sigma_{\text{dop}}^{2})$, independently of the spiking activity. This models a neuron that is uninvolved in whatever task the animal is performing; it may receive some spurious inputs and dopamine due to activity elsewhere, but its inputs and output are statistically independent of the dopamine release. We would like a plasticity model that yields zero net weight drift under random dopamine, so that previous learning is not erased. While the stochastic inputs and dopamine may perturb the weights somewhat, ideally it should not cause them to move consistently in one direction or another.

**Fig 2.**
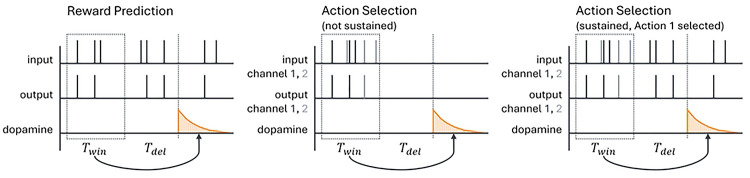
Schematic of the sequence of events in the reward prediction and action selection models. Output spikes are counted in a window of length $T_{\text{win}}$ to estimate the average output firing rate; then, after a delay of length $T_{\text{del}}$ (which may be zero), dopamine is released. For the action selection model there are two channels, colored black and gray, corresponding to the two actions being considered. We examine two versions of the action selection model: one in which the cortical input is suppressed outside of the spike count window, and one in which activity is maintained in the selected channel (here channel 1). These two variants are most similar when $T_{del}=0$ (although they still differ due to spikes occurring after dopamine is released), and we often will consider that case, but we will also compare it to results with $T_{del}>0$ as shown in the figure.

The second scenario that we will consider is the *reward prediction setting*. In this model, we assume that the dopamine signal takes the form of an error signal between the firing rate of the postsynaptic neuron and some target firing rate $R^{*}$. We mainly view this as a reward prediction error [7, 8], as evidence suggests that the ventral striatum plays a major role in processing value estimates [37–39]. But this framework can also be applied more generally, as long as we assume that there is some optimal firing rate $R^{*}$ for whatever task the animal may be performing and that the error signal is proportional to the difference between $R^{*}$ and the actual firing rate. We compute D as follows:

(6) $$D=R^{*}-\overset{‾}{R}$$

where

(7) $$\overset{‾}{R}=\frac{1}{T_{win}}\int_{t_{dop}-T_{win}-T_{del}}^{t_{dop}-T_{del}}\rho^{post}(t)dt$$

is an estimate of the current firing rate. Here $T_{\text{win}}$ is the length of the time window over which the spike train is averaged (e.g., to produce a value estimate) and $T_{\text{del}}$ is a delay term between when the output firing rate is measured and when the dopamine is actually released (Fig 2A). This delay could be due to biological constraints, such as the speed of neural signal propagation or motor response, or to experimentally imposed delays; it has a significant impact on the dynamics, as will be discussed below. For this model, we would like a plasticity mechanism that can learn to match the target firing rate $R^{*}$ on average, so that $R^{*}=E[\overset{‾}{R}]$.

The final model that we will consider is the *action selection setting*, as the basal ganglia including dorsal regions of striatum are hypothesized to play a critical role in action selection [40, 41]. We implement this as a competition between two action channels [41–44]. Two neurons with weight vectors $w^{1}$ and $w^{2}$ (with entries $w_{i}^{j}$ for i∈{1,2,…,N} and j∈{1,2}) receive independent input spike trains generated from identical rate vectors r corresponding to shared presynaptic input sources. We compute estimates of their current firing rates ${\overset{‾}{R}}_{1},$ ${\overset{‾}{R}}_{2}$ as in Eq (7), although unlike in the reward prediction setting we will usually set $T_{del}=0$ (see the discussion in Results, Action Selection Setting). The animal then randomly chooses one of two actions, $A_{1}$ or $A_{2}$, using the output firing rates to determine the selection probabilities:

$$P_{j}=P\left(A=A_{j}\right)=\frac{e^{\beta {\overset{‾}{R}}_{j}}}{e^{\beta {\overset{‾}{R}}_{1}}\;+\;e^{\beta {\overset{‾}{R}}_{2}}}$$

for j∈{1,2}, where β is an inverse temperature parameter. (For simplicity, we take β to be an arbitrary large number in simulations, so that actions are chosen deterministically based on which channel has more spikes, with ties broken randomly.) The animal receives a reward $R^{*}$ depending on which action is taken: $R_{1}^{*}$ if $A_{1}$ is chosen, $R_{2}^{*}$ if $A_{2}$ is chosen. Finally, we compute the dopamine signal as the reward prediction error:

(8) $$D=R^{*}-E\left[R^{*}\right]=R^{*}-\left(R_{1}^{*}E\left[P_{1}\right]+R_{2}^{*}E\left[P_{2}\right]\right),$$

which is used to update the synaptic weights and hence $\rho^{\text{post}}(t)$ and $\overset{‾}{R}$, thus impacting future action selection. We would like a plasticity model that can learn to more frequently take the action that gives the higher reward.

In Eq (8), $P_{1}$ and $P_{2}$ are now treated as random variables; we take the average value of $P_{1}$ and $P_{2}$ over instantiations of the spike trains with the given rates. That is, we sum over the possible postsynaptic spike counts in each channel:

$$E\left[P_{1}\right]=\sum_{i=0}^{\infty }\sum_{j=0}^{\infty }\frac{e^{i\beta /T_{\text{win}\;}}}{e^{i\beta /T_{\text{win}\;}}+\;e^{j\beta /T_{\text{win}\;}}}\frac{n_{1}^{i}e^{-n_{1}}}{i!}\frac{n_{2}^{j}e^{-n_{2}}}{j!}$$

where

$$n_{k}=T_{win}\frac{\left\langle w^{k},r\right\rangle }{N},\;k\in \{1,\;2\}$$

is the expected number of postsynaptic spikes in a window of length $T_{\text{win}};$ $E\left[P_{2}\right]$ is similar.

The definition assumes that the agent’s state-value function [33] is accurate. In other words, the animal has learned the reward it receives on average when performing this task with its current policy (as defined by the weights $w^{1}$ and $w^{2}$). The idea that value estimates are available to neurons that drive action selection is commonly used in models and has ample experimental support (e.g, [45, 46]). In practice these value estimates have to be learned, and as the animal’s policy changes, the value estimates will have to evolve along with it. We assume that the value estimates remain accurate (i.e., are learned instantaneously relative to the timescale of decision policy changes) as a simplification to allow us to focus on the action selection task without the added complication of a separate value learning circuit.

In the action selection setting, we silence all input to the striatal neuron between the end of one spike count window and the beginning of the next (i.e. for the duration of the delay if $T_{\text{del}}\neq 0$ as well as the period after dopamine is released). This step is designed to represent typical experimental settings in which the input stimulus does not persist after an action is taken in response to the stimulus. For instance, in a task in which a rodent must choose which branch of a maze to follow to receive a reward, the stimulus – the sight of the junction – necessarily cannot persist after the animal has made a choice and gone down one of the branches. However, we also consider a modification in which the cortex maintains some level of activity in the channel corresponding to the selected action [47, 48] to help correctly assign credit for rewards to actions when they are separated by significant delays. We discuss this modification in more detail in Results, Action Selection Setting. Fig 2 shows an illustration of the two versions of the action selection model as well as a comparison to the reward prediction model.

We also consider two variations on these basic scenarios. The first is *reward contingency switching* [13, 49], a variation of the action selection setting in which the mapping between actions and rewards is swapped periodically. The plasticity model should be able to update the learned weights based on the new reward schedule and switch which action it takes. The second is *task switching*, in which not only the rewards but also the input firing rate vector r switches between two (or more) possible values. Task switching can be applied to both the reward prediction setting and the action selection setting. In contrast to contingency switching, in which the neuron must switch which action it selects, in the task switching setting the neuron would ideally learn to perform *both* tasks using the same set of weights. (Of course, this is only possible in non-degenerate cases if the input dimension N is at least equal to the number of tasks to be learned.) This variant models the fact that a neuron will generally not be restricted to performing a single task, but rather may be active in a variety of different contexts.

One important simplification that we make in all settings is that the *timing* of dopamine release is independent of the spiking activity, and is simply treated as coming at some random time with mean rate $r^{dop}$. We also assume that dopamine releases are far enough apart that the dopamine level decays approximately to zero between them; in simulations, we implement this by simply using a fixed time interval between dopamine releases. These conventions contrast with models like the one described in [13], which count the number of output spikes in a moving window and take an action (and subsequently release dopamine) as soon as the number crosses some threshold, and with models in which the CBGT circuit performs a process of evidence accumulation up to some threshold to make a decision [44, 50–53]. We opted to use a simpler mechanism here for analytical tractability. Although this may at first seem like a major simplification, in reality, if the neurons’ inputs in our tasks are statistically similar throughout the decision or reward estimation process as spikes are accumulated, then the output spiking characteristics preceding dopamine release on average are not related to the actual timing of dopamine release, only to its magnitude.

### Simulations

We use the parameters listed in Table 2 as the defaults in our simulations for each of the three main task settings; any other parameters or changes to the defaults are listed in figure captions. “Steps” refers to the number of dopamine signals in an experiment; the number of steps used as well as λ were chosen to balance noise level with computation time and to illustrate phenomena of interest. $w_{\text{init}}=0.5$ was chosen arbitrarily; in some plots we instead use 0*.*33 to illustrate time dynamics of weights because 0*.*5 would be too close to values that weights converge to. We chose input firing rates r to roughly match the frequency of cortical input to the striatum. As the random dopamine setting is meant to model neurons receiving spurious inputs, we use a lower input firing rate there. $R^{*},$ $R_{1}^{*}$, and $R_{2}^{*}$ are arbitrary and were chosen for illustrative purposes. We use α=1 as the default scaling parameter in our weight update equations (Eqs (4) and (5)) for simplicity. For the choice of τ=0.02s in Eq (2), see [30, 54]; note that [55] give values of τ=0.0168s for long-term potentiation (LTP) and τ=0.0337s for long-term depression (LTD), but as we do not distinguish between LTP and LTD in our model, we use the intermediate value of τ=0.02s used in other sources. The half-life of dopamine has been estimated as 0*.*72 s [56]; translating the half-life into an exponential time constant we get $\tau_{dop}\approx 1\;s$. The choice of eligibility time constant $\tau_{\text{eli}}=1\;s$ reflects experimentally derived estimates [57, 58] (but see [59], which finds a somewhat larger value). In the reward prediction setting, the delay time for dopamine release, $T_{\text{del}}$, was chosen to be long enough that any spikes occurring before the delay have minimal impact on the weight changes. In the action selection setting we generally use $T_{\text{del}}=0.r^{\text{dop}}$ was likewise chosen to be small enough that the effects of any interactions between adjoining dopamine signals would be negligible. The reward prediction and action settings use a longer period between dopamine signals than that used in the random dopamine setting to allow for the window $T_{\text{win}}$. The constant $\beta =10^{5}$ in the probability calculations is an arbitrary choice; other sufficiently large values would give similar results.

All figures use a sample size of 1000 for numerical results; error bars and bands show standard deviations. In some phase portraits we include fixed points; these were found analytically when possible, otherwise they were computed using the scipy.optimize library [60]. Note that some of the fixed points found on the boundaries are not “true” fixed points in the sense that they are not zeros of the dynamical equations. Rather, they are the result of the weights being clipped at 0 and 1.

## Results

### Random Dopamine Setting

When the dopamine signal is independent of spiking activity and has mean zero, the additive and multiplicative models in theory should exhibit zero net weight drift. This result arises because the dopamine is independent of the other terms in the weight update equation (4), so when taking the average weight drift we can factor out the average dopamine level, which is zero. This is not the case, however, for the corticostriatal model; here, the form of the weight update equation (5) depends on the sign of the dopamine signal, so the terms are not independent. It can be shown (see Section 4 in S1 Appendix) that on average the weights for the corticostriatal model converge to 1/(α+1).

**Fig 3.**
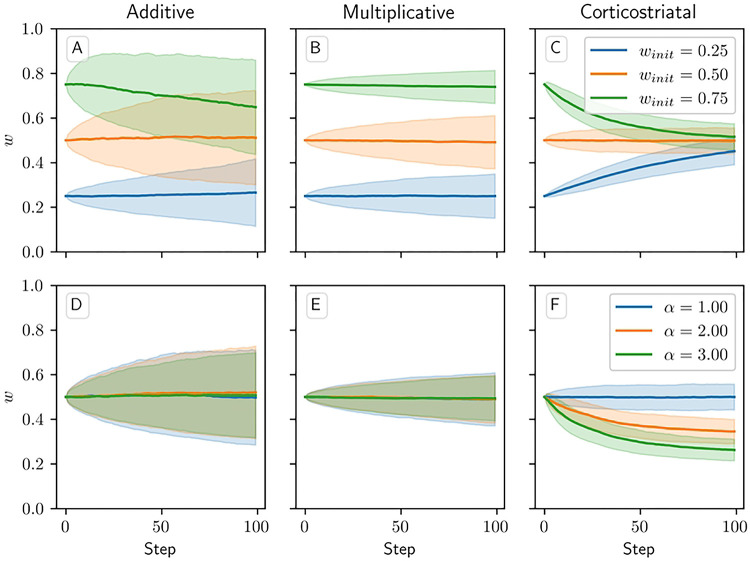
Weight evolution over time in the random dopamine setting. Columns show the additive (A, D), multiplicative (B, E), and corticostriatal (C, F) models. (A-C) the initial weight $w_{\text{init}}$ is varied while α=1 is fixed. (D-F) α is varied while $w_{\text{init}}=0.5$ is fixed.

These outcomes are illustrated in Fig 3. In practice, the weights for the additive and multiplicative models do show some fluctuations about their means, which grow over time, as well as some boundary effects where clipping the weights to 0 and 1 pushes the mean weight values away from the boundaries. This is most visible for the additive model. In this case, the weight drift is proportional to w, so the upper curve will experience larger fluctuations than the lower curve; moreover, since weight increases are being truncated, there is a bias that causes a net downward drift. However, motion away from the initial conditions for both models is generally fairly slow. In contrast, the mean weight values for the corticostriatal model quickly converge to 1/(α+1). Thus, under the corticostriatal model without any supplementary weight maintenance mechanism, any noise will tend to erase previously learned weights.

### Reward Prediction Setting

Under suitable assumptions it is possible to derive a formula for the average weight drift over time for the additive and multiplicative models under the reward prediction framework:

(9) $${\dot{w}}_{i}=\left(R^{*}-\frac{1}{N}\langle w,r\rangle \right)r^{dop}\tau_{dop}\tau_{eli}\frac{\lambda }{N}\left(\tau \Delta f\left(w_{i}\right)r_{i}\langle w,r\rangle +f_{+}\left(w_{i}\right)w_{i}r_{i}\right)$$

where $\Delta f=f_{+}-f_{-}$. (See Section 1.1 in S1 Appendix for the derivation. An important assumption here is that the delay $T_{\text{del}}$ is large relative to $\tau_{\text{eli}}$; this assumption will be discussed below.) The terms in this expression have a simple interpretation: $\tau \Delta f\left(w_{i}\right)r_{i}\langle w,r\rangle$ corresponds to independent pairs of pre- and postsynaptic spikes (both pre-before-post and post-before-pre), $f_{+}\left(w_{i}\right)w_{i}r_{i}$ corresponds to a pre-post spike pair in which the presynaptic spike directly causes the postsynaptic neuron to fire, and $R^{*}-\frac{1}{N}\langle w,r\rangle$ is the average dopamine level, which is the difference between the target firing rate and the mean output firing rate.

The average weight drift equation (9) is fairly easy to analyze. Its most important feature is what we will call the *solution plane*: the hyperplane of weight values such that $\frac{1}{N}\langle w,\;r\rangle =R^{*}$. These are weights such that the output firing rate equals the target firing rate $R^{*}$ and hence they are solutions to the task the neuron has to learn. It is clear from Eq (9) that any point on this plane is a fixed point, which corresponds to the average dopamine signal being zero. However, the solution plane is not necessarily stable. We give a sufficient condition for the existence of a stable solution (that is, a stable fixed point on the solution plane) in the following theorem.

#### Theorem 1.

*Pick* $r\in R^{N}$ *and* $R^{*}\leq \frac{1}{N}∥r{∥}_{1},$ *and let* $w^{\prime }=NR^{*}/∥r{∥}_{1}$*. If*

(10) $$f_{-}\left(w^{\prime }\right)<\left(1+\frac{1}{\tau ∥r{∥}_{1}}\right)f_{+}\left(w^{\prime }\right),$$

*then there exists a stable point on the solution plane, given by* $w=\left(w^{\prime },\ldots ,w^{\prime }\right)$.

*Proof.* See Section 2.2 in S1 Appendix.

For the additive model, condition (10) can be rewritten as

$$\tau (\alpha -1)<\frac{1}{∥r{∥}_{1}}$$

whereas for the multiplicative model, it can be written as

$$R^{*}<\frac{w_{0}}{N}∥r{∥}_{1}$$

where

$$w_{0}=\frac{\tau ∥r{∥}_{1}\;+\;1}{\tau \left(1\;+\;\alpha \right)∥r{∥}_{1}\;+\;1}.$$

See Section 2 in S1 Appendix for derivations and more details on the stability of the solution plane.

The additive model in general has no other equilibria besides the solution plane (and points on the boundary). However, when $\tau (\alpha -1)=1/∥r{∥}_{1}$, points on the line $w_{i}=w_{j}$ for all i≠j are also equilibria. Points on the line are stable when $R^{*}-\frac{1}{N}\langle w,r\rangle <0$; that is, the line is stable on one side of the solution plane. This statement can be proven using a similar approach to that used in Section 3.1 in S1 Appendix.

The multiplicative model has an extra fixed point at $w=\left(w_{0},\ldots ,w_{0}\right)$. We can characterize its stability as follows:

#### Theorem 2.

*For the multiplicative model, if*

$$R^{*}<\frac{w_{0}}{N}∥r{∥}_{1},$$

*then the fixed point* $w=\left(w_{0},\ldots ,w_{0}\right)$ *is unstable; if*

$$R^{*}>\frac{w_{0}}{N}∥r{∥}_{1},$$

*then the point is stable*.

*Proof.* See Section 3.2 in S1 Appendix.

The corticostriatal model, however, is more difficult to analyze. Because the form of the plasticity rule depends on the sign of the dopamine signal, in general it is not possible to factor out the average dopamine level $R^{*}-\frac{1}{N}\langle w,r\rangle$ like we can for the additive and multiplicative models. We analyze this model further in Section 1.2 in S1 Appendix; in general, points on the solution plane will not be fixed points under this model.

**Fig 4.**
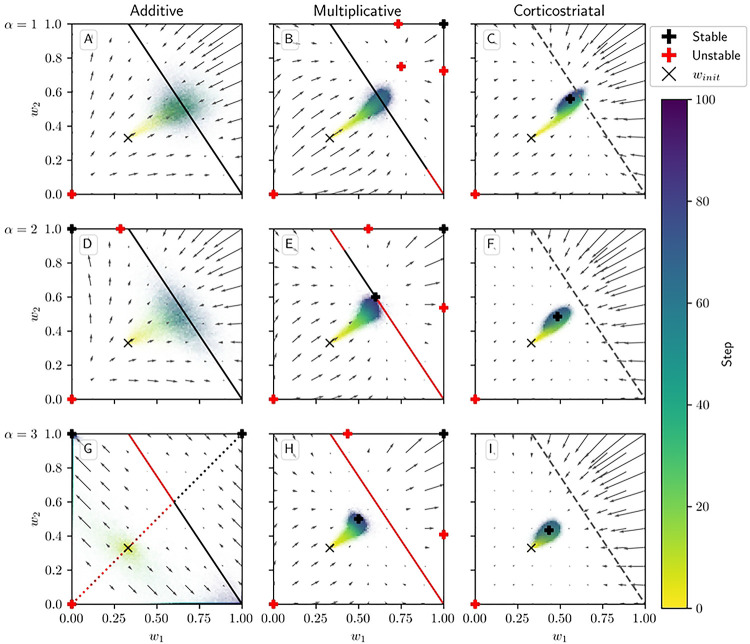
Distribution of weights over time in the reward prediction setting as α is varied. The color code indicated in the color bar shows the simulation step. Columns show the additive (A, D, G), multiplicative (B, E, H), and corticostriatal (C, F, I) models. α is varied across rows: (A-C) α=1; (D-F) α=2; (G-I) α=3. Each panel includes arrows showing the vector field of the average model as well as the solution plane, which is the negatively sloped line. For the corticostriatal model, the solution plane is dashed because it does not govern the dynamics for this model. Red coloring of the solution plane and points off of the plane indicates unstable fixed points; black indicates stable. We use $w_{\text{init}}=0.33$ here, marked by the “×” in each plot. (G) has an extra line of equilibria where $w_{1}=w_{2}$ (dotted). Note that in (G) most of the sample paths end up being driven to the upper left and lower right corners.

Fig 4 shows phase portraits for the averaged models for 3 different values of α. To generate each plot, we ran a set of simulations of the fully stochastic implementation (see Models, Simulations) of the appropriate model with N=2 weights and initial conditions $\left(w_{1},w_{2}\right)=0.33$ as marked by the × symbol. In each simulation, from this starting point, $w_{1}$ and $w_{2}$ evolved over 100 time steps, and the position of $\left(w_{1},w_{2}\right)$ at certain time steps was plotted as a point of the time-dependent color indicated by the color bar; this process resulted in a cloud of points over many simulations, representing the distribution of weights. Each plot also includes the solution plane (here, a line), any relevant fixed points, and vector field arrows for the averaged model. The orientations of these arrows indicate the directions that trajectories would move over time under the flow of the averaged model, while their lengths represent the magnitudes of the weights’ rates of change.

We see that as α increases, a larger fraction of the solution plane becomes unstable for the additive and multiplicative models. Fig 4G includes the additive model’s extra line of fixed points that exists for certain parameter values, as mentioned above. For the multiplicative model, the isolated extra fixed point crosses the solution plane and exchanges stability with it (Fig 4B, E, H). The solution plane does not consist of equilibria for the corticostriatal model; as can be seen, it does not play a role in shaping the model’s dynamics like it does for the additive and multiplicative models. In general, the averaged models capture the dynamics well, as can be seen from the dispersal patterns of trajectories in relation to the averaged model vector field and their convergence to stable fixed points, although depending on the model and the choice of parameters there can be substantial variability across these trajectories.

These results show that while the additive and multiplicative models can perform reward prediction tasks under suitable choices of the parameters, the corticostriatal model cannot. In the latter case, weights in general do not converge to the solution plane, so the postsynaptic neuron’s output firing rate will not match $R^{*}$. In contrast, with the appropriate parameters, most or all of the solution plane may be stable for the additive and multiplicative models. The additive model in fact performs best here, because it does not have the extra fixed point of the multiplicative model; even when the solution plane is stable, the unstable extra fixed point can drive trajectories from some initial conditions away from the solution plane towards the boundaries, as occurs for initial conditions with large enough $w_{1},$ $w_{2}$ in Fig 4B. Meanwhile, most trajectories under the additive model appear to converge to the solution plane, although for large enough α the convergent proportion drops as more of the plane becomes unstable. Our mathematical results serve to characterize the ranges of parameter values where stable points on the solution plane exist. Specifically, they highlight the important role that α and τ play in the dynamics: increasing either parameter reduces the range of r and $R^{*}$ values that support stable solutions.

Next, we consider task switching in which the rewards and input firing rates switch between two values. In this scenario, the average model dynamics can be quite complex, as there are two solution planes, one per set of rewards and input rates, each of which may have stable and unstable regions. Moreover, depending on the length of time between task switches, the weights may either bounce between the fixed points for the different tasks (if they do not coincide) or approximately follow the average of the weight drift equations of the tasks. If the solution planes intersect, then we would like the weights to converge to their intersection, so that the neuron can accomplish both tasks. If they do not intersect, then ideally the weights should converge to some point close to both of them, which would constitute an approximate solution to both tasks.

**Fig 5.**
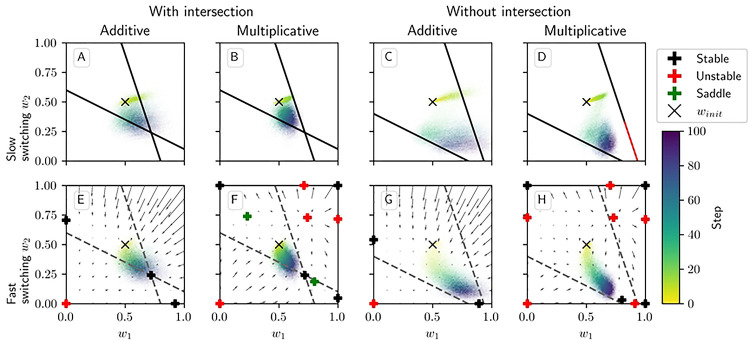
Distribution of weights over time in the reward prediction setting with task switching. First and third columns (A, C, E, G) show the additive model; second and fourth columns (B, D, F, H) show the multiplicative model. For (A, B, E, F) the task switches between $r=(15,\;5)\;s^{-1},$ $R^{*}=6$ and $r=(10,\;20)s^{-1},$ $R^{*}=6$, which yields a solution plane intersection at w=(0.72,0.24). For (C, D, G, H) the task switches between $r=(15,\;5)s^{-1},$ $R^{*}=7$ and $r=(10,\;20)s^{-1},$ $R^{*}=4$, which does not give a solution plane intersection in [0, 1]^2^. In the upper row (A-D) switching is slow (every 20 steps out of 100), while in the lower row (E-H) switching is fast (every step). For the slow switching plots we include the solution planes to demonstrate their relation to where the trajectories converge, but because switching between the two forms of dynamics is infrequent, we cannot display a meaningful vector field illustration. For the fast switching plots we include the vector field for the dynamics computed by averaging the dynamical equations for the two tasks; as switching is fast, averaging approximately captures the behavior of the system. We also plot the solution planes here for illustrative purposes, but they are dashed because they do not control the trajectories. Fixed points are estimated numerically: red indicates unstable fixed points; black indicates stable; green indicates saddle points. The “×” indicates the initial point, in this case $\left(w_{1},w_{2}\right)=(0.5,\;0.5)$.

Fig 5 shows the densities of trajectories of the additive and multiplicative models performing task switching in two example settings: one in which the solution planes intersect and one in which they do not. The upper row uses a long interval between switches, while the lower row switches after each step. (We do not include the corticostriatal model since, as discussed above, it cannot accomplish reward prediction tasks.) As can be seen, if the solution planes intersect, then (for suitable initial conditions) much of the density ends up concentrated at their intersection. If the planes do not intersect then the weights are generally driven to regions close to both planes, although results are less ideal in the multiplicative model due to complications such as an unstable solution plane segment (Fig 5D), an off-plane stable fixed point (Fig 5H), and regions of initial conditions that are impacted by an unstable fixed point (Fig 5H, upper right corner). Overall, while the precise details depend strongly on the choice of inputs and other parameters, the additive and multiplicative models do generally seem able to perform well at reward prediction in a task switching settings.

**Fig 6.**
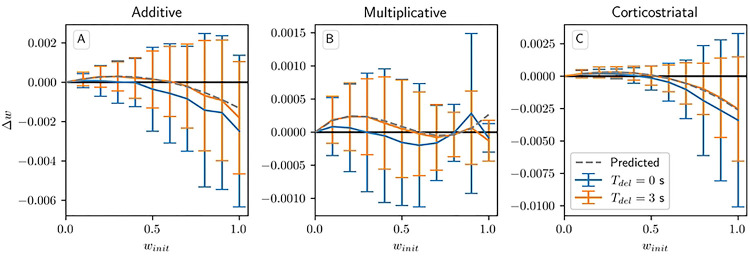
Weight drift after a single dopamine release in the reward prediction setting. Plots show results for the additive (A), multiplicative (B), and corticostriatal (C) models, with $T_{del}=0\;s$ and $T_{del}=3\;s$, as well as the predicted weight drift based on the averaged models, as $w_{\text{init}}$ is varied for N=1. Here $r=10{\;s}^{-1}$ and $R^{*}=6$; we also use $\tau_{\text{dop}}=0.1\;s,$ $\tau_{\text{eli}}=0.1\;s$, and $T_{\text{win}}=0.1\;s$ to highlight the deviation of the $T_{\text{del}}=0$ curve from predicted behavior. Note that when $w_{\text{init}}=1$ there are some deviations from predictions even for $T_{del}=3\;s$ due to boundary effects not taken into account by the averaged models.

All of these results, however, depend on a key assumption: that the delay $T_{\text{del}}$ is long relative to $\tau_{\text{eli}}$. By imposing a large gap between when the firing rate is measured, using Eq (7), and when dopamine is actually released, the delay ensures that the dopamine signal is statistically independent of the other terms in the weight update equation. This is what allows us to factor out the dopamine term $R^{*}-\frac{1}{N}\langle w,r\rangle$ in the average drift formula, Eq (9). Without this term, points on the solution plane $R^{*}=\frac{1}{N}\langle w,r\rangle$ are no longer necessarily equilibria for any of the three plasticity models. This can be seen in Fig 6, in which we plot the change in weight after a single dopamine release as a function of $w_{\text{init}}$ in an N=1 setting. When $T_{\text{del}}=3\;s$ the simulations obey the predictions of the averaged models, and in the additive and multiplicative cases they intersect the x-axis at w=0.6, the point at which $R^{*}=wr$ for these parameters. (While the plots may appear fairly noisy, keep in mind that they only display the change in weight after a single dopamine signal. Over the course of many trials this noise will to a large extent average out.) However, when $T_{\text{del}}=0\;s$ the simulations do not match the predictions, in most cases predicting weight changes below the $T_{\text{del}}=3\;s$ curves. This undershoot may occur because there is a source of negative correlation between the dopamine value and the eligibility at the time dopamine is released. Specifically, the dopamine value D from Eq (6) is negatively correlated with the number of postsynaptic spikes in the spike count window, while the eligibility will in most cases (depending on the plasticity model and the parameters) be positively correlated with the number of recent spikes. If there is no delay, then this will include the spikes in the spike count window used to compute the dopamine value. We picked the parameters for this plot to clearly illustrate this divergence from the averaged models; in general, the impact of this extra correlation will depend on the particular parameters chosen.

### Action Selection Setting

**Fig 7.**
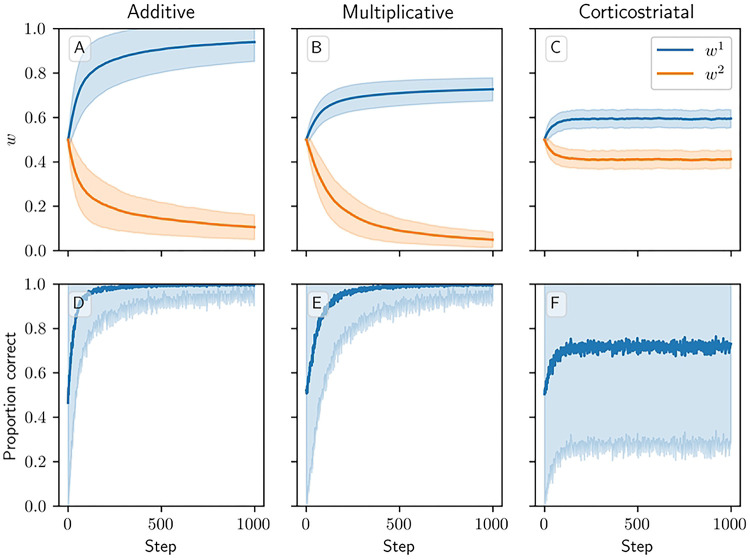
Model performance in the action selection setting. Plots show weights (A-C) and probability of taking the correct action (D-F) versus time for the additive (A, D), multiplicative (B, E), and corticostriatal (C, F) models. Shaded envelopes show standard deviations while solid lines show means over 1000 trials.

We next consider a task of selecting between two actions, in which action 1 gives a higher reward than action 2. In this setting, all three models successfully learn to take action 1 more often than action 2, but major differences arise among the values to which the weights converge across the three models (Fig 7). The additive model drives $w^{1}$ towards one and $w^{2}$ towards zero. The multiplicative model likewise drives $w^{2}$ towards zero, but $w^{1}$ only reaches around 0*.*73 ± 0*.*05 after 1000 steps. Meanwhile under the corticostriatal model, $w^{1}$ and $w^{2}$ converge to limits of around 0*.*56 ± 0*.*04 and 0*.*41 ± 0*.*04, respectively. (These values depend on the particular parameters chosen.) All three models can therefore accomplish this task, although the additive and multiplicative models choose the correct action more consistently than the corticostriatal model does (Fig 7).

The delay plays an important role in this model, too. S1 Fig shows the weight distributions after 1000 steps for the three models as a function of $T_{del}$; with too long of a delay, the models are unable to learn (i.e., the difference between $w_{1}$ and $w_{2}$ becomes too small) because the dopamine signal becomes uncorrelated with eligibility at the time dopamine is released. This is an instance of the *credit assignment problem* [32]. [48] propose that the brain solves this problem via sustained cortical activity in the selected action channel and reduced activity in the unselected channel, building off of experimental results showing this pattern of activity [47]. The corresponding sustained corticostriatal input ensures that while the spikes that directly caused an action to be selected do not themselves contribute to the weight changes, there will still be a correlation between the dopamine signal and the spiking activity at the time dopamine is released due to the differences in firing rates (see Fig 2). As can be seen in S1 Fig, with sustained activity in the selected channel the models are able to successfully produce large differences between $w_{1}$ and $w_{2}$, and hence learn the task, even when $T_{\text{del}}$ is large.

**Fig 8.**
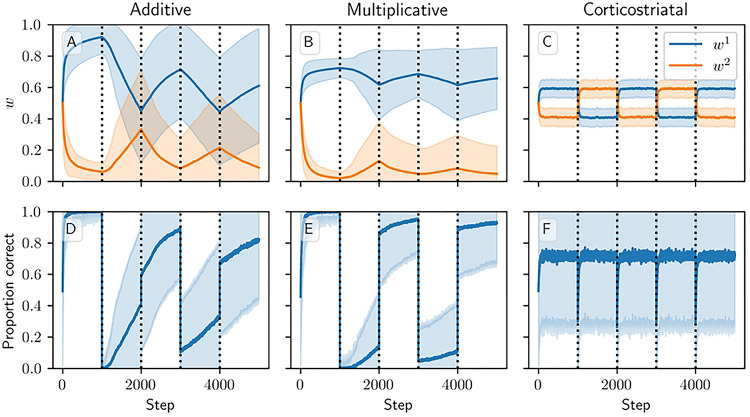
Model performance in the action selection setting with contingency switching. Plots show weights (A-C) and probability of taking the correct action (D-F) versus time for the additive (A, D), multiplicative (B, E), and corticostriatal (C, F) models. Here $r=10{\;s}^{-1}$ and the reward contingencies switch between $R_{1}^{*}=2,$ $R_{2}^{*}=1$ and $R_{1}^{*}=1,$ $R_{2}^{*}=2$ every 1000 steps; we use λ=0.05 for illustrative purposes.

Fig 7 and S1 Fig show results for learning of a single relation between action and reward. In some situations, both in experiments and in natural settings, relations between actions and subsequent rewards can change over time, an effect that we refer to as contingency switching. To simulate these tasks, we swap which action is mapped to the higher reward value every 1000 steps. In this situation, we find that substantial differences arise in performance among the three models. Fig 8 shows that the additive and multiplicative models are unable to perform these tasks well, because the weights get stuck near the widely spread values that they attain for the first contingency scenario. Running the simulations with longer intervals between switches would not help as the weights take just as much time to escape from these values as they spend approaching them; that is, longer intervals lead to stronger convergence and hence more time needed to move away after a contingency switch. The corticostriatal model, in contrast, is able to quickly react to the contingency switches and swap which action it takes, resulting in only brief drops in accuracy when switches occur.

We also tested model performance in task switching in the action selection task. S2 Fig shows model trajectories and proportion of trials on which the more rewarding action is chosen under slow switching, where the inputs and rewards are swapped every 1000 steps. All three models are able to switch which action they take when the state switches. But whereas the additive and multiplicative models are able to learn a set of weights that can yield high probabilities of selection of the more rewarded action in both states, the corticostriatal model struggles to do so because of the more limited range of values the weights take under its dynamics. The corticostriatal model is able to recover its prior performance after a task switch, but it does not seem to learn one set of weights that have above-chance performance on both tasks. When switching is fast (every step), the corticostriatal model learns weights that give performance only slightly better than chance, while the additive and multiplicative models successfully learn weights that perform well in both states (see S3 Fig).

### Single Eligibility Trace

We now revisit the question of whether to use a single eligibility trace summing up both positive (corresponding to pre-before-post spike pairs) and negative (post-before-pre) contributions, as is done in [31], or to use two different traces for the positive and negative components, as we do above. We test a single-trace version of our model that replaces Eq (3) with

(11) $$\frac{dE_{i}}{dt}=\rho^{post}(t)A_{i}^{pre}(t)-\gamma \rho_{i}^{pre}(t)A^{post}(t)-\frac{1}{\tau_{eli}}E_{i}(t)$$

where γ≥1 is a scaling parameter controlling the strength of negative eligibility terms relative to positive terms. The single-trace differential equation for the weights in the additive and multiplicative cases is given by

(12) $$\frac{dw_{i}}{dt}=\left\{\begin{array}{ll} \lambda D(t)f_{+}\left(w_{i}(t)\right)E_{i}(t) & \text{if}\;E_{i}(t)\geq 0\\ \lambda D(t)f_{-}\left(w_{i}(t)\right)E_{i}(t) & \text{if}\;E_{i}(t)<0. \end{array}\right.$$

and for the corticostriatal model is given by

$$\frac{dw_{i}}{dt}=\left\{\begin{array}{ll} \lambda D(t)\left(1-w_{i}(t)\right)E_{i}(t) & \;\text{if}\;D(t)E_{i}(t)\geq 0\\ \lambda D(t)\alpha w_{i}(t)E_{i}(t) & \;\text{if}\;D(t)E_{i}(t)<0. \end{array}\right.$$

The single-trace version of the corticostriatal model is largely equivalent to the model in described in [31], although they use different scaling factors and time constants for pre- and postsynaptic activity.

We had several reasons for focusing in this paper on models with two different eligibility traces. One was analytical convenience: the use of two traces is necessitated by the assumption, made by [28, 30] as well as in the previous sections, that the contributions to the weight changes made by individual spike pairs sum independently, a natural assumption that greatly simplifies analysis. With only one eligibility trace, different spike pairs may cancel each other out, rendering this independence assumption invalid. We therefore cannot derive averaged forms of the single-trace models like we did for the two-trace models. A second justification for the focus on two-trace models is that there is experimental evidence suggesting that the brain in fact uses two different traces, one for LTP and one for LTD [34]. These findings describe cortical pyramidal cells, rather than corticostriatal synapses, but similar mechanisms may be at play here too.

One important characteristic of the single-trace versions of the additive and multiplicative models (Eq (12)) is that they are largely insensitive to variations in α. This insensitivity arises because $E_{i}(t)$ is usually positive, since presynaptic spikes directly cause postsynaptic spikes after a delay of ϵ and not vice versa, which tilts the balance to favor positive eligibility. Hence, the α-dependent $f_{-}$ term is only rarely used. The α parameter is therefore not an effective way of adjusting the relative strengths of the positive and negative components of the eligibility trace. This observation motivates the introduction of the parameter γ in Eq (11) to provide a better means of controlling the relative strengths of the two components in the single-trace models. (The single-trace corticostriatal model is still sensitive to α because it depends on the sign of the product $D(t)E_{i}(t)$, rather than just $E_{i}(t)$, so the term with α will have an impact when $E_{i}(t)>0$ and D(t)<0.)

**Fig 9.**
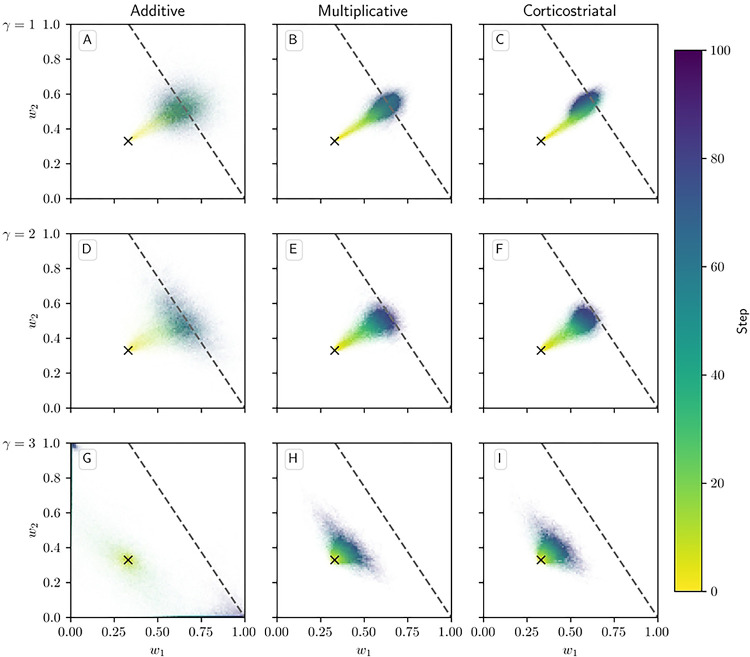
Distribution of weights over time in the reward prediction setting as γ is varied for single-trace models. Columns show the additive (A, D, G), multiplicative (B, E, H), and corticostriatal (C, F, I) models. γ is varied across rows: (A-C) γ=1; (D-F) γ=2; (G-I) γ=3. We include the solution planes for reference, but as we do not have averaged forms of the single-trace dynamics we do not include vector fields or fixed points or analyze the stability of the solution planes.

We show simulations of the single-trace models in the random dopamine, reward prediction, and action selection settings in S4 Fig, Fig 9, and S5 Fig, as well as for action selection with contingency switching in S6 Fig. In the random dopamine setting (S4 Fig) we vary γ in addition to α; in the reward prediction setting (Fig 9) we vary γ and keep α=1 fixed. The single-trace and two-trace versions of the additive model behave identically when α=γ=1, because in this case positive and negative eligibility are treated the same. In the random dopamine setting all three models behave qualitatively similarly to the two-trace versions (Fig 3), and they appear largely insensitive to γ. In the action selection setting results again qualitatively match those found with the two-trace models (Figs 7 and 8). In the reward prediction setting, on the other hand, some differences between single-trace and two-trace model dynamics are visible (cf. Fig 4), especially for larger values of γ. While the solution planes become increasingly unstable as γ increases, similar to the effect seen in the two-trace models as *α* increases, the precise form of the dynamics appears to differ considerably (e.g. in Fig 9H, trajectories seem to spread out rather than converge to a fixed point under the multiplicative model). Overall, using a single eligibility trace does not seem to significantly improve performance on these tasks and makes the dynamics much more difficult to analyze.

### Symmetric Model

None of the models we have considered can accomplish every task we set for them. Can we use our findings to design a plasticity model that can? Here we consider one possibility. Rather than switching whether we scale weight changes by w or 1-w depending on pre-post spike timing, as we do in the multiplicative and corticostriatal models, we simply use w(1-w) irrespective of the direction of the weight update; in other words, rather than equations (4) or (5) we use

$$\frac{dw_{i}}{dt}=\lambda D\left(t\right)w_{i}\left(t\right)\left(1-w_{i}\left(t\right)\right)\left(E_{i}^{+}\left(t\right)-E_{i}^{-}\left(t\right)\right).$$

We will refer to this model as the *symmetric model*. This model fixes the issue with the multiplicative model where w may be used when weights are increasing and 1-w used when weights are decreasing, which may occur if the dopamine signal is negative. Moreover, the dopamine signal can be factored out of the update equation for the symmetric model, unlike with the corticostriatal model; it is therefore to be expected that the symmetric model will perform well in the random dopamine and reward prediction settings where the corticostriatal model does poorly.

**Fig 10.**
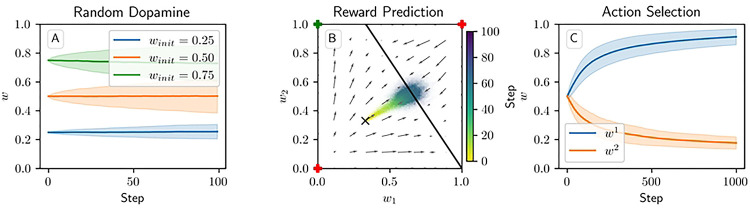
Weight evolution in the three main settings for the symmetric model. This model requires the use of relatively large values of λ: (A) in the random dopamine setting, λ=0.02; (B) in the reward prediction setting, λ=0.0066; (C) in the action selection setting, λ=0.05.

**Fig 11.**
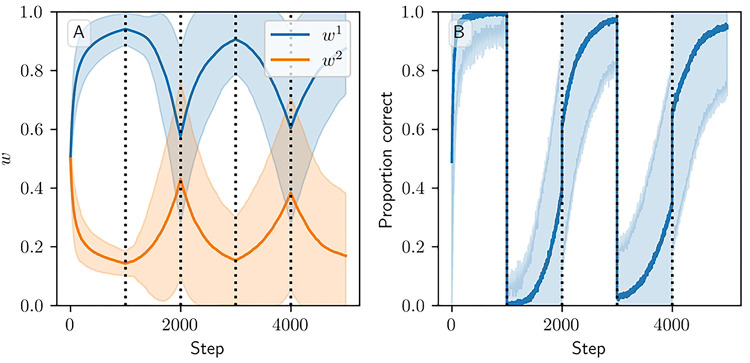
Performance of the symmetric model in the action selection setting with contingency switching. Plots show weights (A) and probability of taking the correct action (B) versus time. Here λ=0.1, and the other parameters are the same as those used in Fig 8.

Experimentally, we see that the symmetric model maintains the good performance of the additive and multiplicative models in the random dopamine and reward prediction settings as well as the basic action selection setting (Fig 10). However, it does not do as well as the corticostriatal model in the action selection setting with slow contingency switching. In this setting the weights for the corticostriatal model converge to fixed points some distance from the boundaries and contingency switching seems to swap the locations of the stable fixed points, allowing the model to respond quickly to switches (see Fig 8C). Under the symmetric model, weights still get driven towards the boundaries. While they go to the boundaries much more slowly than under the additive and multiplicative models due to the w(1-w) term, they also take correspondingly longer to leave once they get there (Fig 11). So while the symmetric model may be an improvement over the additive and multiplicative models in some ways, it does not seem to provide a panacea for the other models’ shortcomings.

## Discussion

Accurately modeling learning in the cortico-basal ganglia-thalamic circuit requires the use of an appropriate synaptic weight update rule for the dopamine-dependent STDP in the corticostriatal connections. In this paper we examine three plasticity models that combine dopamine, eligibility, and spike timing signals in different ways – the additive, multiplicative, and corticostriatal models – and evaluate their performance in a number of different task settings. We find that the additive and multiplicative models do well in many cases: they are able to maintain weights in the presence of random inputs and dopamine release events, they can learn to predict a reward, and they can accomplish a simple action selection task. They do not perform well on action selection tasks in which the reward contingencies occasionally switch, however, because they tend to get stuck at or near the boundaries of the weight domain. In contrast, the corticostriatal model, while performing poorly in the random dopamine and reward prediction settings, is able to rapidly relearn swapped reward contingencies in the action selection setting. This rapid learning matches the results seen in experiments with animals [61] and humans [49]. When tasks instead of contingencies switch, however, the success of the corticostriatal model is hindered somewhat by the restricted range of synaptic weight values that it induces. Overall, we find that the choice of which plasticity model to use can have a large impact on the dynamics of synaptic weights and hence on both the learning achieved by the circuit and the ability of the model to perform a given task. Which plasticity model is appropriate depends strongly on the tasks it will be asked to perform. Ultimately, these results suggest that different synaptic plasticity mechanisms may be at play at corticostriatal synapses involving different regions of the striatum with distinct functions, and that additional experimental and theoretical work is needed to pin down the precise forms of plasticity that occur at corticostriatal synapses and how they should be modeled.

Our mathematical analysis of the random dopamine and reward prediction settings shows how the choice of parameter values impacts model performance on these tasks. Specifically, we found that in the random dopamine setting that under the corticostriatal model, weights evolve to 1/(α+1); in the reward prediction setting under the additive and multiplicative models, we characterized how the existence of stable points on the solution plane (and therefore the ability of the model to solve the task) depends on the parameters α, τ, r, and $R^{*}$. In general, increasing α, the strength of negative eligibility relative to positive, and τ, the STDP time constant, will reduce the ranges of r and $R^{*}$ values that feature stable solutions. Therefore, these parameters are particularly important for practitioners using these models to understand and to select judiciously.

Why exactly do the three plasticity models run into difficulties in some settings? An important issue with the additive model is that it does not prevent weights from being driven to the boundaries (or past them, if the weights are not artificially cut off). The original multiplicative model without dopamine avoids this complication by scaling the weight drift by w if weights are decreasing and by 1-w if they are increasing, but our version of the model with dopaminergic modulation disrupts this property; because the w and 1-w terms are tied to the sign of the eligibility but not the sign of the dopamine signal, if the dopamine signal is negative, then the wrong term is applied (w for increasing weights and 1-w for decreasing weights). This effect can lead to weights being driven to zero in the action selection setting. The corticostriatal model solves this problem by selecting w or 1-w depending on the sign of the dopamine signal times the eligibility trace; in other words, it ensures that even with dopamine the correct scaling term will be chosen: w for decreasing weights and 1-w for increasing weights (see Table 1). The cost of this modification, from an analytical perspective, is that the dopamine signal can no longer be factored out of the weight drift equation. Consequently the corticostriatal model features nonzero mean weight drift even when the mean dopamine signal is zero, leading to its failure to maintain pre-learned weights under random dopamine and its failure to converge to the solution plane in the reward prediction setting. Recent experimental results on local control of dopamine release within the striatum [62–64] suggest that neurons may express more complicated mechanisms that we have not modeled that allow them to avoid spurious weight changes when not involved in task performance, which may ameliorate the difficulties of the corticostriatal model in the random dopamine setting. On the theoretical side, we introduced the symmetric model considered in Results, Symmetric Model as an attempt to have the best of both worlds: a model that properly scales weight updates near the boundaries while allowing the dopamine signal to be factored out of the weight drift equation. Unfortunately, it does not significantly improve on the poor performance of the additive and multiplicative models in the action selection setting with contingency switching.

The corticostriatal model has another problem: its weights tend to remain in a relatively narrow band, leading to a fairly low probability of taking the correct action in the action selection setting. This probability is determined by the number of postsynaptic spikes in each channel, and if the weights are close together, then spiking noise will sometimes lead to more spikes being counted in the incorrect channel, causing the wrong action to be taken. This outcome occurs despite the fact that we use a large value of β, the temperature parameter in our action selection probability function. We believe that this problem is not a fundamental one, however, as it can be easily solved through downstream integration over the outputs of multiple striatal neurons to obtain a clearer signal.

One important issue that we have highlighted throughout this work is the impact that delays have on the weight dynamics. In the reward prediction setting, we need to ensure that there is a sufficiently long delay between when we estimate the postsynaptic firing rate and when dopamine is actually delivered; without this delay, correlations between terms prevent the weights from converging to the solution plane. On the other hand, we need to use short delays in the action selection setting without sustained activity in the selected channel, because these correlations are required for the model to learn which action to take. A complicating factor that we have not addressed is that experimental results consistently show that dopamine release immediately upon pre-post spike pairing does *not* lead to a change in weight; rather, the dopamine must come some time after the spiking activity to effect significant synaptic changes [58, 59]. These findings raise important questions about how to best understand and model delays within a synaptic plasticity framework. Although we considered the eligibility trace as jumping up immediately upon the occurrence of a spike pair and then decaying exponentially, for the sake of analytical tractability and for consistency with prior computational work, an important extension of these results would be to represent the eligibility trace as slowly ramping up and then ramping down over time and to study its interaction with delays and the computational role that it plays.

Our additive and multiplicative models are based on the plasticity rules described in [30], but our plasticity rules differ from theirs in that we incorporate dopaminergic modulation of the synaptic plasticity. The random dopamine setting is closest to the one they use, and indeed by fixing the mean dopamine level to some positive constant (rather than drawing it from a normal distribution centered at zero) we can reproduce their setting very closely, the only difference being that our model only undergoes plasticity only during the periodic dopamine signals rather than after every spike pair. Our goals are quite different from those of the earlier work, however; while they study conditions under which symmetry breaking in the weight distributions occurs and when the models can learn to represent correlations in a set of inputs, we instead use the random dopamine setting to investigate the stability of learned weights under perturbation.

The corticostriatal model in this paper is based on the plasticity model used in [31] but differs from their model in a number of important ways. We make several simplifications to the model, including setting the scaling factors and time constants for pre- and postsynaptic spike traces equal to each other (in their notation, $\tau_{PRE}=\tau_{POST}$ and $\Delta_{PRE}=\Delta_{POST}=1$), as well as considering a single class of striatal neurons rather than taking into account the existence of multiple striatal neuron subpopulations with different plasticity properties. They also employ their plasticity model in a more biologically realistic setting, incorporating many components of the basal ganglia circuitry that we leave out. The most interesting difference between our models is that they use a single eligibility trace summing up both positive (corresponding to pre-before-post spike pairs) and negative (post-before-pre) contributions, while we use two different traces for the positive and negative components. The use of two traces is justified by experimental evidence suggesting that the brain uses two distinct eligibility traces for LTP and LTD [34]. (Note, however, that the computational model introduced in [34] differs considerably from the models used here, as it does not use αw or 1-w factors to rescale the positive and negative traces, instead simply adding them together unmodified.) We find in Results, Single Eligibility Trace that modifying our models to employ a single eligibility trace leads to qualitatively similar results in most cases, although they are much more difficult to analyze.

A number of other three-factor plasticity rules have been explored in the literature. One important model can be found in [65]; while our learning rules are generally built off of simpler two-factor rules modified to incorporate dopaminergic feedback, they derive their learning rule directly from gradient ascent applied to a reward signal. Another work modeling dopamine-dependent STDP is [66]. Their plasticity rule closely resembles the additive model. However, while we focus on the corticostriatal synapses and employ a simple setting consisting of a population of cortical neurons connected to a single striatal neuron, they instead use a mixed population of excitatory and inhibitory neurons with random connectivity meant to model part of a cortical column. The scenarios that they use to test their model also differ from ours. For a more detailed review of other work on three-factor plasticity rules, see [2, 3].

What are the implications of our findings for models of the basal ganglia? We showed that each model has some settings in which it does well and some settings where it fails to accomplish the given task. There are several potential explanations for these outcomes. It is possible that the plasticity mechanism used in the corticostriatal synapses incorporates features that are not well-captured by any of the models considered here. It is also possible that the simplified models that we consider omit aspects of the computational structure of the basal ganglia that are crucial for functional performance. For instance, we do not model the competition between direct and indirect pathways through the basal ganglia, nor the differing effects of dopamine on the two pathways (spiny projection neurons in the direct pathway primarily express the D1 receptor, for which higher dopamine levels lead to LTP and lower dopamine levels lead to LTD and which form the basis for the corticostriatal plasticity model considered here, while in the indirect pathway they primarily express the D2 receptor, for which higher dopamine levels lead to LTD and lower dopamine levels lead to LTP [35, 36]). There may also be more complexity to dopaminergic feedback than the simple model we use; for example, recent work suggests that the dopamine signal may be better modeled as multidimensional rather than scalar-valued [67]. An exciting future direction would be to extend our analysis to take more of these subtleties into account. Nevertheless, we believe that the settings we studied are general enough to provide lessons applicable to many more detailed models.

An interesting possible implication of our work is that different regions of the striatum may feature different plasticity mechanisms specialized to their particular roles. For instance, the ventral and dorsal striatum, which primarily contribute to reward prediction and action selection, respectively [68], may use distinct plasticity rules tuned to the specific tasks that they perform, as suggested by experimental evidence [14, 16]. More generally, while we have focused in this paper on the corticostriatal connections, our settings are broad enough that they may apply to any other region of the brain that receives dopaminergic signals. The random dopamine setting should be relevant whenever the dopamine signal is independent of a neuron’s output, the reward prediction setting applies to any task in which a neuron must match a target firing rate in order to minimize a dopamine error signal, and the action selection setting is a fairly broad model of learning dynamics under competition between two channels. Thus, the fact that no plasticity rule performed well in every setting may simply be due to the specialization of different regions for the specific computational functions that they perform.

## Supplementary Material

Supplement 1

## Figures and Tables

**Table 1.** Scaling factors for the three main models for positive and negative dopamine and eligibility.

	Additive		Multiplicative		Corticostriatal	
$E_{i}(t)$╲D(t)	−	+	−	+	−	+
−	*α*	*α*	*αw*	*αw*	1 — *w*	*αw*
+	1	1	1 — *w*	1 — *w*	*αw*	1 — *w*

Blue cells correspond to scenarios in which the weights will increase, while orange indicates that the weights will decrease.

**Table 2.** Default simulation parameters for the three main task settings.

	Random Dopamine	Reward Prediction	Action Selection

Samples	1000	1000	1000
Steps	100	100	1000
λ	0.01	0.0033	0.025
$w_{\text{init }}$	0.5	0.33 or 0.5	0.5
*N*	1	2	1
*r*	5 s^−1^	(15, 10) s^−1^	10 s^−1^
$R^{*}$	N/A	7.5	N/A
$R_{1}^{*},$ $R_{2}^{*}$	N/A	N/A	(2, 1)
α	1	1	1
τ	0.02 s	0.02 s	0.02 s
$\tau_{\text{dop }}$	1 s	1 s	1 s
$\tau_{\text{eli }}$	1 s	1 s	1 s
$T_{\text{del }}$	N/A	3 s	0 s
$T_{\text{win }}$	N/A	1 s	1 s
ϵ	0.001 s	0.001 s	0.001 s
$r^{\text{dop }}$	1/6 s^−1^	1/7 s^−1^	1/7 s^−1^
β	N/A	N/A	10^5^
$\sigma_{\text{dop }}$	1	N/A	N/A

fixed points in the sense that they are not zeros of the dynamical equations. Rather, they are the result of the weights being clipped at 0 and 1.
