# Distinct dopaminergic spike-timing-dependent plasticity rules are suited to different functional roles

**DOI:** 10.1101/2024.06.24.600372

**Published:** 2024-10-04

**Authors:** Baram Sosis, Jonathan E. Rubin

**Affiliations:** 1 *Department of Mathematics, University of Pittsburgh, 301 Thackeray Hall, Pittsburgh, 15260, PA, USA.; 2 Center for the Neural Basis of Cognition, University of Pittsburgh, 4400 Fifth Ave, Pittsburgh, 15213, PA, USA.

**Keywords:** Dopamine, Synaptic plasticity, STDP, Basal ganglia, Reward prediction, Action selection

## Abstract

Various mathematical models have been formulated to describe the changes in synaptic strengths resulting from spike-timing-dependent plasticity (STDP). A subset of these models include a third factor, dopamine, which interacts with spike timing to contribute to plasticity at specific synapses, notably those from cortex to striatum at the input layer of the basal ganglia. Theoretical work to analyze these plasticity models has largely focused on abstract issues, such as the conditions under which they may promote synchronization and the weight distributions induced by inputs with simple correlation structures, rather than on scenarios associated with specific tasks, and has generally not considered dopamine-dependent forms of STDP. In this paper we introduce three forms of dopamine-modulated STDP adapted from previously proposed plasticity rules. We then analyze, mathematically and with simulations, their performance in three biologically relevant scenarios. We test the ability of each of the three models to maintain its weights in the face of noise and to complete simple reward prediction and action selection tasks, studying the learned weight distributions and corresponding task performance in each setting. Interestingly, we find that each plasticity rule is well suited to a subset of the scenarios studied but falls short in others. Different tasks may therefore require different forms of synaptic plasticity, yielding the prediction that the precise form of the STDP mechanism present may vary across regions of the striatum, and other brain areas impacted by dopamine, that are involved in distinct computational functions.

## 1 Introduction

Learning and memory are critical features of cognition in both humans and non-human animals, and a number of neural learning mechanisms have been described. One important mechanism is spike-timing-dependent plasticity (STDP) Markram et al. (1997); Bi and Poo (1998), a class of Hebbian plasticity rules in which the relative timing of pre- and postsynaptic spikes determines the changes in synaptic connection strength. Typically, a presynaptic spike before a postsynaptic spike – that is, a causal ordering of the spikes – leads to synaptic potentiation, whereas the reverse order leads to depression. In many cases, though, synaptic plasticity depends not just on the timing of pre- and postsynaptic spikes but also on some third factor, such as a neuromodulatory signal or other input Frémaux and Gerstner (2016); Gerstner et al. (2018). These additional factors may act as gating signals, and their strength and timing may impact both the magnitude and the direction of synaptic changes.

A prominent example of neuromodulatory impact on synaptic plasticity occurs at the cortical inputs to the basal ganglia. The neuromodulator dopamine is released by midbrain dopamine neurons when unexpected reward is received Schultz (1998); Schultz and Romo (1990) and plays a crucial role in modulating plasticity of corticostriatal synapses Surmeier et al. (2010). Experimental evidence and theoretical modeling suggest that dopamine serves as a reward prediction error signal Montague et al. (1996); Schultz et al. (1997), enabling the brain to learn to favor behaviors that lead to reward and disfavoring behaviors that do not; such findings have been recently reviewed Lerner et al. (2021). Theoretical analysis of action selection and modulation by cortico-basal ganglia-thalamic (CBGT) circuits posits a role for these dopaminergic reward prediction error signals both in updating value estimates associated with available choices and, through their impact on corticostriatal synaptic strengths, in altering the likelihood that a particular action will be selected in the future Gurney et al. (2015); Mikhael and Bogacz (2016); Baladron and Hamker (2020); Vich et al. (2020). These distinct functions are likely performed by different neurons in different regions of the basal ganglia, however, which raises the possibility that distinct plasticity rules are involved. Unfortunately, despite some exciting experimental investigations of long-term plasticity properties in specific striatal regions and task settings Perez et al. (2022); Smith et al. (2001); Wang (2008), relatively little is known about the details of these plasticity mechanisms, especially in striatal regions thought to encode value.

**Fig. 1:**
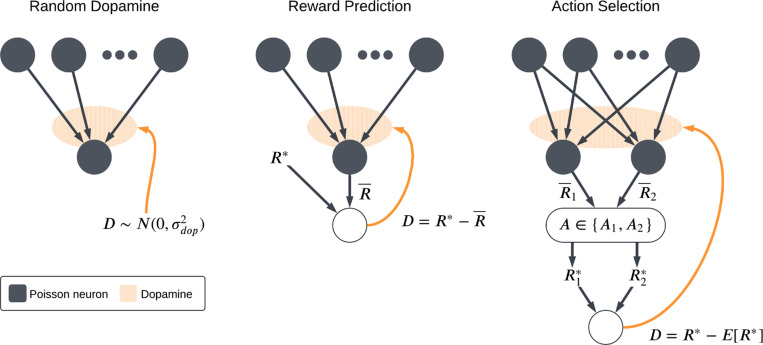
Schematic of the three main task settings. In the random dopamine setting, the neuron of interest receives stochastic cortical inputs not related to its primary function and dopamine signals resulting from activity elsewhere in the basal ganglia. In the reward prediction setting, the output firing rate is interpreted as a predicted reward and the dopamine signal is the reward prediction error. In the action selection setting, an action is chosen based on which of two competing channels has a higher output firing rate. A reward is then received based on the action taken and the dopamine again represents reward prediction error

These considerations lead to the question of how well particular implementations of dopaminergic plasticity can perform the kinds of tasks or fulfill the kinds of roles that the corticostriatal system is believed to execute in the brain. If a plasticity model is capable of supporting biologically-relevant tasks, then that serves as some evidence in favor of the model; conversely, if it fails to do so, then we may want to modify it or look for other alternatives. While many analyses of different reward-modulated learning rules have been performed Izhikevich (2007); Xie and Seung (2004); Legenstein et al. (2008); Porr et al. (2007); Frémaux et al. (2010), prior work has generally focused on particular sets of tasks or particular classes of plasticity models, rather than examining the range of tasks that the striatum may have to perform and which plasticity rules are best suited for which tasks. To fill this gap, we describe three models of dopaminergic plasticity, two derived by extending more general models of STDP learning to incorporate dopaminergic modulation and one designed specifically to model corticostriatal plasticity, as well as some variations on these models. We consider their performance in several different task settings relevant to the striatum, illustrated in Figure 1. As a baseline, we study synaptic weight evolution in a neuron receiving random, uncorrelated inputs and dopamine; this is meant to model a neuron uninvolved in whatever task the animal is performing. We also study simple models of reward prediction and action selection, two tasks in which the basal ganglia are believed to play major roles Schultz et al. (1998); Surmeier et al. (2009); Chakravarthy et al. (2010); Kravitz and Kreitzer (2012); Grillner et al. (2013); Hikosaka et al. (2014); Orsini et al. (2015); Mikhael and Bogacz (2016); Mink (2018). Finally, we examine some more complex variants of these settings in which the reward contingencies or the task changes periodically. We find that although each model does well in some, no model is able to succeed in all of the scenarios we consider. Thus, our results suggest that the brain may need to employ distinct, specialized plasticity mechanisms to learn different tasks.

## 2 Models

### 2.1 Plasticity Models

Here we introduce three models of dopaminergic spike-timing-dependent plasticity. The *additive* and *multiplicative models* are based on incorporating dopamine into existing models of STDP (without dopamine) described in Abbott and Blum (1996); Gerstner et al. (1996) (for the additive model) and Kistler and Hemmen (2000); Rubin et al. (2001); van Rossum et al. (2000) (for the multiplicative model); we mainly follow the presentation in Gütig et al. (2003). What we call the *corticostriatal model* is based on a computational model of the corticostriatal connections, specifically connections onto striatal spiny projection neurons that express the D1 dopamine receptor, sometimes referred to as direct pathway SPNs, as described in Clapp et al. (2024). This model incorporates recent experimental findings about synaptic plasticity and eligibility traces in these neurons Richfield et al. (1989); Shen et al. (2008); Dreyer et al. (2010); Keeler et al. (2014); Shan et al. (2014); Fisher et al. (2017); Shindou et al. (2019) and builds on other recent modeling studies Gurney et al. (2015); Mikhael and Bogacz (2016); Baladron and Hamker (2020); Vich et al. (2020).

We consider linear Poisson neurons: presynaptic spike trains are modeled as Poisson processes $\rho_{i}^{\text{pre}}(t)$ with constant rate $r_{i}={\langle \rho_{i}^{\text{pre}}(t)\rangle }_{t}\;(\text{where}\;i=1,2,\ldots ,N)$, and likewise spike trains of the single postsynaptic neuron are Poisson processes $\rho^{\text{post}}(t)$ with instantaneous firing rate functions R(t) given by a linear combination of the presynaptic spike trains:

(1) $$R(t)=\frac{1}{N}\sum_{i=1}^{N}w_{i}(t)\rho_{i}^{\text{pre}}(t-\epsilon )$$

where ϵ>0 is a small synaptic delay and $w_{i}$ are the synaptic efficacies, which we will also call weights, which we normalize to [0, 1]. (We will write the vectors of input firing rates and weights as $r,w\in {\mathbb{R}}^{N}$.) This can be implemented by first generating input spike trains $\rho_{i}^{\text{pre}}(t)$, and then, whenever a presynaptic spike from input unit i occurs, say at time $t_{\text{pre}}$, adding a postsynaptic spike at time $t_{\text{post}}=t_{\text{pre}}+\epsilon$ to the postsynaptic spike train $\rho^{\text{post}}$ with probability $\frac{1}{N}w_{i}\left(t_{\text{pre}}\right)$. We assume that the input spike trains are uncorrelated.

Rather than modifying each synapse immediately with the occurrence every spike pair, as in a classical two-factor STDP rule, we instead assume that an eligibility trace Houk et al. (1994); Sutton and Barto (2018) for that synapse is incremented, which decays exponentially between spike pairs. Then the weight change is proportional to both the current value of the eligibility trace and the value of the dopamine signal, described below.

We base our implementation of this model on the implementation described in Vich et al. (2020) and use a set of trace variables to track the influences of pre- and postsynaptic spikes and spike pairs. We define $A_{i}^{\text{pre}}(t)$ and $A^{\text{post}}(t)$ to track the pre- and postsynaptic spiking:

(2) $$\frac{dA_{i}^{\text{pre}}}{dt}=\rho_{i}^{\text{pre}}(t)-\frac{1}{\tau }A_{i}^{\text{pre}}(t)\;\frac{dA^{\text{post}}}{dt}=\rho^{\text{post}}(t)-\frac{1}{\tau }A^{\text{post}}(t)$$

with decay constant τ>0. We also define eligibility traces to track spike pairs. An important assumption in our analysis, made by Gütig et al. (2003); Rubin et al. (2001) and others, is that changes in weight from individual spike pairs can be summed independently. To realize this, we define two eligibility traces, $E_{i}^{+}(t)$ and $E_{i}^{-}(t)$, to track pre-before-post and post-before-pre spike pairs, respectively:

(3) $$\frac{dE_{i}^{+}}{dt}=\rho^{\text{post}}(t)A_{i}^{\text{pre}}(t)-\frac{1}{\tau_{\text{eli}}}E_{i}^{+}(t)\;\frac{dE_{i}^{-}}{dt}=\rho_{i}^{\text{pre}}(t)A^{\text{post}}(t)-\frac{1}{\tau_{\text{eli}}}E_{i}^{-}(t)$$

with decay constant $\tau_{\text{eli}}>0$. We use two independent traces in part because experimental results have suggested that this independence is present in cortical synapses He et al. (2015). Moreover, using a single trace, as done previously Vich et al. (2020), allows spike pairs to interact nonlinearly and partially cancel each other out, while using two traces ensures that different spike pairs do not interact, which is convenient for analysis. In Section E we show that using a modified plasticity model with a single eligibility trace gives qualitatively similar results in most cases, and does not meaningfully improve performance on the tasks we study here.

We assume that dopamine is released at fixed intervals of length $1/r^{\text{dop}}$ for constant $r^{\text{dop}}>0$; otherwise, it decays exponentially:

$$\frac{dD}{dt}=\sum_{k}D_{k}\delta \left(t-k/r^{\text{dop}}\right)-\frac{1}{\tau_{\text{dop}}}D$$

The value of the dopamine increment $D_{k}$ depends on the task setting; see Section 2.2. (We will treat this signal as the dopamine level *relative to some baseline*, rather than the raw dopamine concentration itself; so, in the absence of any signal, D equals zero, and we allow $D_{k}<0$.) Note that while the dopamine *concentration* may depend on the postsynaptic spike train, we assume for analytical convenience that the *timing* of dopamine delivery is independent of the spiking activity. Here we assume the dopamine is simply delivered periodically for simplicity; the precise form of the dopamine process is irrelevant as long as it has mean rate $r^{\text{dop}}$, is independent of the spike trains, and yields dopamine signals that are far enough apart that their interactions can be neglected.

Finally, the weights in equation (1) are updated using the values of the dopamine signal and the eligibility traces in a way that depends on the choice of plasticity model.

The additive and multiplicative models use the following rule:

(4) $$\frac{dw_{i}}{dt}=\lambda D(t)\left(f_{+}\left(w_{i}(t)\right)E_{i}^{+}(t)-f_{-}\left(w_{i}(t)\right)E_{i}^{-}(t)\right)$$

where $f_{+}(w)={\left(1-w\right)}^{\mu }$ and $f_{-}(w)=\alpha w^{\mu }$ apply different scaling factors to weight updates from positive and negative eligibility. λ>0 is the learning rate, α tunes how strongly negative eligibility is weighted relative to positive eligibility (typically α≥1), and μ∈[0,1] selects from a family of different possible update functions. We will only consider the cases μ=0, known as the additive model, and μ=1, known as the multiplicative model. (See Gütig et al. (2003) for an exploration of the effects of varying μ in a simpler two-factor STDP setting.)

The corticostriatal model is broadly similar, but modifies the functional form of the weight update depending on the sign of the weight change. Rather than using the $f_{+}/f_{-}$ functions defined above, we use f(w)=1−w when the sign of the overall weight change (including the sign of the dopamine signal and the sign of the eligibility) is positive, and f(w)=αw when it is negative. This convention is described by the formula:

(5) $$\frac{dw_{i}}{dt}=\{\begin{array}{cc} \lambda D(t)\left(\left(1-w_{i}(t)\right)E_{i}^{+}(t)-\alpha w_{i}(t)E_{i}^{-}(t)\right) & \text{if}\;D(t)\geq 0\\ \lambda D(t)\left(\alpha w_{i}(t)E_{i}^{+}(t)-\left(1-w_{i}(t)\right)E_{i}^{-}(t)\right) & \text{if}\;D(t)<0 \end{array}$$

In all three models, synapses become stronger with above-baseline dopamine signals (and weaker with below-baseline dopamine signals) when the postsynaptic neuron has recently participated in a pre-before-post spike pairing, and weights change in the opposite direction following post-before-pre spike pairs. These properties are implemented to match the observed behaviors of cortical synapses onto striatal spiny projection neurons expressing specifically D1 dopamine receptors Gurney et al. (2015); Baladron and Hamker (2020); Vich et al. (2020); Clapp et al. (2024); Shen et al. (2008); Shan et al. (2014). Neurons expressing D2 receptors show the opposite behavior, but we do not consider those here.

Table 1 shows how the scaling factors used by each of the three models depends on the signs of the dopamine and eligibility. The additive and multiplicative models only depend on the sign of the eligibility, while the corticostriatal model uses the sign of the product of dopamine and eligibility to determine which scaling factor to use. This means that when using the corticostriatal model, the scaling factor corresponds to the direction in which the weights will change: 1−w for increasing weights and αw for decreasing weights. In contrast, the scaling factors used by the additive and multiplicative models do not correspond to the direction of weight change.

While we primarily focus on the additive, multiplicative, and corticostriatal models in this paper, we will also explore some variations on these models. In Section E we describe versions of these three models using a single eligibility trace, rather than the two traces we use elsewhere. In Section 3.4 we also explore a new model, which we term the *symmetric model*, meant to alleviate some of the issues we find with the other three plasticity rules.

For all of the models, weights are kept bounded between 0 and 1; for the additive and multiplicative models, this necessitates clipping weights that would go beyond these limits based on equation (4) alone.

### 2.2 Task Settings

The plasticity models described above are agnostic as to how exactly the dopamine signal is computed. We consider three different task settings, corresponding to three different scenarios or functional roles that may arise with striatal neurons (see Figure 1). The first is what we will refer to as the *random dopamine setting*: dopamine is sampled from a normal distribution centered at zero, $D∼N\left(0,\sigma_{\text{dop}}^{2}\right)$, independently of the spiking activity. This models a neuron that is uninvolved in whatever task the animal is performing; it may receive some spurious inputs and dopamine due to activity elsewhere, but its inputs and output are statistically independent of the dopamine release. We would like a plasticity model that yields zero net weight drift under random dopamine, so that previous learning is not erased. While the stochastic inputs and dopamine may perturb the weights somewhat, ideally it should not cause them to move consistently in one direction or another.

**Fig. 2:**
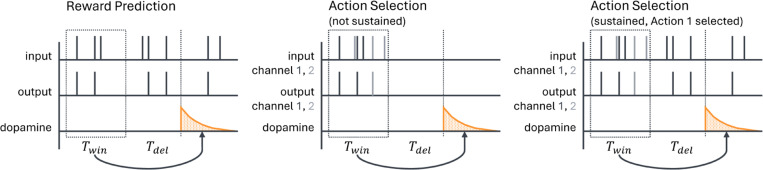
Schematic of the sequence of events in the reward prediction and action selection models. Output spikes are counted in a window of length $T_{\text{win}}$ to estimate the average output firing rate; then, after a delay of length $T_{\text{del}}$ (which may be zero), dopamine is released. For the action selection model there are two channels, colored black and gray, corresponding to the two actions being considered. We examine two versions of the action selection model: one in which the cortical input is suppressed outside of the spike count window, and one in which activity is maintained in the selected channel (here channel 1). These two variants are most similar when $T_{\text{del}}=0$ (although they still differ due to spikes occurring after dopamine is released), and we often will consider that case, but we will also compare it to results with $T_{\text{del}}>0$ as shown in the figure

The second scenario that we will consider is the *reward prediction setting*. In this model, we assume that the dopamine signal takes the form of an error signal between the firing rate of the postsynaptic neuron and some target firing rate $R^{*}$. We mainly view this as a reward prediction error Montague et al. (1996); Schultz et al. (1997), as evidence suggests that the ventral striatum plays a major role in processing value estimates Daniel and Pollmann (2014); Pagnoni et al. (2002); Schultz et al. (1992). But this framework can also be applied more generally, as long as we assume that there is some optimal firing rate $R^{*}$ for whatever task the animal may be performing and that the error signal is proportional to the difference between $R^{*}$ and the actual firing rate. For simplicity we do not explicitly model the neural mechanisms that govern dopamine release, instead simply computing its value as follows:

(6) $$D=R^{*}-\bar{R}$$

where

(7) $$\bar{R}=\frac{1}{T_{\text{win}}}\int_{t_{\text{dop}}-T_{\text{win}}-T_{\text{del}}}^{t_{\text{dop}}-T_{\text{del}}}\rho^{\text{post}}(t)\;dt$$

is an estimate of the current firing rate. Here $T_{\text{win}}$ is the length of the time window over which the spike train is averaged (e.g., to produce a value estimate) and $T_{\text{del}}$ is a delay term between when the output firing rate is measured and when the dopamine is actually released (Figure 2a). This delay could be due to biological constraints, such as the speed of neural signal propagation or motor response, or to experimentally imposed delays; it has a significant impact on our analysis, as will be discussed below. For this model, we would like a plasticity mechanism that can learn to match the target firing rate $R^{*}$ on average, so that $R^{*}=E[\bar{R}]$.

The final model that we will consider is the *action selection setting*, as the basal ganglia including dorsal regions of striatum are hypothesized to play a critical role in action selection Kropotov and Etlinger (1999); Mink (1996). We implement this as a competition between two action channels Bogacz (2007); Bogacz and Gurney (2007); Mink (1996); Vich et al. (2022). Two neurons with weight vectors $w^{1}$ and $w^{2}$ (with entries $w_{i}^{j}$ for i∈{1,2,…,N} and j∈{1,2}) receive independent input spike trains generated from identical rate vectors r corresponding to shared presynaptic input sources. We compute estimates of their current firing rates ${\bar{R}}_{1}$, ${\bar{R}}_{2}$ as in equation (7), although unlike in the reward prediction setting we will usually set $T_{\text{del}}=0$ (see the discussion in Section 3.3). The animal then randomly chooses one of two actions, $A_{1}$ or $A_{2}$, using the output firing rates to determine the selection probabilities:

$$P_{j}=P\left(A=A_{j}\right)=\frac{e^{\beta {\bar{R}}_{j}}}{e^{\beta {\bar{R}}_{1}}+e^{\beta {\bar{R}}_{2}}}$$

for j∈{1,2}, where β is an inverse temperature parameter. (For simplicity, we take β to be an arbitrary large number in simulations, so that actions are chosen deterministically based on which channel has more spikes, with ties broken randomly.) The animal receives a reward $R^{*}$ depending on which action is taken: $R_{1}^{*}$ if $A_{1}$ is chosen, $R_{2}^{*}$ if $A_{2}$ is chosen. Finally, we compute the dopamine signal as the reward prediction error:

(8) $$D=R^{*}-E\left[R^{*}\right]=R^{*}-\left(R_{1}^{*}E\left[P_{1}\right]+R_{2}^{*}E\left[P_{2}\right]\right),$$

which is used to update the synaptic weights and hence $\rho^{\text{post}}(t)$ and $\bar{R}$, thus impacting future action selection. We would like a plasticity model that can learn to more frequently take the action that gives the higher reward. Note that like in the reward prediction setting, we do not model the neural mechanisms that may implement this process, instead simply computing $P_{1}$, $P_{2}$, and D explicitly.

In equation (8), $P_{1}$ and $P_{2}$ are now treated as random variables; we take the average value of $P_{1}$ and $P_{2}$ over instantiations of the spike trains with the given rates. That is, we sum over the possible postsynaptic spike counts in each channel:

$$E\left[P_{1}\right]=\sum_{i=0}^{\infty }\sum_{j=0}^{\infty }\frac{e^{i\beta /T_{\text{win}}}}{e^{i\beta /T_{\text{win}}}+e^{j\beta /T_{\text{win}}}}\frac{n_{1}^{i}e^{-n_{1}}}{i!}\frac{n_{2}^{j}e^{-n_{2}}}{j!}$$

where

$$n_{k}=T_{\text{win}}\frac{\langle w^{k},r\rangle }{N},\;k\in \{1,2\}$$

is the expected number of postsynaptic spikes in a window of length $T_{\text{win}}$; $E\left[P_{2}\right]$ is similar.

This definition assumes that the agent’s state-value function Sutton and Barto (2018) is accurate. In other words, the animal has learned the reward it receives on average when performing this task with its current policy (as defined by the weights $w^{1}$ and $w^{2}$). The idea that value estimates are available to neurons that drive action selection is commonly used in models and has ample experimental support (e.g., Samejima et al. (2005); Seo et al. (2012)). In practice these value estimates have to be learned, and as the animal’s policy changes, the value estimates will have to evolve along with it. We assume that the value estimates remain accurate (i.e., are learned instantaneously relative to the timescale of decision policy changes) as a simplification to allow us to focus on the action selection task without the added complication of a separate value learning circuit.

In the action selection setting, we silence all input to the striatal neuron between the end of one spike count window and the beginning of the next (i.e. for the duration of the delay if $T_{\text{del}}\neq 0$ as well as the period after dopamine is released). This step is designed to represent typical experimental settings in which the input stimulus does not persist after an action is taken in response to the stimulus. For instance, in a task in which a rodent must choose which branch of a maze to follow to receive a reward, the stimulus – the sight of the junction – necessarily cannot persist after the animal has made a choice and gone down one of the branches. However, we also consider a modification in which the cortex maintains some level of activity in the channel corresponding to the selected action Cisek and Kalaska (2005); Rubin et al. (2021) to help correctly assign credit for rewards to actions when they are separated by significant delays. We discuss this modification in more detail in Section 3.3. Figure 2 shows an illustration of the two versions of the action selection model as well as a comparison to the reward prediction model.

We also consider two variations on these basic scenarios. The first is *reward contingency switching* Vich et al. (2020); Bond et al. (2021), a variation of the action selection setting in which the mapping between actions and rewards is swapped periodically. The plasticity model should be able to update the learned weights based on the new reward schedule and switch which action it takes. The second is *task switching*, in which not only the rewards but also the input firing rate vector r switches between two (or more) possible values. Task switching can be applied to both the reward prediction setting and the action selection setting. In contrast to contingency switching, in which the neuron must switch which action it selects, in the task switching setting the neuron would ideally learn to perform *both* tasks using the same set of weights. (Of course, this is only possible in non-degenerate cases if the input dimension N is at least equal to the number of tasks to be learned.) This variant models the fact that a neuron will generally not be restricted to performing a single task, but rather may be active in a variety of different contexts.

One important simplification that we make in all settings is that the timing of dopamine release is independent of the spiking activity, and is simply treated as coming at some random time with mean rate $r^{\text{dop}}$. We also assume that dopamine releases are far enough apart that the dopamine level decays approximately to zero between them; in simulations, we implement this by simply using a fixed time interval between dopamine releases. These conventions contrast with models like the one described in Vich et al. (2020), which count the number of output spikes in a moving window and take an action (and subsequently release dopamine) as soon as the number crosses some threshold, and with models in which the CBGT circuit performs a process of evidence accumulation up to some threshold to make a decision Bogacz et al. (2006); Bogacz and Larsen (2011); Dunovan and Verstynen (2016); Dunovan et al. (2019); Vich et al. (2022). We opted to use a simpler mechanism here for analytical tractability. Although this may at first seem like a major simplification, in reality, if the neurons’ inputs in our tasks are statistically similar throughout the decision or reward estimation process as spikes are accumulated, then the output spiking characteristics preceding dopamine release on average are not related to the actual timing of dopamine release, only to its magnitude.

### 2.3 Simulations

We use the parameters listed in Table 2 as the defaults in our simulations for each of the three main task settings; any other parameters or changes to the defaults are listed in figure captions. “Steps” refers to the number of dopamine signals in an experiment; the number of steps used as well as λ were chosen to balance noise level with computation time and to illustrate phenomena of interest. $w_{\text{init}}=0.5$ was chosen arbitrarily; in some plots we instead use $w_{\text{init}}=0.33$ to illustrate time dynamics of weights because 0.5 would be too close to values that weights converge to. We chose input firing rates r to roughly match the frequency of cortical input to the striatum. As the random dopamine setting is meant to model neurons receiving spurious inputs, we use a lower input firing rate there. $R^{*},\;R_{1}^{*}$, and $R_{2}^{*}$ are arbitrary and were chosen for illustrative purposes. We use α=1 as the default scaling parameter in our weight update equations (equations (4) and (5)) for simplicity. For the choice of τ=0.02 s in equation (2), see Bi and Poo (1998); Gütig et al. (2003); note that Bi and Poo (2001) give values of τ=0.0168 s for long-term potentiation (LTP) and τ=0.0337 s for long-term depression (LTD), but as we do not distinguish between LTP and LTD in our model, we use the intermediate value of τ=0.02 s used in other sources. The half-life of dopamine has been estimated as 0.72 s in the dorsolateral striatum Riley et al. (2024); translating the half-life into an exponential time constant we get $\tau_{\text{dop}}\approx 1$ s. The choice of eligibility time constant $\tau_{\text{eli}}=1$ s reflects experimentally derived estimates Fisher et al. (2017); Yagishita et al. (2014) (but see Shindou et al. (2019), which finds a somewhat larger value). In the reward prediction setting, the delay time for dopamine release, $T_{\text{del}}$, was chosen to be long enough that any spikes occurring before the delay have minimal impact on the weight changes. In the action selection setting we generally use $T_{\text{del}}=0$. $r^{\text{dop}}$ was likewise chosen to be small enough that the effects of any interactions between adjoining dopamine signals would be negligible. The reward prediction and action settings use a longer period between dopamine signals than that used in the random dopamine setting to allow for the window $T_{\text{win}}$. The constant $\beta =10^{5}$ in the probability calculations is an arbitrary choice; other sufficiently large values would give similar results.

All figures use a sample size of 1000 for numerical results; error bars and bands show standard deviations. In some phase portraits we include fixed points; these were found analytically when possible, otherwise they were computed using the scipy.optimize library Virtanen et al. (2020). Note that some of the fixed points found on the boundaries are not “true” fixed points in the sense that they are not zeros of the dynamical equations. Rather, they are the result of the weights being clipped at 0 and 1. All code needed to run the simulations and reproduce the figures in the paper is available on GitHub at https://github.com/bsosis/DA-STDP.

## 3 Results

### 3.1 Random Dopamine Setting

When the dopamine signal is independent of spiking activity and has mean zero, the additive and multiplicative models in theory should exhibit zero net weight drift. This result arises because the dopamine is independent of the other terms in the weight update equation (4), so when taking the average weight drift we can factor out the average dopamine level, which is zero. This is not the case, however, for the corticostriatal model; here, the form of the weight update equation (5) depends on the sign of the dopamine signal, so the terms are not independent. It can be shown (see Section D) that on average the weights for the corticostriatal model converge to 1/(α+1).

**Fig. 3:**
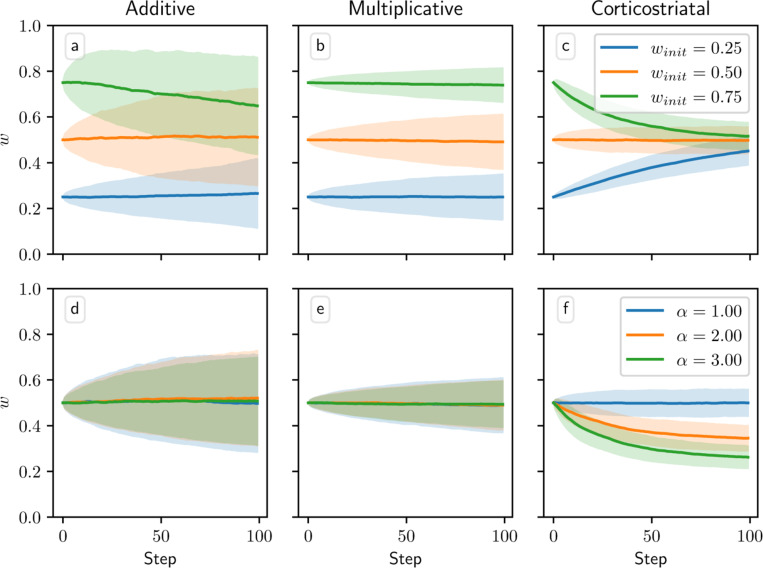
Weight evolution over time in the random dopamine setting. Columns show the additive (a, d), multiplicative (b, e), and corticostriatal (c, f) models. (a-c) the initial weight $w_{\text{init}}$ is varied while α=1 is fixed. (d-f) α is varied while $w_{\text{init}}=0.5$ is fixed

These outcomes are illustrated in Figure 3. In practice, the weights for the additive and multiplicative models do show some fluctuations about their means, which grow over time, as well as some boundary effects where clipping the weights to 0 and 1 pushes the mean weight values away from the boundaries. This is most visible for the additive model. In this case, the weight drift is proportional to w, so the upper curve will experience larger fluctuations than the lower curve; moreover, since weight increases are being truncated, there is a bias that causes a net downward drift. However, motion away from the initial conditions for both models is generally fairly slow. In contrast, the mean weight values for the corticostriatal model quickly converge to 1/(α+1). Thus, under the corticostriatal model without any supplementary weight maintenance mechanism, any noise will tend to erase previously learned weights.

### 3.2 Reward Prediction Setting

Under suitable assumptions it is possible to derive a formula for the average weight drift over time for the additive and multiplicative models under the reward prediction framework:

(9) $${\dot{w}}_{i}=\left(R^{*}-\frac{1}{N}\langle w,r\rangle \right)r^{\text{dop}}\tau_{\text{dop}}\tau_{\text{eli}}\frac{\lambda }{N}\left(\tau \text{Δ}f\left(w_{i}\right)r_{i}\langle w,r\rangle +f_{+}\left(w_{i}\right)w_{i}r_{i}\right)$$

where $\text{Δ}f=f_{+}-f_{-}.$ (See Section A.1 for the derivation. An important assumption here is that the delay $T_{\text{del}}$ is large relative to $\tau_{\text{eli}}$; this assumption will be discussed below.) The terms in this expression have a simple interpretation: $\tau \text{Δ}f\left(w_{i}\right)r_{i}\langle w,r\rangle$ corresponds to independent pairs of pre- and postsynaptic spikes (both pre-before-post and post-before-pre), $f_{+}\left(w_{i}\right)w_{i}r_{i}$ corresponds to a pre-post spike pair in which the presynaptic spike directly causes the postsynaptic neuron to fire, and $R^{*}-\frac{1}{N}\langle w,r\rangle$ is the average dopamine level, which is the difference between the target firing rate and the mean output firing rate.

The average weight drift equation (9) is fairly easy to analyze. Its most important feature is what we will call the *solution plane*: the hyperplane of weight values such that $\frac{1}{N}\langle w,r\rangle =R^{*}$. These are weights such that the output firing rate equals the target firing rate $R^{*}$ and hence they are solutions to the task the neuron has to learn. It is clear from equation (9) that any point on this plane is a fixed point, which corresponds to the average dopamine signal being zero. However, the solution plane is not necessarily stable. We give a sufficient condition for the existence of a stable solution (that is, a stable fixed point on the solution plane) in the following theorem.

#### Theorem 1.

*Pick* $r\in {\mathbb{R}}^{N}$ *and* $R^{*}\leq \frac{1}{N}∥r{∥}_{1}$, *and let* $w^{\prime }=NR^{*}/∥r{∥}_{1}$. *If*

(10) $$f_{-}\left(w^{\prime }\right)<\left(1+\frac{1}{\tau ∥r{∥}_{1}}\right)f_{+}\left(w^{\prime }\right),$$

*then there exists a stable point on the solution plane, given by* $w=\left(w^{\prime },\ldots ,w^{\prime }\right)$.

*Proof.* See Section B.2. □

For the additive model, condition (10) can be rewritten as

$$\tau (\alpha -1)<\frac{1}{∥r{∥}_{1}}$$

whereas for the multiplicative model, it can be written as

$$R^{*}<\frac{w_{0}}{N}∥r{∥}_{1}$$

where

$$w_{0}=\frac{\tau ∥r{∥}_{1}+1}{\tau (1+\alpha )∥r{∥}_{1}+1}.$$

See Section B for derivations and more details on the stability of the solution plane.

The additive model in general has no other equilibria besides the solution plane (and points on the boundary). However, when $\tau (\alpha -1)=1/∥r{∥}_{1}$, points on the line $w_{i}=w_{j}$ for all i≠j are also equilibria. Points on the line are stable when $R^{*}-\frac{1}{N}\langle w,r\rangle <0$; that is, the line is stable on one side of the solution plane. This statement can be proven using a similar approach to that used in Section C.1.

The multiplicative model has an extra fixed point at $w=\left(w_{0},\ldots ,w_{0}\right)$. We can characterize its stability as follows:

#### Theorem 2.

*For the multiplicative model, if*

$$R^{*}<\frac{w_{0}}{N}∥r{∥}_{1}$$

*then the Jacobian at the fixed point* $w=\left(w_{0},\ldots ,w_{0}\right)$ *is positive definite (and so the point is unstable); if*

$$R^{*}>\frac{w_{0}}{N}∥r{∥}_{1}$$

*then the Jacobian is negative definite (and so the point is stable)*.

*Proof.* See Section C.2. □

The corticostriatal model, however, is more difficult to analyze. Because the form of the plasticity rule depends on the sign of the dopamine signal, in general it is not possible to factor out the average dopamine level $R^{*}-\frac{1}{N}\langle w,r\rangle$ like we can for the additive and multiplicative models. We analyze this model further in Section A.2; in general, points on the solution plane will not be fixed points under this model.

**Fig. 4:**
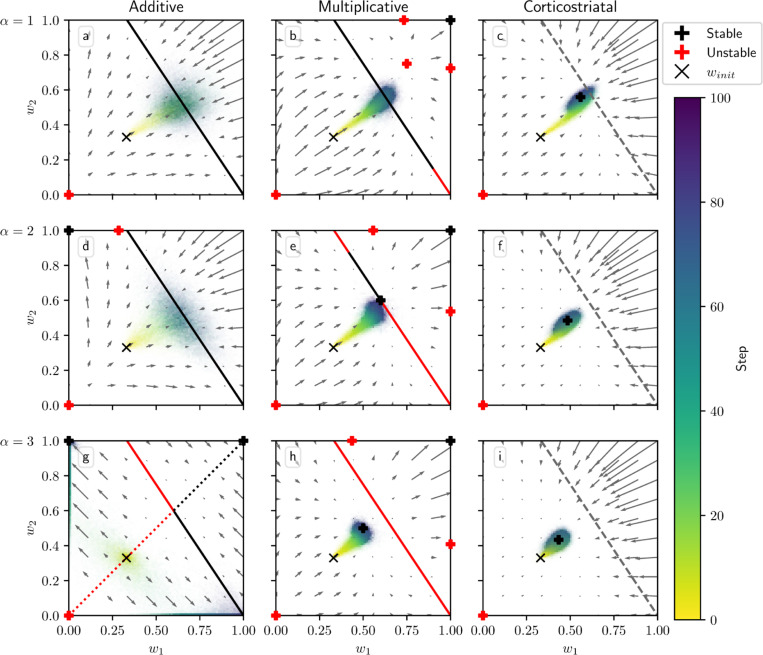
Distribution of weights over time in the reward prediction setting as α is varied. The color code indicated in the color bar shows the simulation step. Columns show the additive (a, d, g), multiplicative (b, e, h), and corticostriatal (c, f, i) models. α is varied across rows: (a-c) α=1; (d-f) α=2; (g-i) α=3. Each panel includes arrows showing the vector field of the averaged model as well as the solution plane, which is the negatively sloped line. For the corticostriatal model, the solution plane is dashed because it does not govern the dynamics for this model. Red coloring of the solution plane and points off of the plane indicates unstable fixed points; black indicates stable. We use $w_{\text{init}}=0.33$ here, marked by the “×” in each plot. (g) has an extra line of equilibria where $w_{1}=w_{2}$ (dotted). Note that in (g) most of the sample paths end up being driven to the upper left and lower right corners

Figure 4 shows phase portraits for the averaged models for three different values of α. To generate each plot, we ran a set of simulations of the fully stochastic implementation (see Section 2.3) of the appropriate model with N=2 weights and initial conditions $\left(w_{1},w_{2}\right)=0.33$ as marked by the × symbol. In each simulation, from this starting point, $w_{1}$ and $w_{2}$ evolved over 100 time steps, and the position of $\left(w_{1},w_{2}\right)$ at certain time steps was plotted as a point of the time-dependent color indicated by the color bar; this process resulted in a cloud of points over many simulations, representing the distribution of weights. Each plot also includes the solution plane (here, a line), any relevant fixed points, and vector field arrows for the averaged model. The orientations of these arrows indicate the directions that trajectories would move over time under the flow of the averaged model, while their lengths represent the magnitudes of the weights’ rates of change.

We see that as α increases, a larger fraction of the solution plane becomes unstable for the additive and multiplicative models. Figure 4g includes the additive model’s extra line of fixed points that exists for certain parameter values, as mentioned above. For the multiplicative model, the isolated extra fixed point crosses the solution plane and exchanges stability with it (Figure 4b, e, h). The solution plane does not consist of equilibria for the corticostriatal model; as can be seen, it does not play a role in shaping the model’s dynamics like it does for the additive and multiplicative models. In general, the averaged models capture the dynamics well, as can be seen from the dispersal patterns of trajectories in relation to the averaged model vector field and their convergence to stable fixed points, although depending on the model and the choice of parameters there can be substantial variability across these trajectories.

These results show that while the additive and multiplicative models can perform reward prediction tasks under suitable choices of the parameters, the corticostriatal model cannot. In the latter case, weights in general do not converge to the solution plane, so the postsynaptic neuron’s output firing rate will not match $R^{*}$ except by coincidence. In contrast, with the appropriate parameters, most or all of the solution plane may be stable for the additive and multiplicative models. The additive model in fact performs best here, because it does not have the extra fixed point of the multiplicative model; even when the solution plane is stable, the unstable extra fixed point can drive trajectories from some initial conditions away from the solution plane towards the boundaries, as occurs for initial conditions with large enough $w_{1},w_{2}$ in Figure 4b. Meanwhile, most trajectories under the additive model appear to converge to the solution plane, although for large enough α the convergent proportion drops as more of the plane becomes unstable. Our mathematical results serve to characterize the ranges of parameter values where stable points on the solution plane exist. Specifically, they highlight the important role that α and τ play in the dynamics: increasing either parameter reduces the range of r and $R^{*}$ values that support stable solutions.

Next, we consider task switching in which the rewards and input firing rates switch between two values. In this scenario, the average model dynamics can be quite complex, as there are two solution planes, one per set of rewards and input rates, each of which may have stable and unstable regions. Moreover, depending on the length of time between task switches, the weights may either bounce between the fixed points for the different tasks (if they do not coincide) or approximately follow the average of the weight drift equations of the tasks. If the solution planes intersect, then we would like the weights to converge to their intersection, so that the neuron can accomplish both tasks. If they do not intersect, then ideally the weights should converge to some point close to both of them, which would constitute an approximate solution to both tasks.

**Fig. 5:**
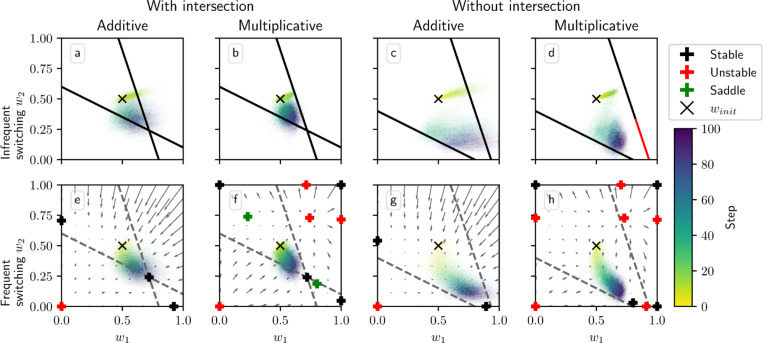
Distribution of weights over time in the reward prediction setting with task switching. First and third columns (a, c, e, g) show the additive model; second and fourth columns (b, d, f, h) show the multiplicative model. For (a, b, e, f) the task switches between $r=(15,5)\;{\text{s}}^{-1}$, $R^{*}=6$ and $r=(10,20)\;{\text{s}}^{-1}$, $R^{*}=6$, which yields a solution plane intersection at w=(0.72,0.24). For (c, d, g, h) the task switches between $r=(15,5)\;{\text{s}}^{-1}$, $R^{*}=7$ and $r=(10,20)\;{\text{s}}^{-1}$, $R^{*}=4$, which does not give a solution plane intersection in [0,1]^2^. In the upper row (a-d) switching is infrequent (every 20 steps out of 100), while in the lower row (e-h) switching is frequent (every step). For the infrequent switching plots we include the solution planes to demonstrate their relation to where the trajectories converge, but because switching between the two forms of dynamics is infrequent, we cannot display a meaningful vector field illustration. For the frequent switching plots we include the vector field for the dynamics computed by averaging the dynamical equations for the two tasks; as switching is frequent, averaging approximately captures the behavior of the system. We also plot the solution planes here for illustrative purposes, but they are dashed because they do not control the trajectories. Fixed points are estimated numerically: red indicates unstable fixed points; black indicates stable; green indicates saddle points. The “×” indicates the initial point, in this case $\left(w_{1},w_{2}\right)=(0.5,0.5)$

Figure 5 shows the densities of trajectories of the additive and multiplicative models performing task switching in two example settings: one in which the solution planes intersect and one in which they do not. The upper row uses a long interval between switches, while the lower row switches after each step. (We do not include the corticostriatal model since, as discussed above, it cannot accomplish reward prediction tasks.) As can be seen, if the solution planes intersect, then (for suitable initial conditions) much of the density ends up concentrated at their intersection. If the planes do not intersect then the weights are generally driven to regions close to both planes, although results are less ideal in the multiplicative model due to complications such as an unstable solution plane segment (Figure 5d), an off-plane stable fixed point (Figure 5h), and regions of initial conditions that are impacted by an unstable fixed point (Figure 5h, upper right corner). Overall, while the precise details depend strongly on the choice of inputs and other parameters, the additive and multiplicative models do generally seem able to perform well at reward prediction in a task switching settings.

**Fig. 6:**
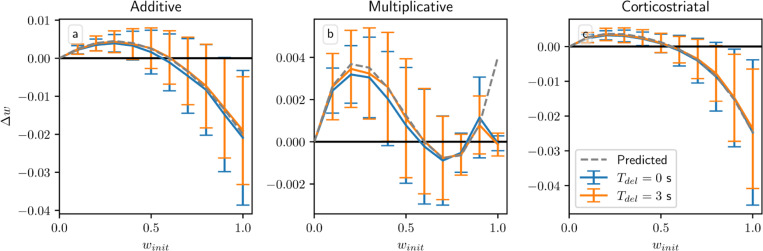
Weight drift after a single dopamine release in the reward prediction setting with variable $T_{\text{del}}$. Plots show results for the additive (a), multiplicative (b), and corticostriatal (c) models, with $T_{\text{del}}=0$ s and $T_{\text{del}}=3$ s, as well as the predicted weight drift based on the averaged models, as $w_{\text{init}}$ is varied for N=1. Here $r=10\;{\text{s}}^{-1}$ and $R^{*}=6$; we also use λ=0.0005. Note that when $w_{\text{init}}=1$ there are some deviations from predictions even for $T_{\text{del}}=3$ s due to boundary effects not taken into account by the averaged models

All of these results, however, depend on a key assumption: that the delay $T_{\text{del}}$ is long relative to $\tau_{\text{eli}}$. By imposing a large gap between when the firing rate is measured, using equation (7), and when dopamine is actually released, the delay ensures that the dopamine signal is statistically independent of the other terms in the weight update equation. This is what allows us to factor out the dopamine term $R^{*}-\frac{1}{N}\langle w,r\rangle$ in the average drift formula, equation (9). Without this term, we can no longer guarantee that points on the solution plane $R^{*}-\frac{1}{N}\langle w,r\rangle$ are equilibria for any of the three plasticity models. In Figure 6 we plot the change in weight after a single dopamine release as a function of $w_{\text{init}}$ in an N=1 setting. When $T_{\text{del}}=3$ s the simulations obey the predictions of the averaged models, and in the additive and multiplicative cases they intersect the x-axis at w=0.6, the point at which $R^{*}=wr$ for these parameters. (While the plots may appear fairly noisy, keep in mind that they only display the change in weight after a single dopamine signal. Figure 4 shows that although there is some dispersion, over the course of many trials trajectories still tend to follow the averaged dynamics.) When $T_{\text{del}}=0$ s the simulations do not exactly match the predictions, but the differences are fairly small. In most cases the $T_{\text{del}}=0$ s curves are below the curves for $T_{\text{del}}=3$ s. This undershoot may occur because there is a source of negative correlation between the dopamine value and the eligibility at the time that dopamine is released. Specifically, the dopamine value D from equation (6) is negatively correlated with the number of postsynaptic spikes in the spike count window, while the eligibility will in most cases (depending on the plasticity model and the parameters) be positively correlated with the number of recent spikes. If there is no delay, then this will include the spikes in the spike count window used to compute the dopamine value. While these plots show that for realistic parameter values our model is not very sensitive to the delay or its absence, it should be noted that for other sets of parameters, for instance smaller values of $\tau_{\text{dop}}$, $\tau_{\text{eli}}$, and $T_{\text{win}}$, the lack of a delay can have a significant effect.

**Fig. 7:**
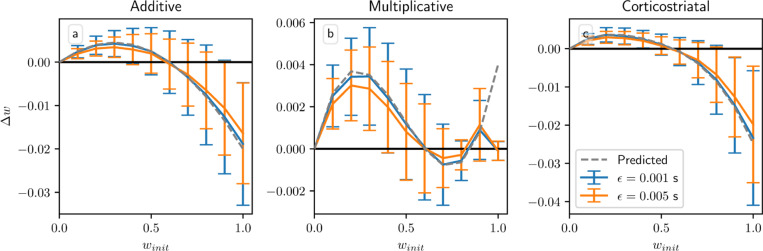
Weight drift after a single dopamine release in the reward prediction setting with variable ϵ. Plots show results for the additive (a), multiplicative (b), and corticostriatal (c) models, with ϵ=0.001 s and ϵ=0.005 s, as well as the predicted weight drift based on the averaged models, as $w_{\text{init}}$ is varied for N=1. Here $r=10\;{\text{s}}^{-1}$ and $R^{*}=6$; we also use λ=0.0005. Note that when $w_{\text{init}}=1$ there are some deviations from predictions even for ϵ=0.001 s due to boundary effects not taken into account by the averaged models

Another assumption in our analysis is that ϵ, the time between presynaptic spikes and any postsynaptic spikes they cause, is small relative to τ, the time constant of synaptic plasticity. Specifically, we assume following Gütig et al. (2003) that $e^{-\epsilon /\tau }\approx 1$; using our default values of ϵ=0.001 s and τ=0.02 s, this quantity is $e^{-\epsilon /\tau }=0.95$. In Figure 7 we show the result of increasing ϵ to 0.005 s, in which case $e^{-\epsilon /\tau }=0.78$. The main effect of increasing ϵ is to reduce the magnitude of the changes in weight. τ defines the duration of the window of synaptic plasticity; as ϵ increases, pre- and postsynaptic spikes grow farther apart relative to τ, and so weight changes due to presynaptic spikes directly causing postsynaptic spikes (corresponding to the $f_{+}\left(w_{i}\right)w_{i}r_{i}$ term in equation (9) for the additive and multiplicative models; the other terms correspond to spike pairs that are close together only by chance) are reduced by a factor of $e^{-\epsilon /\tau }$. Overall, though, for realistic values of ϵ the differences between the two curves are small, and the qualitative behavior is largely unchanged.

### 3.3 Action Selection Setting

**Fig. 8:**
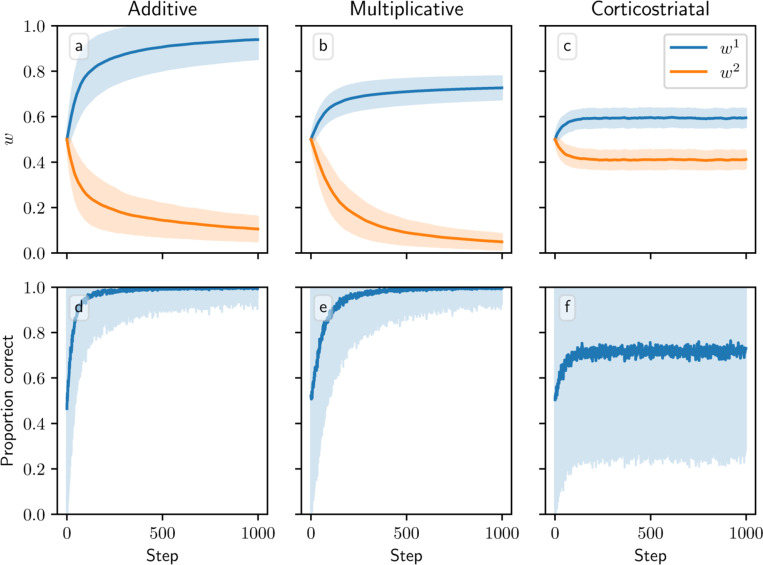
Model performance in the action selection setting. Plots show weights (a-c) and probability of taking the correct action (d-f) versus time for the additive (a, d), multiplicative (b, e), and corticostriatal (c, f) models. Shaded envelopes show standard deviations while solid lines show means over 1000 trials

We next consider a task of selecting between two actions, in which action 1 gives a higher reward than action 2. (We do not have expressions for the averaged dynamics on this task, so our results in this section will rely on simulations.) In this setting, all three models successfully learn to take action 1 more often than action 2, but major differences arise among the values to which the weights converge across the three models (Figure 8). The additive model drives $w^{1}$ towards one and $w^{2}$ towards zero. The multiplicative model likewise drives $w^{2}$ towards zero, but $w^{1}$ only reaches around 0.73±0.05 after 1000 steps. Meanwhile under the corticostriatal model, $w^{1}$ and $w^{2}$ converge to limits of around 0.56 ± 0.04 and 0.41 ± 0.04, respectively. (These values depend on the particular parameters chosen.) All three models can therefore accomplish this task, although the additive and multiplicative models choose the correct action more consistently than the corticostriatal model does (Figure 8).

**Fig. 9:**
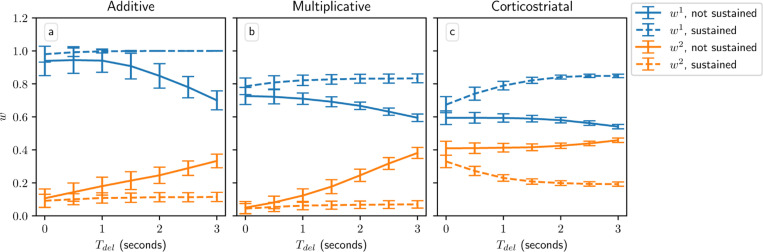
Performance in the action selection setting as delay is varied with and without sustained activity. Plots show weights after 1000 steps for additive (a), multiplicative (b), and corticostriatal (c) models. With no sustained activity, both input channels are silenced during the delay period, while with sustained activity, the input to the selected channel is maintained at a level of 70% (see Figure 2)

The delay plays an important role in this model, too. Figure 9 shows the weight distributions after 1000 steps for the three models as a function of $T_{\text{del}}$; with too long of a delay, the models are unable to learn (i.e., the difference between $w_{1}$ and $w_{2}$ becomes too small) because the dopamine signal becomes uncorrelated with eligibility at the time dopamine is released. This is an instance of the *credit assignment problem* Houk et al. (1994). Rubin et al. (2021) propose that the brain solves this problem via sustained cortical activity in the selected action channel and reduced activity in the unselected channel, building off of experimental results showing this pattern of activity Cisek and Kalaska (2005). The corresponding sustained corticostriatal input ensures that while the spikes that directly caused an action to be selected do not themselves contribute to the weight changes, there will still be a correlation between the dopamine signal and the spiking activity at the time dopamine is released due to the differences in firing rates (see Figure 2). As can be seen in Figure 9, with sustained activity in the selected channel the models are able to successfully produce large differences between $w_{1}$ and $w_{2}$, and hence learn the task, even when $T_{\text{del}}$ is large.

**Fig. 10:**
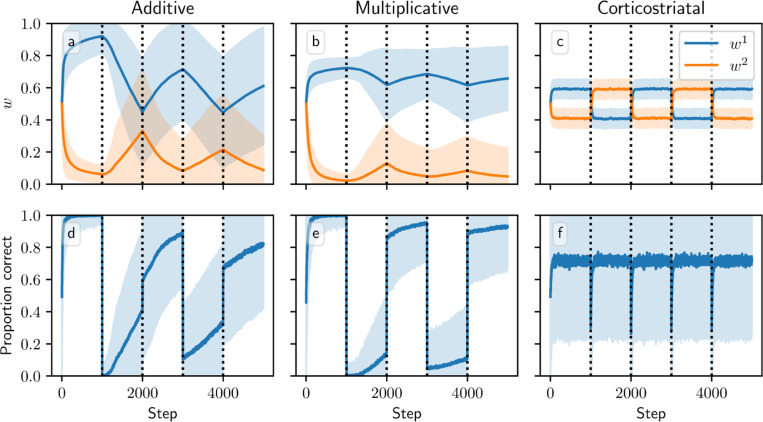
Model performance in the action selection setting with contingency switching. Plots show weights (a-c) and probability of taking the correct action (d-f) versus time for the additive (a, d), multiplicative (b, e), and corticostriatal (c, f) models. Here $r=10\;{\text{s}}^{-1}$ and the reward contingencies switch between $R_{1}^{*}=2$, $R_{2}^{*}=1$ and $R_{2}^{*}=1$, $R_{2}^{*}=2$ every 1000 steps; we use λ=0.05 for illustrative purposes

Figure 8 and Figure 9 show results for learning of a single relation between action and reward. In some situations, both in experiments and in natural settings, relations between actions and subsequent rewards can change over time, an effect that we refer to as contingency switching. To simulate these tasks, we swap which action is mapped to the higher reward value every 1000 steps. In this situation, we find that substantial differences arise in performance among the three models. Figure 10 shows that the additive and multiplicative models are unable to perform these tasks well, because the weights get stuck near the widely spread values that they attain for the first contingency scenario. Running the simulations with longer intervals between switches would not help as the weights take just as much time to escape from these values as they spend approaching them; that is, longer intervals lead to stronger convergence and hence more time needed to move away after a contingency switch. The corticostriatal model, in contrast, is able to quickly react to the contingency switches and swap which action it takes, resulting in only brief drops in accuracy when switches occur.

**Fig. 11:**
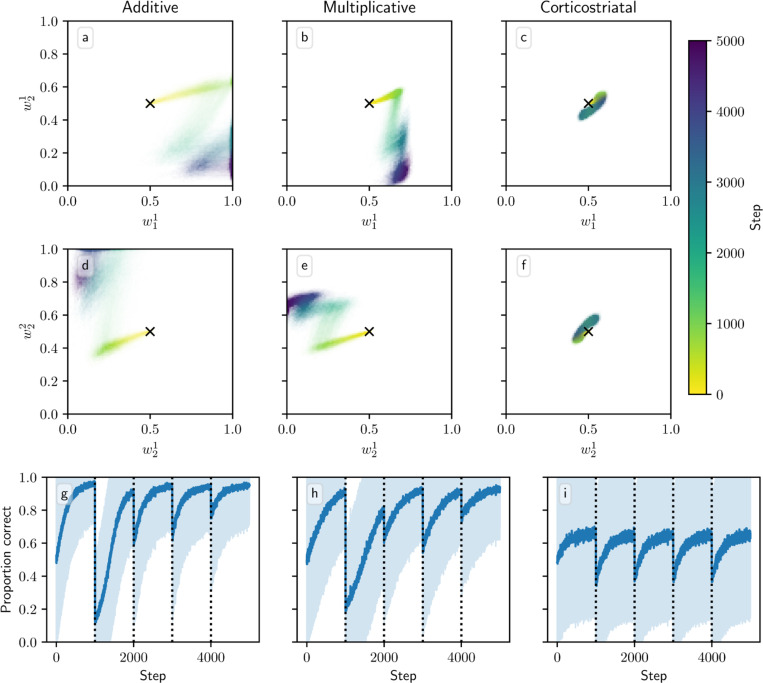
Model performance in the action selection setting with infrequent task switching. Plots show weights (a-f) and probability of taking the correct action (g-i) versus time for the additive (a, d, g), multiplicative (b, e, h), and corticostriatal (c, f, i) models. The input switches every 1000 steps between $r=(15,5)\;{\text{s}}^{-1}$, $R_{1}^{*}=2,\;R_{2}^{*}=1$ and $r=(5,15)\;{\text{s}}^{-1}$, $R_{1}^{*}=1,\;R_{2}^{*}=2$. Channel 1 is displayed in (a-c) and channel 2 in (d-f); each one consists of two weights. The optimal weight vectors are $w^{1}=(1,0)$ and $w^{2}=(0,1)$, which would, by design, allow the model to preferentially choose action 1 in state 1 and action 2 in state 2. In these plots λ=0.05

**Fig. 12:**
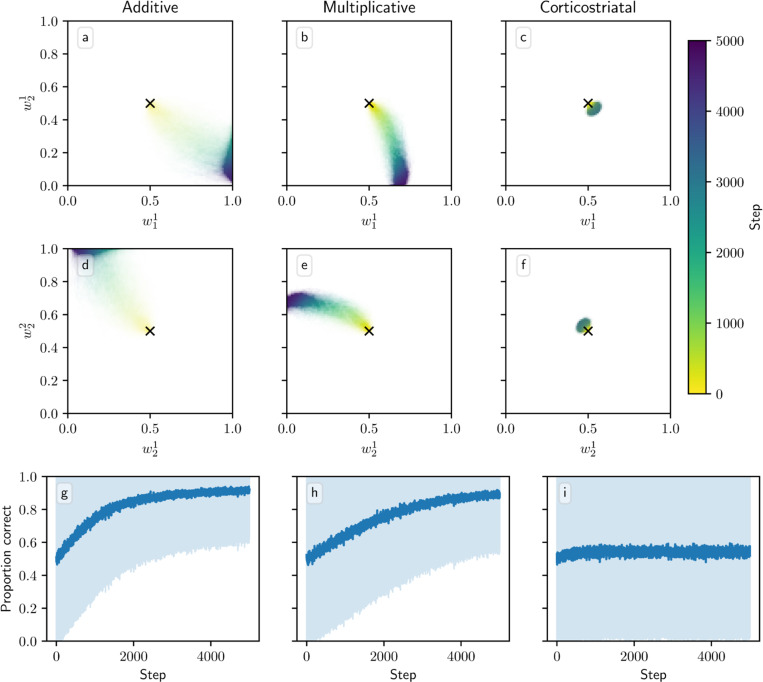
Model performance in the action selection setting with frequent task switching. Plots show weights (a-f) and probability of taking the correct action (g-i) versus time for the additive (a, d, g), multiplicative (b, e, h), and corticostriatal (c, f, i) models. Parameters are the same as in Figure 11 except that task switching occurs at every simulation step

We also tested model performance in task switching in the action selection task. Figure 11 shows model trajectories and proportion of trials on which the more rewarding action is chosen under infrequent switching, where the inputs and rewards are swapped every 1000 steps. All three models are able to switch which action they take when the state switches. But whereas the additive and multiplicative models are able to learn a set of weights that can yield high probabilities of selection of the more rewarded action in both states, the corticostriatal model struggles to do so because of the more limited range of values the weights take under its dynamics. The corticostriatal model is able to recover its prior performance after a task switch, but it does not seem to learn one set of weights that have above-chance performance on both tasks. When switching is frequent (every step), the corticostriatal model learns weights that give performance only slightly better than chance, while the additive and multiplicative models successfully learn weights that perform well in both states (see Figure 12). (Note that in both Figures 11 and 12, the weights under the corticostriatal model stay near the initial value of $w_{\text{init}}=0.5$ because of the presence of equilibria nearby; had we used different initial conditions the weights would still quickly converge to the values shown in the figures.)

### 3.4 Symmetric Model

None of the models we have considered can accomplish every task we set for them. Can we use our findings to design a plasticity model that can? Here we consider one possibility. Rather than switching whether we scale weight changes by w or 1−w depending on pre-post spike timing, as we do in the multiplicative and corticostriatal models, we simply use w(1−w) irrespective of the direction of the weight update; in other words, rather than equations (4) or (5) we use

$$\frac{dw_{i}}{dt}=\lambda D(t)w_{i}(t)\left(1-w_{i}(t)\right)\left(E_{i}^{+}(t)-E_{i}^{-}(t)\right).$$

We will refer to this model as the *symmetric model*. This model fixes the issue with the multiplicative model where w may be used when weights are increasing and 1−w used when weights are decreasing, which may occur if the dopamine signal is negative. Moreover, the dopamine signal can be factored out of the update equation for the symmetric model, unlike with the corticostriatal model; it is therefore to be expected that the symmetric model will perform well in the random dopamine and reward prediction settings where the corticostriatal model does poorly.

**Fig. 13:**
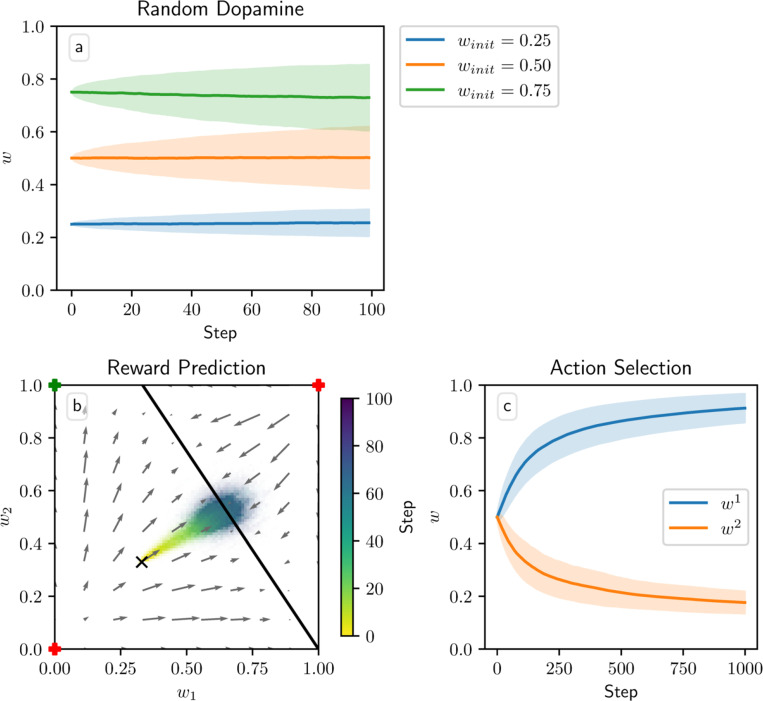
Weight evolution in the three main settings for the symmetric model. This model requires the use of relatively large values of λ: (a) in the random dopamine setting, λ=0.02; (b) in the reward prediction setting, λ=0.0066; (c) in the action selection setting, λ=0.05 (double their default values)

**Fig. 14:**
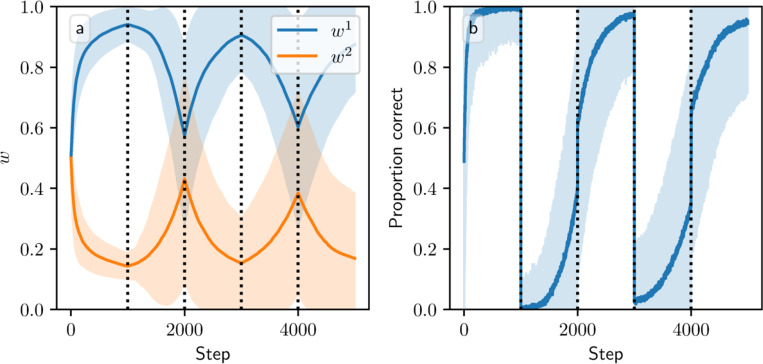
Performance of the symmetric model in the action selection setting with contingency switching. Plots show weights (a) and probability of taking the correct action (b) versus time. Here λ=0.1, and the other parameters are the same as those used in Figure 10

Experimentally, we see that the symmetric model maintains the good performance of the additive and multiplicative models in the random dopamine and reward prediction settings as well as the basic action selection setting (Figure 13). However, it does not do as well as the corticostriatal model in the action selection setting with infrequent contingency switching. In this setting the weights for the corticostriatal model converge to fixed points some distance from the boundaries and contingency switching seems to swap the locations of the stable fixed points, allowing the model to respond quickly to switches (see Figure 10c). Under the symmetric model, weights still get driven towards the boundaries. While they go to the boundaries much more slowly than under the additive and multiplicative models due to the w(1−w) term, they also take correspondingly longer to leave once they get there (Figure 14). So while the symmetric model may be an improvement over the additive and multiplicative models in some ways, it does not seem to provide a panacea for the other models’ shortcomings.

## 4 Discussion

Accurately modeling learning in the cortico-basal ganglia-thalamic circuit requires the use of an appropriate synaptic weight update rule for the dopamine-dependent STDP in the corticostriatal connections. In this paper we examine three plasticity models that combine dopamine, eligibility, and spike timing signals in different ways – the additive, multiplicative, and corticostriatal models – and evaluate their performance in a number of different task settings. We find that the additive and multiplicative models do well in many cases: they are able to maintain weights in the presence of random inputs and dopamine release events, they can learn to predict a reward, and they can accomplish a simple action selection task. They do not perform well on action selection tasks in which the reward contingencies occasionally switch, however, because they tend to get stuck at or near the boundaries of the weight domain. In contrast, the corticostriatal model, while performing poorly in the random dopamine and reward prediction settings, is able to rapidly relearn swapped reward contingencies in the action selection setting. This rapid learning matches the results seen in experiments with animals Beron et al. (2022) and humans Bond et al. (2021). When tasks instead of contingencies switch, however, the success of the corticostriatal model is hindered somewhat by the restricted range of synaptic weight values that it induces. Overall, we find that the choice of which plasticity model to use can have a large impact on the dynamics of synaptic weights and hence on both the learning achieved by the circuit and the ability of the model to perform a given task. Which plasticity model is appropriate depends strongly on the tasks it will be asked to perform. Ultimately, these results suggest that different synaptic plasticity mechanisms may be at play at corticostriatal synapses involving different regions of the striatum with distinct functions, as well as at corticocortical synapses with dopamine-dependent plasticity Otani et al. (2003), and that additional experimental and theoretical work is needed to pin down the precise forms of plasticity that occur at corticostriatal synapses and how they should be modeled.

Our mathematical analysis of the random dopamine and reward prediction settings shows how the choice of parameter values impacts model performance on these tasks. Specifically, we found that in the random dopamine setting that under the corticostriatal model, weights evolve to 1/(α+1); in the reward prediction setting under the additive and multiplicative models, we characterized how the existence of stable points on the solution plane (and therefore the ability of the model to solve the task) depends on the parameters α,τ,r, and $R^{*}$. In general, increasing α, the strength of negative eligibility relative to positive, and τ, the STDP time constant, will reduce the ranges of r and $R^{*}$ values that feature stable solutions. Therefore, these parameters are particularly important for practitioners using these models to understand and to select judiciously.

Why exactly do the three plasticity models run into difficulties in some settings? An important issue with the additive model is that it does not prevent weights from being driven to the boundaries (or past them, if the weights are not artificially cut off). The original multiplicative model without dopamine avoids this complication by scaling the weight drift by w if weights are decreasing and by 1−w if they are increasing. Our version of the model with dopaminergic modulation disrupts this property, though: because the w and 1−w terms are tied to the sign of the eligibility but not the sign of the dopamine signal, if the dopamine signal is negative, then the wrong term is applied (w for increasing weights and 1−w for decreasing weights). This effect can lead to weights being driven to zero in the action selection setting. The corticostriatal model solves this problem by selecting w or 1−w depending on the sign of the product of the dopamine signal term with the eligibility trace term. In other words, it ensures that even with dopamine the correct scaling term will be chosen: w for decreasing weights and 1−w for increasing weights (see Table 1). The cost of this modification, from an analytical perspective, is that the dopamine signal can no longer be factored out of the weight drift equation. Consequently the corticostriatal model features nonzero mean weight drift even when the mean dopamine signal is zero, leading to its failure to maintain pre-learned weights under random dopamine and its failure to converge to the solution plane in the reward prediction setting. Recent experimental results on local control of dopamine release within the striatum Cachope and Cheer (2014); Nolan et al. (2020); Holly et al. (2024) suggest that neurons may express more complicated mechanisms that we have not modeled that allow them to avoid spurious weight changes when not involved in task performance, which may ameliorate the difficulties of the corticostriatal model in the random dopamine setting. On the theoretical side, we introduced the symmetric model considered in Section 3.4 as an attempt to have the best of both worlds: a model that properly scales weight updates near the boundaries while allowing the dopamine signal to be factored out of the weight drift equation. Unfortunately, it does not significantly improve on the poor performance of the additive and multiplicative models in the action selection setting with contingency switching.

The corticostriatal model has another problem: its weights tend to remain in a relatively narrow band, leading to a fairly low probability of taking the correct action in the action selection setting. This probability is determined by the number of postsynaptic spikes in each channel, and if the weights are close together, then spiking noise will sometimes lead to more spikes being counted in the incorrect channel, causing the wrong action to be taken. This outcome occurs despite the fact that we use a large value of β, the temperature parameter in our action selection probability function. We believe that this problem is not a fundamental one, however, as it can be easily solved through downstream integration over the outputs of multiple striatal neurons to obtain a clearer signal.

One important issue that we have highlighted throughout this work is the impact that delays have on the weight dynamics. In the reward prediction setting, we need to ensure that there is a sufficiently long delay between when we estimate the postsynaptic firing rate and when dopamine is actually delivered; without this delay, we cannot guarantee convergence to the solution plane due to correlations between terms (although in practice this does not substantially affect our results). On the other hand, we need to use short delays in the action selection setting without sustained activity in the selected channel, because these correlations are required for the model to learn which action to take. A complicating factor that we have not addressed is that experimental results consistently show that dopamine release immediately upon pre-post spike pairing does *not* lead to a change in weight; rather, the dopamine must come some time after the spiking activity to effect significant synaptic changes Shindou et al. (2019); Yagishita et al. (2014). Moreover, dopamine is not released instantly, but rather takes some time to ramp up to its peak value Riley et al. (2024). These findings raise important questions about how to best understand and model delays within a synaptic plasticity framework. Although we considered both the dopamine concentration and the eligibility trace as jumping up immediately and then decaying exponentially, for the sake of analytical tractability and for consistency with prior computational work, an important extension of these results would be to represent them as slowly ramping up and then ramping down over time and to study how these more realistic time-courses interact with delays and the computational roles that they play.

Our additive and multiplicative models are based on the plasticity rules described in Gütig et al. (2003), but our plasticity rules differ from theirs in that we incorporate dopaminergic modulation of the synaptic plasticity. The random dopamine setting is closest to the one they use, and indeed by fixing the mean dopamine level to some positive constant (rather than drawing it from a normal distribution centered at zero) we can reproduce their setting very closely, the only difference being that our model only undergoes plasticity during the periodic dopamine signals rather than after every spike pair. Our goals are quite different from those of the earlier work, however: while they study conditions under which symmetry breaking in the weight distributions occurs and when the models can learn to represent correlations in a set of inputs, we instead use the random dopamine setting to investigate the stability of learned weights under perturbation.

The corticostriatal model in this paper is based on the plasticity model used in Clapp et al. (2024) but differs from their model in a number of important ways. We make several simplifications to the model, including setting the scaling factors and time constants for pre- and postsynaptic spike traces equal to each other (in their notation, $\tau_{PRE}=\tau_{POST}$ and ${\text{Δ}}_{PRE}={\text{Δ}}_{POST}=1$), as well as considering a single class of striatal neurons rather than taking into account the existence of multiple striatal neuron subpopulations with different plasticity properties. They also employ their plasticity model in a more biologically realistic setting, incorporating many components of the basal ganglia circuitry that we leave out. The most interesting difference between our models is that they use a single eligibility trace summing up both positive (corresponding to pre-before-post spike pairs) and negative (post-before-pre) contributions, while we use two different traces for the positive and negative components. The use of two traces is justified by experimental evidence suggesting that the brain uses two distinct eligibility traces for LTP and LTD He et al. (2015). (Note, however, that the computational model introduced in He et al. (2015) differs considerably from the models used here, as it does not use αw or 1−w factors to rescale the positive and negative traces, instead simply adding them together without modification.) We find in Section E that altering our models to employ a single eligibility trace leads to qualitatively similar results in most cases, although they are much more difficult to analyze.

A number of other three-factor plasticity rules have been explored in the literature. One important model can be found in Xie and Seung (2004); while our learning rules are generally built off of simpler two-factor rules modified to incorporate dopaminergic feedback, they derive their learning rule directly from gradient ascent applied to a reward signal. Another work modeling dopamine-dependent STDP is Izhikevich (2007). The plasticity rule in that work closely resembles our additive model. However, while we focus on the corticostriatal synapses and employ a simple setting consisting of a population of cortical neurons connected to a single striatal neuron, they instead use a mixed population of excitatory and inhibitory neurons with random connectivity meant to model part of a cortical column. The scenarios that they use to test their model also differ from ours. For a more detailed review of other work on three-factor plasticity rules, see Frémaux and Gerstner (2016); Gerstner et al. (2018).

What are the implications of our findings for models of the basal ganglia? We showed that each model has some settings in which it does well and some settings where it fails to accomplish the given task. There are several potential explanations for these outcomes. It is possible that the plasticity mechanism used in the corticostriatal synapses incorporates features that are not well-captured by any of the models considered here. It is also possible that the simplified models that we consider omit aspects of the computational structure of the basal ganglia that are crucial for functional performance. For instance, we do not model the competition between direct and indirect pathways through the basal ganglia, nor the differing effects of dopamine on the two pathways (spiny projection neurons in the direct pathway primarily express the D1 receptor, for which higher dopamine levels lead to LTP and lower dopamine levels lead to LTD and which form the basis for the corticostriatal plasticity model considered here, while in the indirect pathway they primarily express the D2 receptor, for which higher dopamine levels lead to LTD and lower dopamine levels lead to LTP Shan et al. (2014); Shen et al. (2008)). There may also be more complexity to dopaminergic feedback than the simple model we use; for example, recent work suggests that the dopamine signal may be better modeled as multidimensional rather than scalar-valued Wärnberg and Kumar (2023). An exciting future direction would be to extend our analysis to take more of these subtleties into account. Nevertheless, we believe that the settings we studied are general enough that our results will apply to more detailed models.

An interesting possible implication of our work is that different regions of the striatum may feature different plasticity mechanisms specialized to their particular roles. For instance, the ventral and dorsal striatum, which primarily contribute to reward prediction and action selection, respectively O’Doherty et al. (2004), may use distinct plasticity rules tuned to the specific tasks that they perform, as suggested by experimental evidence Perez et al. (2022); Wang (2008). More generally, while we have focused in this paper on the corticostriatal connections, our settings are broad enough that they may apply to any other region of the brain that receives dopaminergic signals, such as the prefrontal cortex where dopamine-dependent plasticity also occurs Otani et al. (2003). The random dopamine setting should be relevant whenever the dopamine signal is independent of a neuron’s output, the reward prediction setting applies to any task in which a neuron must match a target firing rate in order to minimize a dopamine error signal, and the action selection setting is a fairly broad model of learning dynamics under competition between two channels. Thus, the fact that no plasticity rule performed well in every setting in our study may simply be due to the specialization of different regions for the specific computational functions that they perform.

## Figures and Tables

**Fig. 15:**
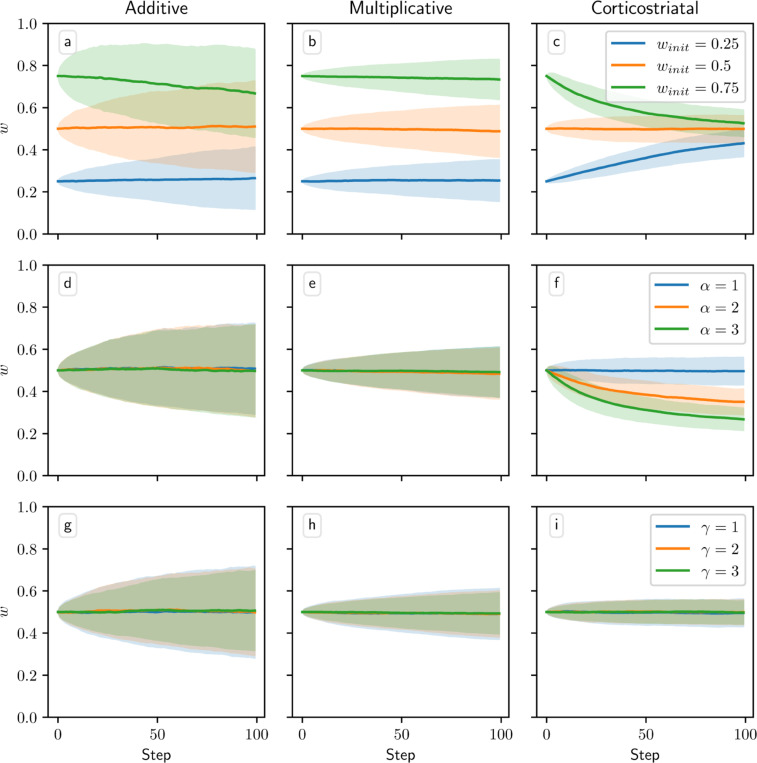
Weight evolution over time in the random dopamine setting for single-trace models. Columns show the additive (a, d, g), multiplicative (b, e, h), and corticostriatal (c, f, i) models. (a-c) the initial weight $w_{\text{init}}$ is varied while α=1 and γ=1 are fixed. (d-f) α is varied while $w_{\text{init}}=0.5$ and γ=1 are fixed. (g-i) γ is varied while $w_{\text{init}}=0.5$ and α=1 are fixed

**Fig. 16:**
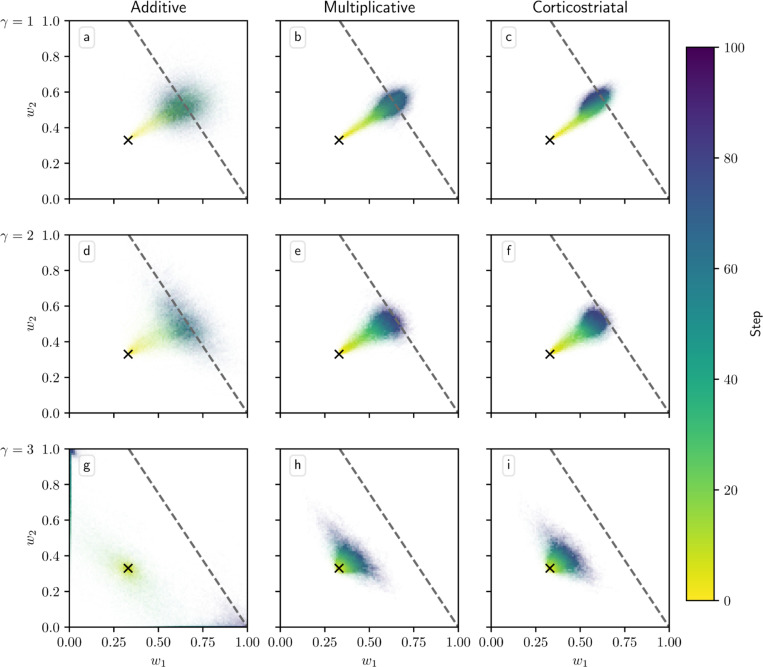
Distribution of weights over time in the reward prediction setting as γ is varied for single-trace models. Columns show the additive (a, d, g), multiplicative (b, e, h), and corticostriatal (c, f, i) models. γ is varied across rows: (a-c) γ=1; (d-f) γ=2; (g-i) γ=3. We include the solution planes for reference, but as we do not have averaged forms of the single-trace dynamics we do not include vector fields or fixed points or analyze the stability of the solution planes

**Fig. 17:**
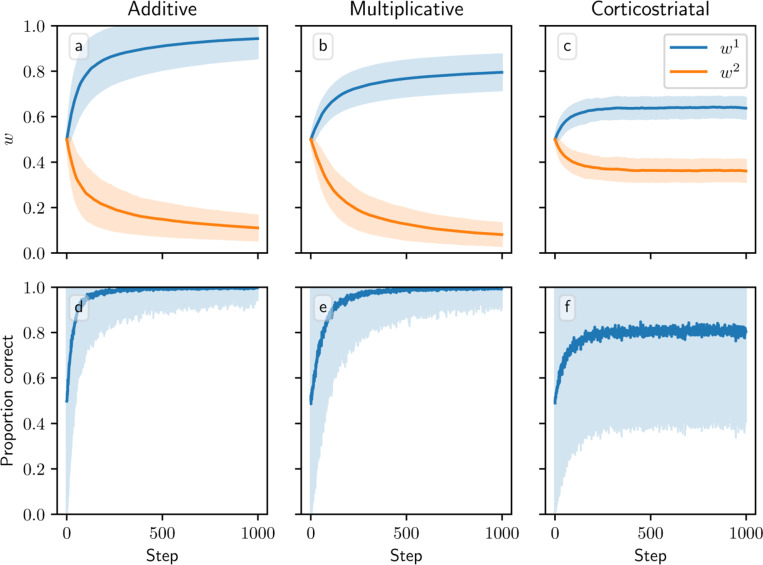
Model performance in the action selection setting for single-trace models. Plots show weights (a-c) and probability of taking the correct action (d-f) versus time for the additive (a, d), multiplicative (b, e), and corticostriatal (c, f) models. In these simulations γ=1

**Fig. 18:**
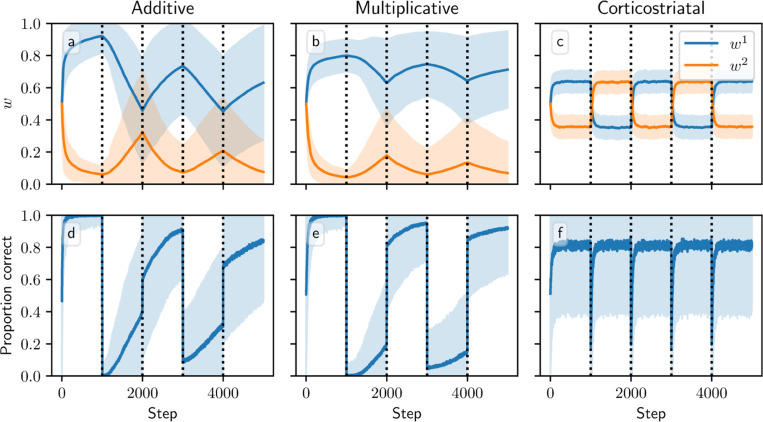
Model performance in the action selection setting with contingency switching for single-trace models. Plots show weights (a-c) and probability of taking the correct action (d-f) versus time for the additive (a, d), multiplicative (b, e), and corticostriatal (c, f) models. Here γ=1, and the other parameters are the same as those used in Figure 10

**Table 1:** Scaling factors for the three main models for positive and negative dopamine and eligibility

	Additive		Multiplicative		Corticostriatal	
_$E_{i}(t)$_╲^D(t)^	−	+	−	+	−	+
−	α	α	αw	αw	1−w	αw
+	1	1	1−w	1−w	αw	1−w

Blue cells correspond to scenarios in which the weights will increase, while orange indicates that the weights will decrease.

**Table 2:** Default simulation parameters for the three main task settings

	Random Dopamine	Reward Prediction	Action Selection
Samples	1000	1000	1000
Steps	100	100	1000
λ	0.01	0.0033	0.025
$w_{\text{init}}$	0.5	0.33 or 0.5	0.5
N	1	2	1
r	5 s^−1^	(15,10) s^−1^	10 s^−1^
$R^{*}$	N/A	7.5	N/A
$R_{1}^{*},\;R_{2}^{*}$	N/A	N/A	(2, 1)
α	1	1	1
τ	0.02 s	0.02 s	0.02 s
$\tau_{\text{dop}}$	1 s	1 s	1 s
$\tau_{\text{eli}}$	1 s	1 s	1 s
$T_{\text{del}}$	N/A	3 s	0 s
$T_{\text{win}}$	N/A	1 s	1 s
ϵ	0.001 s	0.001 s	0.001 s
$r^{\text{dop}}$	1/6 s^−1^	1/7 s^−1^	1/7 s^−1^
β	N/A	N/A	10^5^
$\sigma_{\text{dop}}$	1	N/A	N/A

## Appendix A Averaged Model, Reward Prediction Setting

### A.1 Additive and Multiplicative Models

Here we derive an averaged model that adds up all pre-post spike pairs and takes the average over realizations of the pre- and postsynaptic spike trains and over the dopamine signal, focusing on the additive and multiplicative models in the reward rate setting. (The presentation here largely follows that in Gütig et al. (2003).) We first give an expression for the total change in weight induced by a single triplet of a presynaptic spike at $t_{\text{pre}}$, a postsynaptic spike at $t_{\text{post}}$, and a dopamine signal D at $t_{\text{dop}}$. This can be found by integrating over the time since the largest of $t_{\text{pre}}$, $t_{\text{post}}$, and $t_{\text{dop}}$, because prior to $t_{\text{pre}}$ or $t_{\text{post}}$, the eligibility trace is zero, and prior to $t_{\text{dop}}$, the dopamine trace is zero. The result is given here for the additive and multiplicative models:

(A1)
$$\text{Δ}w=\lambda D\int_{\max\left\{t_{\text{dop}},t_{\text{pre}},t_{\text{post}}\right\}}^{\infty }e^{-\frac{t-t_{\text{dop}}}{\tau_{\text{dop}}}}e^{-\frac{t-\max\left\{t_{\text{pre}},t_{\text{post}}\right\}}{\tau_{\text{eli}}}}e^{-\frac{|t_{\text{post}}-t_{\text{pre}}|}{\tau }}\;\;\times \left(\{\begin{array}{ll} -f_{-}(w) & \text{if}\;t_{\text{post}}\leq t_{\text{pre}}\\ f_{+}(w) & \text{if}\;t_{\text{post}}>t_{\text{pre}} \end{array}\right)dt\;\;=\lambda D\frac{\tau_{\text{dop}}\tau_{\text{eli}}}{\tau_{\text{dop}}+\tau_{\text{eli}}}e^{-\frac{|t_{\text{post}}-t_{\text{pre}}|}{\tau }}\left(\text{\{}\begin{array}{ll} -f_{-}(w) & \text{if}\;t_{\text{post}}\leq t_{\text{pre}}\\ f_{+}(w) & \text{if}\;t_{\text{post}}>t_{\text{pre}} \end{array}\right)\;\;\times \left(\begin{array}{ll} e^{-\frac{|t_{\text{dop}}-\max\left\{t_{\text{pre}},t_{\text{post}}\right\}|}{\tau_{\text{dop}}}} & \text{if}\;t_{\text{dop}}\leq \max\left\{t_{\text{pre}},t_{\text{post}}\right\}\\ e^{-\frac{|t_{\text{dop}}-\max\left\{t_{\text{pre}},t_{\text{post}}\right\}|}{\tau_{\text{eli}}}} & \text{if}\;t_{\text{dop}}>\max\left\{t_{\text{pre}},t_{\text{post}}\right\} \end{array}\right).$$

We restate here the definition of the dopamine signal for the reward prediction setting:

(A2)
$$D=R^{*}-\bar{R}$$

where

(A3)
$$\bar{R}=\frac{1}{T_{\text{win}}}\int_{t_{\text{dop}}-T_{\text{win}}-T_{\text{del}}}^{t_{\text{dop}}-T_{\text{del}}}\rho^{\text{post}}(t)dt.$$

Following Gütig et al. (2003), we will define the cross-correlation functions ${\text{Γ}}_{i,\text{post}}(\text{Δ}t)={\langle \rho_{i}^{\text{pre}}(t)\rho^{\text{post}}(t+\text{Δ}t)\rangle }_{t}$, where ${\langle \cdot \rangle }_{t}$ denotes averaging over time. These will arise in our derivation of the averaged weight dynamics. We also define the point process $\rho^{\text{dop}}$ indicating when a dopamine signal is delivered, with rate ${\langle \rho^{\text{dop}}(t)\rangle }_{t}=r^{\text{dop}}$. (As noted previously, in simulations we assume for simplicity that dopamine is delivered periodically, but the precise form of the dopamine process does not matter as long as it has the given mean rate, it is independent of the spike trains, and dopamine signals are far enough apart that their interactions can be neglected.) Treating Δw as a function of $t_{\text{pre}}$, $t_{\text{post}}$, and $t_{\text{dop}}$, we can write the mean weight drift as follows:

(A4)
$${\dot{w}}_{i}={\langle \int }_{-\infty }^{\infty }\int_{-\infty }^{\infty }\text{Δ}w_{i}(t,t+\text{Δ}t,t+\text{Δ}t+\text{Δ}s)\;\;\rho_{i}^{\text{pre}}(t)\rho^{\text{post}}(t+\text{Δ}t)\rho^{\text{dop}}(t+\text{Δ}t+\text{Δ}s)\;d\text{Δ}s\;d\text{Δ}{t\rangle }_{t}$$

where $t=t_{\text{pre}},\;\text{Δ}t=t_{\text{post}}-t_{\text{pre}}$, and $\text{Δ}s=t_{\text{dop}}-t_{\text{post}}$.

Note that $\rho^{\text{dop}}$ is independent of the other terms. Also, if $T_{\text{del}}$ is large enough, we can assume that $\bar{R}$ (and hence D) is independent of $\rho^{\text{post}}$ (and hence also of $\rho_{i}^{\text{pre}}$, as $\bar{R}$ only depends on $\rho_{i}^{\text{pre}}$ through $\rho^{\text{post}}$), because any postsynaptic spikes counted by the integral in equation (A3) must occur at least $T_{\text{del}}$ before the dopamine signal, and consequently, either the negative exponential in $|t_{\text{dop}}-\max\left\{t_{\text{pre}},t_{\text{post}}\right\}|$ or the one in $|t_{\text{post}}-t_{\text{pre}}|$ in equation (A1) will be very small. Thus, assuming D is independent of the other terms provides a very good approximation if $T_{\text{del}}$ is large enough. Another simplifying assumption we will make is that the weights change only a small amount on each dopamine release, so that we can treat $w_{i}$ as constant in these expressions. Under these assumptions, we can substitute equations (A1) to (A3) into equation (A4) and split it into the $t_{\text{post}}\leq t_{\text{pre}}$ and $t_{\text{post}}>t_{\text{pre}}$ cases as follows:

(A5)
$${\dot{w}}_{i}=-\lambda f_{-}\left(w_{i}\right)\int_{-\infty }^{0}\int_{-\infty }^{\infty }\left(R^{*}-\frac{1}{T_{\text{win}}}\int_{0}^{T_{\text{win}}}{\langle \rho^{\text{post}}\left(u+t+\text{Δ}t+\text{Δ}s-T_{\text{del}}-T_{\text{win}}\right)\rangle }_{t}du\right)\;\;\times \frac{\tau_{\text{dop}}\tau_{\text{eli}}}{\tau_{\text{dop}}+\tau_{\text{eli}}}e^{-\frac{\left|\text{Δ}t\right|}{\tau }}(\text{\{}\begin{array}{cc} e^{-\frac{\left|\text{Δ}s+\text{Δ}t\right|}{\tau_{\text{dop}}}} & \text{if}\;\text{Δ}s+\text{Δ}t\leq 0\\ e^{-\frac{\left|\text{Δ}s+\text{Δ}t\right|}{\tau_{\text{eli}}}} & \text{if}\;\text{Δ}s+\text{Δ}t>0 \end{array}\text{)}\;\;\times {\langle \rho_{i}^{\text{pre}}(t)\rho^{\text{post}}(t+\text{Δ}t)\rangle }_{t}{\langle \rho^{\text{dop}}(t+\text{Δ}t+\text{Δ}s)\rangle }_{t}d\text{Δ}s\;d\text{Δ}t\;+\lambda f_{+}\left(w_{i}\right)\int_{0}^{\infty }\int_{-\infty }^{\infty }\left(R^{*}-\frac{1}{T_{\text{win}}}\int_{0}^{T_{\text{win}}}{\langle \rho^{\text{post}}(u+t+\text{Δ}t+\text{Δ}s-T_{\text{del}}-T_{\text{win}})\langle }_{t}\;du\right)\;\;\times \frac{\tau_{\text{dop}}\tau_{\text{eli}}}{\tau_{\text{dop}}+\tau_{\text{eli}}}e^{-\frac{\left|\text{Δ}t\right|}{\tau }}\left(\text{\{}\begin{array}{ll} e^{-\frac{\left|\text{Δ}s\right|}{\tau_{\text{dop}}}} & \text{if Δ}s\leq 0\\ e^{-\frac{\left|\text{Δ}s\right|}{\tau_{\text{eli}}}} & \text{if Δ}s>0 \end{array}\right)\;\;\times {\langle \rho_{i}^{\text{pre}}(t)\rho^{\text{post}}(t+\text{Δ}t)\rangle }_{t}{\langle \rho^{\text{dop}}(t+\text{Δ}t+\text{Δ}s)\rangle }_{t}d\text{Δ}s\;d\text{Δ}t.$$

Recall that the postsynaptic firing rate is given by

$$R(t)=\frac{1}{N}\sum_{i=1}^{N}w_{i}(t)\rho_{i}^{\text{pre}}(t-\epsilon ).$$

It follows that for any x,

(A6)
$${\langle \rho }^{\text{post}}(t+x{)\rangle }_{t}=\frac{1}{N}\sum_{i=1}^{N}w_{i}{\langle \rho_{i}^{\text{pre}}(t+x-\epsilon )\rangle }_{t}\;\;=\frac{1}{N}\sum_{i=1}^{N}w_{i}r_{i},$$

in particular this applies to ${\langle \rho^{\text{post}}\left(u+t+\text{Δ}t+\text{Δ}s-T_{\text{del}}-T_{\text{win}}\right)\rangle }_{t}$ in equation (A5). Additionally, ${\langle \rho^{\text{dop}}(t+\text{Δ}t+\text{Δ}s)\rangle }_{t}=r^{\text{dop}}$ is a constant. We can therefore make the change of variables Δs←Δs+Δt to combine the positive and negative integrals, arriving at the formula:

$${\dot{w}}_{i}=\left(R^{*}-\frac{1}{N}\sum_{i=1}^{N}w_{i}r_{i}\right)r^{\text{dop}}\frac{\tau_{\text{dop}}\tau_{\text{eli}}}{\tau_{\text{dop}}+\tau_{\text{eli}}}\int_{-\infty }^{\infty }(\text{\{}\begin{array}{cc} e^{-\frac{\left|\text{Δ}s\right|}{\tau_{\text{dop}}}} & \text{if Δ}s\leq 0\\ e^{-\frac{\left|\text{Δ}s\right|}{\tau_{\text{eli}}}} & \text{if Δ}s>0 \end{array}\text{)}\;d\text{Δ}s\;\;\times \int_{-\infty }^{\infty }e^{-\frac{|\text{Δ}t|}{\tau }}(\text{\{}\begin{array}{ll} -\lambda f_{-}\left(w_{i}\right) & \text{if Δ}t\leq 0\\ \lambda f_{+}\left(w_{i}\right) & \text{if Δ}t>0 \end{array}{\text{)Γ}}_{i,\text{post}}(\text{Δ}t)d\text{Δ}t\;=\left(R^{*}-\frac{1}{N}\sum_{i=1}^{N}w_{i}r_{i}\right)r^{\text{dop}}\tau_{\text{dop}}\tau_{\text{eli}}\;\;\times \int_{-\infty }^{\infty }e^{-\frac{\left|\text{Δ}t\right|}{\tau }}(\text{\{}\begin{array}{ll} -\lambda f_{-}\left(w_{i}\right) & \text{if Δ}t\leq 0\\ \lambda f_{+}\left(w_{i}\right) & \text{if Δ}t>0 \end{array}{)\text{Γ}}_{i,\text{post}}(\text{Δ}t)d\text{Δ}t.$$

Note that the remaining integral is exactly the one found in Gütig et al. (2003). Using equation (A6) and following Gütig et al. (2003), we decompose ${\text{Γ}}_{i,\text{post}}$ as

$${\text{Γ}}_{i,\text{post}}(\text{Δ}t)=\frac{1}{N}\sum_{j=1}^{N}w_{j}{\langle \rho_{i}^{\text{pre}}(t)\rho_{j}^{\text{pre}}(t+\text{Δ}t-\epsilon )\rangle }_{t}$$

and define the normalized cross-correlation function

$${\text{Γ}}_{ij}^{0}\left(t^{\prime }\right)=\frac{{\langle \rho_{i}^{\text{pre}}(t)\rho_{j}^{\text{pre}}\left(t+t^{\prime }\right)\rangle }_{t}}{r_{i}r_{j}}-1.$$

(Note that Gütig et al. (2003) assumes all presynaptic firing rates are identical, and so uses $r^{2}$ in the denominator instead.) We also define the effective cross-correlation matrices $C^{\pm }$ with elements

$$C_{ij}^{+}=\int_{0}^{\infty }\frac{1}{\tau }e^{-\frac{\left|\text{Δ}t\right|}{\tau }}{\text{Γ}}_{ij}^{0}(\text{Δ}t-\epsilon )d\text{Δ}t$$

and similarly for $C_{ij}^{-}$ (which integrates from −∞ to 0). Then we can rewrite the integrals in terms of $C_{ij}^{\pm }$:

$$\lambda f_{+}\left(w_{i}\right)\int_{0}^{\infty }e^{-\frac{\left|\text{Δ}t\right|}{\tau }}{\text{Γ}}_{i,\text{post}}(\text{Δ}t)d\text{Δ}t=\lambda f_{+}\left(w_{i}\right)\frac{1}{N}\sum_{j=1}^{N}w_{j}\tau r_{i}r_{j}\;\;\times \left(1+\int_{0}^{\infty }\frac{1}{\tau }e^{-\frac{\left|\text{Δ}t\right|}{\tau }}{\text{Γ}}_{ij}^{0}(\text{Δ}t-\epsilon )d\text{Δ}t\right)\;\;=\lambda f_{+}\left(w_{i}\right)\frac{1}{N}\sum_{j=1}^{N}w_{j}\tau r_{i}r_{j}\left(1+C_{ij}^{+}\right)$$

and similarly for the negative terms. Like in Gütig et al. (2003), we assume ${\text{Γ}}_{ij}^{0}\left(t^{\prime }\right)=\frac{1}{\sqrt{r_{i}r_{j}}}c_{ij}\delta \left(t^{\prime }\right)$ for some constants $c_{ij}\geq 0$ (again extending their formula to non-identical presynaptic firing rates). Since the argument of ${\text{Γ}}_{ij}^{0}(\text{Δ}t-\epsilon )$ is never zero when Δt<0, it follows that $C_{ij}^{-}=0$ and $C_{ij}^{+}=\frac{1}{\tau \sqrt{r_{i}r_{j}}}c_{ij}e^{-\epsilon /\tau }\approx \frac{1}{\tau \sqrt{r_{i}r_{j}}}c_{ij}$. (We assume, as in Gütig et al. (2003), that ϵ is small enough that $e^{-\epsilon /\tau }\approx 1$.) For Possion spike trains, the constants $c_{ij}$ equal 1 if the spike trains are identical (because the autocorrelation is ${\langle \rho (t)\rho \left(t+t^{\prime }\right)\rangle }_{t}=r^{2}+r\delta \left(t^{\prime }\right)$ for a Poisson spike train ρ with rate r) and are otherwise less than 1. We will assume that the presynaptic spike trains are uncorrelated, so $c_{ij}=0$ for i≠j. Therefore the formulas simplify as follows:

$$\lambda f_{+}\left(w_{i}\right)\frac{1}{N}\sum_{j=1}^{N}w_{j}\tau r_{i}r_{j}\left(1+C_{ij}^{+}\right)=\lambda f_{+}\left(w_{i}\right)\frac{1}{N}\left(w_{i}r_{i}+\sum_{j=1}^{N}w_{j}\tau r_{i}r_{j}\right)$$

and

$$-\lambda f_{-}\left(w_{i}\right)\frac{1}{N}\sum_{j=1}^{N}w_{j}\tau r_{i}r_{j}\left(1+C_{ij}^{-}\right)=-\lambda f_{-}\left(w_{i}\right)\frac{1}{N}\sum_{j=1}^{N}w_{j}\tau r_{i}r_{j}.$$

Substituting these results back in, we obtain the formula for ${\dot{w}}_{i}$:

$${\dot{w}}_{i}=\left(R^{*}-\frac{1}{N}\sum_{j=1}^{N}w_{i}r_{i}\right)r^{\text{dop}}\tau_{\text{dop}}\tau_{\text{eli}}\frac{\lambda }{N}\left(\tau \text{Δ}f\left(w_{i}\right)r_{i}\left(\sum_{j=1}^{N}w_{j}r_{j}\right)+f_{+}\left(w_{i}\right)w_{i}r_{i}\right)$$

where $\text{Δ}f=f_{+}-f_{-}.$ In vector notation, this can be written as:

(A7)
$$\dot{w}=\left(R^{*}-\frac{1}{N}\langle w,r\rangle \right)r^{\text{dop}}\tau_{\text{dop}}\tau_{\text{eli}}\frac{\lambda }{N}\left(\tau \langle w,r\rangle \text{Δ}f(w)⊙r+f_{+}(w)⊙w⊙r\right)$$

where ⊙ is the entrywise or Hadamard product and we treat $f_{\pm }(w)$ as applying entrywise.

### A.2 Corticostriatal Model

The analogous expression to equation (A1) for the corticostriatal model is:

$$\text{Δ}w=\lambda \frac{\tau_{\text{dop}}\tau_{\text{eli}}}{\tau_{\text{dop}}+\tau_{\text{eli}}}e^{-\frac{|t_{\text{post}}-t_{\text{pre}}|}{\tau }}\left(\text{\{}\begin{array}{ll} -\alpha |D|w & \text{if}\;D\left(t_{\text{post}}-t_{\text{pre}}\right)\leq 0\\ \left|D|(1-w\right) & \text{if}\;D\left(t_{\text{post}}-t_{\text{pre}}\right)>0 \end{array}\right)\;\;\times \left(\text{\{}\begin{array}{ll} e^{-\frac{|t_{\text{dop}}-\max\left\{t_{\text{pre}},t_{\text{post}}\right\}|}{\tau_{\text{dop}}}} & \text{if}\;t_{\text{dop}}\leq \max\left\{t_{\text{pre}},t_{\text{post}}\right\}\\ e^{-\frac{|t_{\text{dop}}-\max\left\{t_{\text{pre}},t_{\text{post}}\right\}|}{\tau_{\text{eli}}}} & \text{if}\;t_{\text{dop}}>\max\left\{t_{\text{pre}},t_{\text{post}}\right\} \end{array}\right).$$

To derive an averaged form of the corticostriatal model, we need to decompose the expected dopamine signal into $E[D]=D_{+}+D_{-}$, where

$$D_{+}=E[D|D\geq 0]P(D\geq 0)\;D_{-}=E[D|D<0]P(D<0).$$

These can be computed by counting the number of postsynaptic spikes to fall inside the window in equation (A3), using the cumulative distribution function of the Poisson distribution; on the D≥0 side,

$$D_{+}=\sum_{n=0}^{\left\lfloor R^{*}T_{\text{win}}\right\rfloor }\left(R^{*}-\frac{n}{T_{\text{win}}}\right)\frac{{\left(r^{\text{post}}T_{\text{win}}\right)}^{n}e^{-r^{\text{post}}T_{\text{win}}}}{n!}\;\;=R^{*}\sum_{n=0}^{\left\lfloor R^{*}T_{\text{win}}\right\rfloor }\frac{{\left(r^{\text{post}}T_{\text{win}}\right)}^{n}e^{-r^{\text{post}}T_{\text{win}}}}{n!}-r^{\text{post}}\sum_{n=1}^{\left\lfloor R^{*}T_{\text{win}}\right\rfloor }\frac{{\left(r^{\text{post}}T_{\text{win}}\right)}^{n-1}e^{-r^{\text{post}}T_{\text{win}}}}{(n-1)!}\;\;=R^{*}\frac{\text{Γ}\left(\left\lfloor R^{*}T_{\text{win}}\right\rfloor +1,r^{\text{post}}T_{\text{win}}\right)}{\text{Γ}\left(\lfloor R^{*}T_{\text{win}}\rfloor +1\right)}-r^{\text{post}}\frac{\text{Γ}\left(\left\lfloor R^{*}T_{\text{win}}\right\rfloor ,r^{\text{post}}T_{\text{win}}\right)}{\text{Γ}\left(\left\lfloor R^{*}T_{\text{win}}\right\rfloor \right)}$$

where $r^{\text{post}}=\frac{1}{N}\langle w,r\rangle$ is the postsynaptic firing rate. Since $E[D]=R^{*}-r^{\text{post}}$, it follows that $D_{-}=R^{*}-r^{\text{post}}-D_{+}$. Then an analogous derivation to that in Section A.1, treating the D≥0 and D<0 cases separately, gives the following average drift formula:

(A8)
$$\dot{w}=r^{\text{dop}}\tau_{\text{dop}}\tau_{\text{eli}}\frac{\lambda }{N}(D_{+}(\tau \langle w,r\rangle (1-(1+\alpha )w)⊙r+(1-w)⊙w⊙r)\;\;-D_{-}(\tau \langle w,r\rangle (1-(1+\alpha )w)⊙r-\alpha w⊙w⊙r))$$

## Appendix B Stability of Solution Equilibria, Reward Prediction Setting

### B.1 Stability Condition

In the reward prediction setting the additive and multiplicative models have equilibria along the solution plane, defined as the set of weights such that $\frac{1}{N}\langle w,r\rangle =R^{*}$. However, these equilibria are not necessarily stable. We will now describe the conditions under which some or all of the solution plane is stable. We are particularly interested in conditions under which for any pair $r,R^{*}$ there exists some weight w such that $\frac{1}{N}\langle w,r\rangle =R^{*}$ is a stable equilibrium. (Note that if $R^{*}>\frac{1}{N}{\sum_{i=1}^{N}r}_{i}$ then this condition is impossible to satisfy, as the weights are restricted to [0,1]. We will therefore always assume that $0\leq R^{*}\leq \frac{1}{N}{\sum_{i=1}^{N}r}_{i}$.) We will first derive a general stability condition for the additive and multiplicative models and describe its application to these models.

For the additive and multiplicative models, the Jacobian on the plane $\frac{1}{N}\langle w,r\rangle =R^{*}$ is simple to calculate, as the derivatives of the second term in equation (A7) are multiplied by $R^{*}-\frac{1}{N}\langle w,r\rangle$ and therefore go to zero. The Jacobian is then given by:

$$J=-\frac{1}{N}\times r^{\text{dop}}\tau_{\text{dop}}\tau_{\text{eli}}\frac{\lambda }{N}\left(\tau \langle w,r\rangle \text{Δ}f(w)⊙r+f_{+}(w)⊙w⊙r\right)r^{T}\;\;=-r^{\text{dop}}\tau_{\text{dop}}\tau_{\text{eli}}\frac{\lambda }{N}\left(\tau R^{*}\text{Δ}f(w)⊙r+\frac{1}{N}f_{+}(w)⊙w⊙r\right)r^{T}.$$

The Jacobian has the eigenvalue 0 with multiplicity N−1 corresponding to the subspace orthogonal to r, that is, parallel to the solution plane. The remaining eigenvalue is given by

$$\text{Λ}=-r^{\text{dop}}\tau_{\text{dop}}\tau_{\text{eli}}\frac{\lambda }{N}\langle \tau R^{*}\text{Δ}f(w)⊙r+\frac{1}{N}f_{+}(w)⊙w⊙r,r\rangle$$

with associated eigenvector

$$\tau R^{*}\text{Δ}f(w)⊙r+\frac{1}{N}f_{+}(w)⊙w⊙r.$$

To determine the stability of the solution plane we therefore simply need to examine the sign of Λ, giving the following *stability condition*:

(B9)
$$0<\langle \tau R^{*}\text{Δ}f(w)⊙r+\frac{1}{N}f_{+}(w)⊙w⊙r,r\rangle \;\;=\sum_{i=1}^{N}r_{i}^{2}\left(\tau R^{*}\text{Δ}f\left(w_{i}\right)+\frac{1}{N}f_{+}\left(w_{i}\right)w_{i}\right).$$

Note that in equation (B9) we can substitute $R^{*}=\frac{1}{N}\langle w,r\rangle$ as long as we are on the solution plane, giving the equivalent condition

(B10)
$$0<\langle \tau \langle w,r\rangle \text{Δ}f(w)⊙r+f_{+}(w)⊙w⊙r,r\rangle .$$

Equations (B9) and (B10) define different subsets of ${\left[0,1\right]}^{N}$ but identical sets when restricted to the plane $R^{*}=\frac{1}{N}\langle w,r\rangle$. We can therefore use either condition depending on which is more convenient for any particular calculation.

### B.2 Sufficient Condition for a Stable Solution

We can derive a general sufficient condition for the existence of a stable solution for both the additive and multiplicative models, restating and proving Theorem 1. In all of the following analysis we assume at least one $r_{i}$ is nonzero, as the r=0 case is trivial.

#### Theorem 1.

*Pick* $r\in {\mathbb{R}}^{N}$ *and* $R^{*}\leq \frac{1}{N}∥r{∥}_{1}$, *and let* $w^{\prime }=NR^{*}/∥r{∥}_{1}$. *If*

(10)
$$f_{-}\left(w^{\prime }\right)<\left(1+\frac{1}{\tau ∥r{∥}_{1}}\right)f_{+}\left(w^{\prime }\right),$$

*then there exists a stable point on the solution plane, given by* $w=\left(w^{\prime },\ldots ,w^{\prime }\right)$.

*Proof*. First note that $w=\left(w^{\prime },\ldots ,w^{\prime }\right)$ clearly lies on the solution plane, because $\frac{1}{N}\langle \left(w^{\prime },\ldots ,w^{\prime }\right),r\rangle =\frac{1}{N}w^{\prime }∥r{∥}_{1}=R^{*}$. A sufficient condition for equation (B10) to hold at the point $\left(w^{\prime },\ldots ,w^{\prime }\right)$ is that for all i,

$$\;0<r_{i}^{2}\left(\tau \langle (w^{\prime },\ldots ,w^{\prime }),r\rangle \left(f_{+}\left(w^{\prime }\right)-f_{-}\left(w^{\prime }\right)\right)+f_{+}\left(w^{\prime }\right)w^{\prime }\right)\;\;=r_{i}^{2}\left(\tau w^{\prime }∥r{∥}_{1}\left(f_{+}\left(w^{\prime }\right)-f_{-}\left(w^{\prime }\right)\right)+f_{+}\left(w^{\prime }\right)w^{\prime }\right)\;\Leftrightarrow 0<\tau ∥r{∥}_{1}\left(f_{+}\left(w^{\prime }\right)-f_{-}\left(w^{\prime }\right)\right)+f_{+}\left(w^{\prime }\right)$$

and rearranging the terms gives equation (10). □

In the case of the additive model, $f_{+}(w)=1$ and $f_{-}=\alpha$, so rearranging equation (10) gives the following condition:

(B11)
$$\tau (\alpha -1)<\frac{1}{∥r{∥}_{1}}.$$

We can also derive a condition for the multiplicative model, where $f_{+}(w)=1-w$ and $f_{-}(w)=\alpha w$, by plugging the definition of $w^{\prime }$ into equation (10):

$$\alpha \frac{NR^{*}}{∥r{∥}_{1}}<\left(1+\frac{1}{\tau ∥r{∥}_{1}}\right)\left(1-\frac{NR^{*}}{∥r{∥}_{1}}\right)\;\Leftrightarrow R^{*}<\frac{1}{N}∥r{∥}_{1}\frac{1+1/\tau ∥r{∥}_{1}}{\alpha +1+1/\tau ∥r{∥}_{1}}\;\;=\frac{w_{0}}{N}∥r{∥}_{1}$$

where

(B12)
$$w_{0}=\frac{\tau ∥r{∥}_{1}+1}{\tau (1+\alpha )∥r{∥}_{1}+1}.$$

The point $w=\left(w_{0},\ldots ,w_{0}\right)$ is in fact a fixed point of the multiplicative model, as can be seen by plugging it into equation (A7), and will be discussed in more detail in Section C.2.

### B.3 Additive Model

For the additive model we can also derive a necessary condition for the existence of a stable solution. Here, $f_{+}(w)=1$ and $f_{-}(w)=\alpha$, so we can write the stability condition (equation (B10)) as follows:

0<〈τ(1−α)〈w,r〉r+w⊙r,r〉=τ(1−α)〈w,r〉〈r,r〉+〈w,r⊙r〉=〈w,τ(1−α)〉〈r,r〉r+r⊙r〉

where we have used the fact that 〈x⊙y,z〉=〈x,y⊙z〉 for real vectors. Note that this defines a half-space within the space of weights with the origin on the boundary; we would like to find conditions under which at least some part of the solution plane in ${\left[0,1\right]}^{N}$ lies inside this half-space. A very simple *necessary condition* for this to take place is that the intersection of this half-space with ${\left[0,1\right]}^{N}$ is non-empty. This is equivalent to requiring that the vector τ(1−α)〈r,r〉r+r⊙r (the normal vector to the boundary of the half-space) has at least one positive entry. In other words, there exists some index i such that

$$\;0<\tau (1-\alpha )\langle r,r\rangle r_{i}+r_{i}^{2}\;\Leftrightarrow 0<\tau (1-\alpha )\langle r,r\rangle +r_{i}$$

if $r_{i}\neq 0$. Since $r_{i}\geq 0$ for all i, this is equivalent to a condition on the infinity norm of r:

(B13)
$$\tau (\alpha -1)<\frac{∥r{∥}_{\infty }}{∥r{∥}_{2}^{2}}.$$

Note that the right-hand-side of equation (B13) goes to zero as r grows, so if α>1, then we cannot put a condition on the parameters α and τ guaranteeing that the necessary condition holds for all r; however, we can do so if we restrict ourselves to input rate vectors r with bounded norm. Suppose $∥r{∥}_{1}\leq r_{\max}$. Then we have:

$$\frac{∥r{∥}_{\infty }}{∥r{∥}_{2}^{2}}=\frac{1}{\sum_{i=1}^{N}\frac{r_{i}^{2}}{\underset{j}{\max}\left\{r_{j}\right\}}}\;\;\geq \frac{1}{\sum_{i=1}^{N}r_{i}}\;\;\geq \frac{1}{r_{\max}}.$$

(This lower bound is achieved at $r=\left(\frac{1}{N}r_{\max},\ldots ,\frac{1}{N}r_{\max}\right)$ and at $r=r_{\max}e_{i}$ for any coordinate vector $e_{i}$.) Thus the best bound on τ(α−1) that applies to all r such that $∥r{∥}_{1}\leq r_{\max}$ is $\frac{1}{r_{\max}}$. Combining this with equation (B11), we can state this result as follows:

#### Proposition 3.

*For the additive model, there exists some stable solution* w *(i.e.* $R^{*}=\frac{1}{N}\langle w,r\rangle$ *and the stability condition holds) for all* $r,R^{*}$ *such that* $R^{*}\leq \frac{1}{N}∥r{∥}_{1}$ *and* $∥r{∥}_{1}\leq r_{\max}$ *if and only if*

$$\tau (\alpha -1)<\frac{1}{r_{\max}}.$$

## Appendix C Other Dynamics Results, Reward Prediction Setting

### C.1 Stability of the Origin

For all three models, the origin w=0 is a fixed point in the reward prediction setting. Here we study its stability, focusing on the additive model for simplicity.

#### Proposition 4.

*For the additive model, the Jacobian at the fixed point* w=0 *is positive definite (and so the point is unstable) if and only if*

(C14)
$$\tau (\alpha -1)<\frac{1}{∥r{∥}_{1}}.$$

*Proof.* The Jacobian at w=0 can be calculated as follows, using equation (A7) and plugging in $f_{+}(w)=1$ and $f_{-}(w)=\alpha$:

$${\frac{\partial {\dot{w}}_{i}}{\partial w_{j}}|}_{w=0}=-\frac{1}{N}r_{j}r^{\text{dop}}\tau_{\text{dop}}\tau_{\text{eli}}\frac{\lambda }{N}\left(\tau (1-\alpha )r_{i}\langle r,w\rangle +w_{i}r_{i}\right)\;\;+{\left(R^{*}-\frac{1}{N}\langle w,r\rangle \right)r^{\text{dop}}\tau_{\text{dop}}\tau_{\text{eli}}\frac{\lambda }{N}\left(\tau (1-\alpha )r_{i}r_{j}+\delta_{ij}r_{i}\right)|}_{w=0}\;\;=R^{*}r^{\text{dop}}\tau_{\text{dop}}\tau_{\text{eli}}\frac{\lambda }{N}\left(\tau (1-\alpha )r_{i}r_{j}+\delta_{ij}r_{i}\right)$$

or in vector notation,

$$J_{0}=R^{*}r^{\text{dop}}\tau_{\text{dop}}\tau_{\text{eli}}\frac{\lambda }{N}\left(\tau (1-\alpha )rr^{T}+\text{diag}(r)\right).$$

Since $J_{0}$ is symmetric, we can apply Sylvester’s criterion to derive conditions under which $J_{0}$ is positive definite. If we assume $r_{i}\neq 0$ for each i so that diag (r) is invertible, then the determinant of $J_{0}$ can be computed using Sylvester’s determinant theorem:

$$\text{det}(J_{0})={\left(R^{*}r^{\text{dop}}\tau_{\text{dop}}\tau_{\text{eli}}\frac{\lambda }{N}\right)}^{N}\text{det}(\tau (1-\alpha )rr^{T}+\text{diag}(r))\;\;={\left(R^{*}r^{\text{dop}}\tau_{\text{dop}}\tau_{\text{eli}}\frac{\lambda }{N}\right)}^{N}\text{det diag}(r)\text{det}(\tau (1-\alpha )r^{T}\text{diag}{\left(r\right)}^{-1}r+I_{1})\;\;={\left(R^{*}r^{\text{dop}}\tau_{\text{dop}}\tau_{\text{eli}}\frac{\lambda }{N}\right)}^{N}(\prod_{i=1}^{N}r_{i})(\tau (1-\alpha )∥r{∥}_{1}+1).$$

This is positive if and only if $\tau (1-\alpha )∥r{∥}_{1}+1>0$. But note that every upper left submatrix of $J_{0}$ has the exact same structure, so analogous calculations show that the $k^{\text{th}}$ leading principal minor of $J_{0}$ is positive if and only if $1+\tau (1-\alpha ){\sum_{i=1}^{k}r}_{i}>0$. If α≤1 then this clearly holds for all k=1,…,N; if α>1 then $\tau (1-\alpha )\sum_{i=1}^{N}r_{i}>\tau (1-\alpha )\sum_{i=1}^{N}r_{i}$ (because $r_{i}\geq 0$ for all i), so we only need to check the $N^{\text{th}}$ term. Thus by rearranging this criterion, we see that $J_{0}$ is positive definite (and thus the fixed point w=0 is unstable) if and only if equation (C14) holds. □

### C.2 Extra Fixed Point in the Multiplicative Model

As noted above, the point $w=\left(w_{0},\ldots ,w_{0}\right)$ is a fixed point of the multiplicative model, where $w_{0}$ is defined in equation (B12). We can use a similar approach to that used previously to give conditions on its stability, stated in the main text as Theorem 2:

#### Theorem 2.

*For the multiplicative model, if*

$$R^{*}<\frac{w_{0}}{N}∥r{∥}_{1}$$

*then the Jacobian at the fixed point* $w=\left(w_{0},\ldots ,w_{0}\right)$ *is positive definite (and so the point is unstable); if*

$$R^{*}>\frac{w_{0}}{N}∥r{∥}_{1}$$

*then the Jacobian is negative definite (and so the point is stable)*.

*Proof.* The Jacobian can be computed as follows, using equation (A7) and plugging in $f_{+}(w)=1-w$ and $f_{-}(w)=\alpha w$:

$${\frac{\partial {\dot{w}}_{i}}{\partial w_{j}}|}_{w=\left(w_{0},\ldots ,w_{0}\right)}=-\frac{1}{N}r_{j}r^{\text{dop}}\tau_{\text{dop}}\tau_{\text{eli}}\frac{\lambda }{N}\left(\tau \left(1-(1+\alpha )w_{i}\right)r_{i}\langle r,w\rangle +\left(1-w_{i}\right)w_{i}r_{i}\right)\;\;+(R^{*}-\frac{1}{N}\langle w,r\rangle )r^{\text{dop}}\tau_{\text{dop}}\tau_{\text{eli}}\frac{\lambda }{N}(\tau \left(1-(1+\alpha )w_{i}\right)r_{i}r_{j}\;\;+\delta_{ij}\left(\left(1-2w_{i}\right)r_{i}-\tau (1+\alpha )r_{i}\langle w,r\rangle \right)){|}_{w=\left(w_{0},\ldots ,w_{0}\right)}\;\;=(R^{*}-\frac{1}{N}w_{0}∥r{∥}_{1})r^{\text{dop}}\tau_{\text{dop}}\tau_{\text{eli}}\frac{\lambda }{N}(\tau \left(1-(1+\alpha )w_{0}\right)r_{i}r_{j}\;\;+\delta_{ij}\left(\left(1-2w_{0}\right)r_{i}-\tau (1+\alpha )w_{0}r_{i}∥r{∥}_{1}\right))$$

or in vector notation,

$$J_{w_{0}}=(R^{*}-\frac{1}{N}w_{0}∥r{∥}_{1})r^{\text{dop}}\tau_{\text{dop}}\tau_{\text{eli}}\frac{\lambda }{N}(\tau \left(1-(1+\alpha )w_{0}\right)rr^{T}\;\;+\left(1-2w_{0}-\tau (1+\alpha )w_{0}∥r{∥}_{1}\right)\text{diag}(r)).$$

Like we did in Section C.1, we can compute the determinant of the $k^{\text{th}}$ upper left submatrix $J_{w_{0}}$, which we will denote $J_{w_{0}}^{k}$:

(C15)
$$\det\left(J_{w_{0}}^{k}\right)=(R^{*}-{\frac{1}{N}w_{0}∥r{∥}_{1})}^{k}{\left(r^{\text{dop}}\tau_{\text{dop}}\tau_{\text{eli}}\frac{\lambda }{N}\right)}^{k}{\left(1-2w_{0}-\tau (1+\alpha )w_{0}∥r{∥}_{1}\right)}^{k}\;\;\times \left(\prod_{i=1}^{k}r_{i}\right)\left(1+\frac{\tau \left(1-(1+\alpha )w_{0}\right)}{1-2w_{0}-\tau (1+\alpha )w_{0}∥r{∥}_{1}}\sum_{i=1}^{k}r_{i}\right).$$

(Note that the $∥r{∥}_{1}$ terms that come from the definition of $J_{w_{0}}$ are sums over all N elements of r, while the sum that we get from computing $r^{T}\text{diag}{\left(r\right)}^{-1}r$ in the determinant only includes the first k elements.)

To check the signs of these determinants, first observe that $1-(1+\alpha )w_{0}$ is negative, as can be seen by using the definition of $w_{0}$, equation (B12):

$$1-(1+\alpha )w_{0}=1-\frac{\tau (1+\alpha )∥r{∥}_{1}+1+\alpha }{\tau (1+\alpha )∥r{∥}_{1}+1}\;\;=-\frac{\alpha }{\tau (1+\alpha )∥r{∥}_{1}+1}\;\;<0.$$

Next, observe that $1-2w_{0}-\tau (1+\alpha )w_{0}∥r{∥}_{1}$ is also negative:

$$1-2w_{0}-\tau (1+\alpha )w_{0}∥r{∥}_{1}=1-w_{0}-\left(\tau (1+\alpha )∥r{∥}_{1}+1\right)w_{0}\;\;=1-w_{0}-\left(\tau ∥r{∥}_{1}+1\right)\;\;=-w_{0}-\tau ∥r{∥}_{1}\;\;<0.$$

Thus, the last term in equation (C15) is positive. In addition, this implies that $J_{w_{0}}^{k}$ has a factor of ${\left(-1\right)}^{k}$. If $R^{*}<\frac{1}{N}w_{0}∥r{∥}_{1}$, then we get a second factor of ${\left(-1\right)}^{k}$ canceling the first, so det $\left(J_{w_{0}}^{k}\right)>0$ for all k, and then by Sylvester’s criterion, $J_{w_{0}}$ is positive definite (and thus the fixed point is unstable). On the other hand, if $R^{*}>\frac{1}{N}w_{0}∥r{∥}_{1}$, then the sign of det $\left(J_{w_{0}}^{k}\right)$ is ${\left(-1\right)}^{k}$. But this means that det $\left(-J_{w_{0}}^{k}\right)>0$, so by Sylvester’s criterion, $-J_{w_{0}}$ is positive definite, or equivalently, $J_{w_{0}}$ is negative definite (and thus the fixed point is stable). □

## Appendix D Averaged Model, Random Dopamine Setting

The analysis described in Section A can be easily extended to the random dopamine setting, the only difference being the treatment of the dopamine signal. For the additive and multiplicative models, the mean dopamine signal can be factored out of the drift equation; since in the random dopamine setting $D∼N\left(0,\sigma_{\text{dop}}^{2}\right)$, which has zero mean, it follows that the additive and multiplicative models have zero mean weight drift.

For the corticostriatal model, it is clear from the symmetry of the normal distribution that $D_{+}=\frac{1}{2}E[|D|]$ and $D_{-}=-D_{+}$, where $E[|D|]=\sigma_{\text{dop}}\sqrt{2/\pi }$. Plugging these into equation (A8), we get:

$$\dot{w}=\frac{1}{2}E[|D|]r^{\text{dop}}\tau_{\text{dop}}\tau_{\text{eli}}\frac{\lambda }{N}(\tau \langle w,r\rangle (1-(1+\alpha )w)⊙r+(1-w)⊙w⊙r\;\;+\tau \langle w,r\rangle (1-(1+\alpha )w)⊙r-\alpha w⊙w⊙r)\;\;=\frac{1}{2}E[|D|]r^{\text{dop}}\tau_{\text{dop}}\tau_{\text{eli}}\frac{\lambda }{N}(2\tau \langle w,r\rangle r+w⊙r)⊙(1-(1+\alpha )w).$$

This equation has a single fixed point at $w_{i}=1/(\alpha +1)$ for all i. The Jacobian at this point is a diagonal matrix with negative diagonal elements:

$$J=-\frac{1}{2}(1+\alpha )E[|D|]r^{\text{dop}}\tau_{\text{dop}}\tau_{\text{eli}}\frac{\lambda }{N}\text{diag}(2\tau \langle w,r\rangle r+w⊙r).$$

Consequently, this fixed point is stable.

## Appendix E Single Eligibility Trace

We now revisit the question of whether to use a single eligibility trace summing up both positive (corresponding to pre-before-post spike pairs) and negative (post-before-pre) contributions, as is done in Clapp et al. (2024), or to use two different traces for the positive and negative components, as we do elsewhere in the paper. We had several reasons for focusing on models with two different eligibility traces. One was analytical convenience: the use of two traces is necessitated by the assumption, made by Gütig et al. (2003); Rubin et al. (2001) as well as in this paper, that the contributions to the weight changes made by individual spike pairs sum independently, a natural assumption that greatly simplifies analysis. With only one eligibility trace, different spike pairs may cancel each other out, rendering this independence assumption invalid. We therefore cannot derive averaged forms of the single-trace models like we did for the two-trace models. A second justification for the focus on two-trace models is that there is experimental evidence suggesting that the brain in fact uses two different traces, one for LTP and one for LTD He et al. (2015). These findings describe cortical pyramidal cells, rather than corticostriatal synapses, but similar mechanisms may be at play here too.

We test a single-trace version of our model that replaces equation (3) with

(E16)
$$\frac{dE_{i}}{dt}=\rho^{\text{post}}(t)A_{i}^{\text{pre}}(t)-\gamma \rho_{i}^{\text{pre}}(t)A^{\text{post}}(t)-\frac{1}{\tau_{\text{eli}}}E_{i}(t)$$

where γ≥1 is a scaling parameter controlling the strength of negative eligibility terms relative to positive terms. The single-trace differential equation for the weights in the additive and multiplicative cases is given by

(E17)
$$\frac{dw_{i}}{dt}=\{\begin{array}{ll} \lambda D(t)f_{+}\left(w_{i}(t)\right)E_{i}(t) & \text{if}\;E_{i}(t)\geq 0\\ \lambda D(t)f_{-}\left(w_{i}(t)\right)E_{i}(t) & \text{if}\;E_{i}(t)<0. \end{array}$$

and for the corticostriatal model is given by

$$\frac{dw_{i}}{dt}=\{\begin{array}{ll} \lambda D(t)\left(1-w_{i}(t)\right)E_{i}(t) & \text{if}\;D(t)E_{i}(t)\geq 0\\ \lambda D(t)\alpha w_{i}(t)E_{i}(t) & \text{if}\;D(t)E_{i}(t)<0. \end{array}$$

The single-trace version of the corticostriatal model is largely equivalent to the model in described in Clapp et al. (2024), although they use different scaling factors and time constants for pre- and postsynaptic activity.

One important characteristic of the single-trace versions of the additive and multiplicative models (equation (E17)) is that they are largely insensitive to variations in α. This insensitivity arises because $E_{i}(t)$ is usually positive, since presynaptic spikes directly cause postsynaptic spikes after a delay of ϵ and not vice versa, which tilts the balance to favor positive eligibility. Hence, the α-dependent $f_{-}$ term is only rarely used. The α parameter is therefore not an effective way of adjusting the relative strengths of the positive and negative components of the eligibility trace. This observation motivates the introduction of the parameter γ in equation (E16) to provide a better means of controlling the relative strengths of the two components in the single-trace models. (The single-trace corticostriatal model is still sensitive to α because it depends on the sign of the product $D(t)E_{i}(t)$, rather than just $E_{i}(t)$, so the term with α will have an impact when $E_{i}(t)>0$ and D(t)<0.)

We show simulations of the single-trace models in the random dopamine, reward prediction, and action selection settings in Figure 15, Figure 16, and Figure 17, as well as for action selection with contingency switching in Figure 18. In the random dopamine setting (Figure 15) we vary γ in addition to α; in the reward prediction setting (Figure 16) we vary γ and keep α=1 fixed. The single-trace and two-trace versions of the additive model behave identically when α=γ=1, because in this case positive and negative eligibility are treated the same. In the random dopamine setting all three models behave qualitatively similarly to the two-trace versions (Figure 3), and they appear largely insensitive to γ. In the action selection setting results again qualitatively match those found with the two-trace models (Figures 8 and 10). In the reward prediction setting, on the other hand, some differences between single-trace and two-trace model dynamics are visible (cf. Figure 4), especially for larger values ofγ. While the solution planes become increasingly unstable as γ increases, similar to the effect seen in the two-trace models as α increases, the precise form of the dynamics appears to differ considerably (e.g. in Figure 16h, trajectories seem to spread out rather than converge to a fixed point under the multiplicative model). Overall, using a single eligibility trace does not seem to significantly improve performance on these tasks and makes the dynamics much more difficult to analyze.
