# Supplementary material for: Distinct dopaminergic spike-timing-dependent plasticity rules are suited to different functional roles
Source: bioRxiv. 2024 Jun 27:2024.06.24.600372. Preprint. [Version 1] doi: 10.1101/2024.06.24.600372 (PMC11230239; doi:10.1101/2024.06.24.600372)
Supplement: Supplementary file 1 [file NIHPP2024.06.24.600372v1-supplement-1.pdf]

bioRxiv preprint doi: https://doi.org/10.1101/2024.06.24.600372; this version posted June 27, 2024. The copyright holder for this preprint (which was not certified by peer review) is the author/funder, who has granted bioRxiv a license to display the preprint in perpetuity. It is made available under aCC-BY 4.0 International license.

Supporting information

S1 Fig. Performance in the action selection setting as delay is varied with and without sustained activity. Plots show weights after 1000 steps for additive (A), multiplicative (B), and corticostriatal (C) models. With no sustained activity, both input channels are silenced during the delay period, while with sustained activity, the

bioRxiv preprint doi: https://doi.org/10.1101/2024.06.24.600372; this version posted June 27, 2024. The copyright holder for this preprint (which was not certified by peer review) is the author/funder, who has granted bioRxiv a license to display the preprint in perpetuity. It is made available under aCC-BY 4.0 International license.

input to the selected channel is maintained at a level of 70% (see Fig 2).

S2 Fig. Model performance in the action selection setting with slow task switching. Plots show weights (A-F) and probability of taking the correct action (G-I) versus time for the additive (A, D, G), multiplicative (B, E, H), and corticostriatal (C, F, I) models. The input switches every 1000 steps between $r = (15, 5)$ s^{-1}, $R_1^* = 2$, $R_2^* = 1$ and $r = (5, 15)$ s^{-1}, $R_1^* = 1$, $R_2^* = 2$. Channel 1 is displayed in (A-C) and channel 2 in (D-F); each one consists of two weights. The optimal weight vectors are $w^1 = (1, 0)$ and $w^2 = (0, 1)$, which would, by design, allow the model to preferentially choose action 1 in state 1 and action 2 in state 2. In these plots $\lambda = 0.005$.

S3 Fig. Model performance in the action selection setting with fast task switching. Plots show weights (A-F) and probability of taking the correct action (G-I) versus time for the additive (A, D, G), multiplicative (B, E, H), and corticostriatal (C, F, I) models. Parameters are the same as in S2 Fig except that task switching occurs at every simulation step.

S4 Fig. Weight evolution over time in the random dopamine setting for single-trace models. Columns show the additive (A, D, G), multiplicative (B, E, H), and corticostriatal (C, F, I) models. (A-C) the initial weight w_{init} is varied while $\alpha = 1$ and $\gamma = 1$ are fixed. (D-F) α is varied while $w_{\text{init}} = 0.5$ and $\gamma = 1$ are fixed. (G-I) γ is varied while $w_{\text{init}} = 0.5$ and $\alpha = 1$ are fixed.

S5 Fig. Model performance in the action selection setting for single-trace models. Plots show weights (A-C) and probability of taking the correct action (D-F) versus time for the additive (A, D), multiplicative (B, E), and corticostriatal (C, F) models. In these simulations $\gamma = 1$.

S6 Fig. Model performance in the action selection setting with contingency switching for single-trace models. Plots show weights (A-C) and probability of taking the correct action (D-F) versus time for the additive (A, D), multiplicative (B, E), and corticostriatal (C, F) models. Here $\gamma = 1$, and the other parameters are the same as those used in Fig 8.

S1 Appendix. Proofs and analytical results describing the weight dynamics in random dopamine and reward prediction settings.

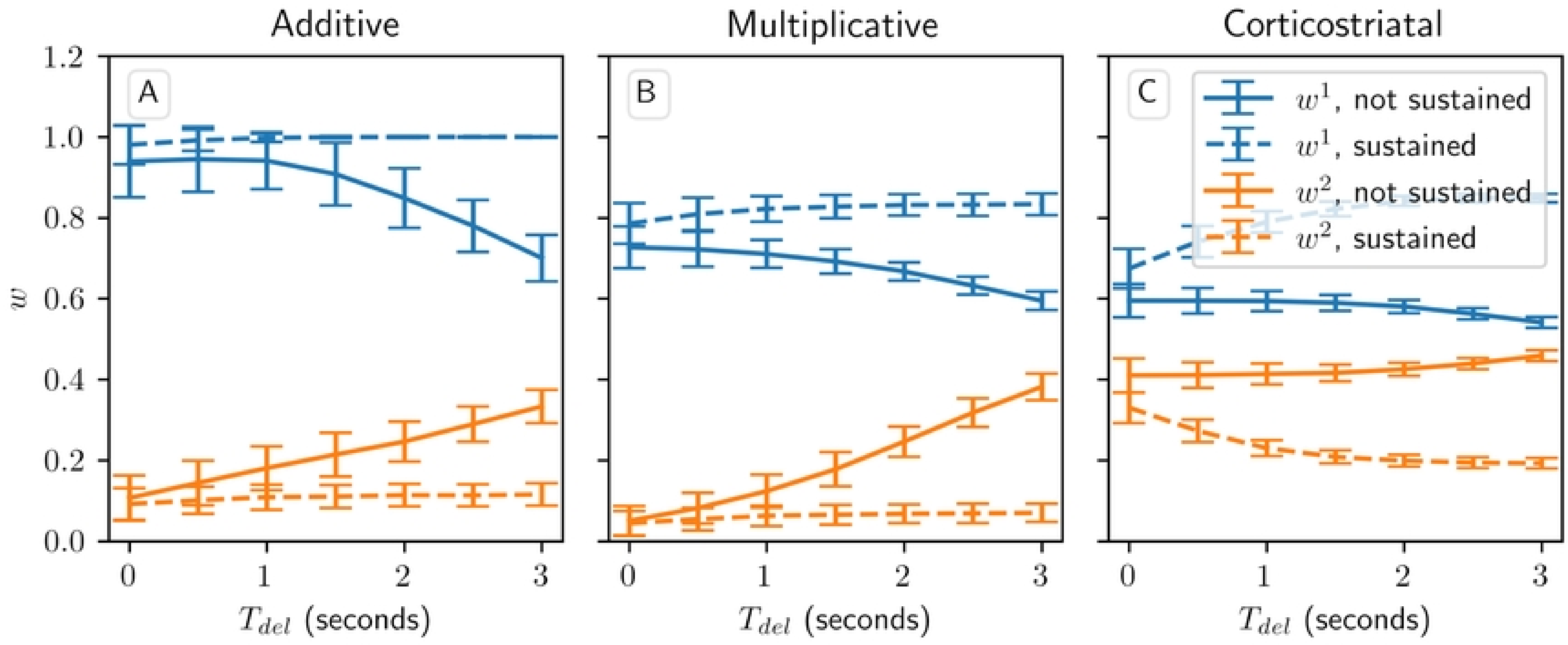
Additive
Multiplicative
Corticostriatal
A
B
C
w^1, not sustained
w^1, sustained
w^2, not sustained
w^2, sustained
w
1.2
1.0
0.8
0.6
0.4
0.2
0.0
0
1
2
3
T_{del} (seconds)

S1_fig

bioRxiv preprint doi: https://doi.org/10.1101/2024.06.24.600372; this version posted June 27, 2024. The copyright holder for this preprint (which was not certified by peer review) is the author/funder, who has granted bioRxiv a license to display the preprint in perpetuity. It is made available under aCC-BY 4.0 International license.

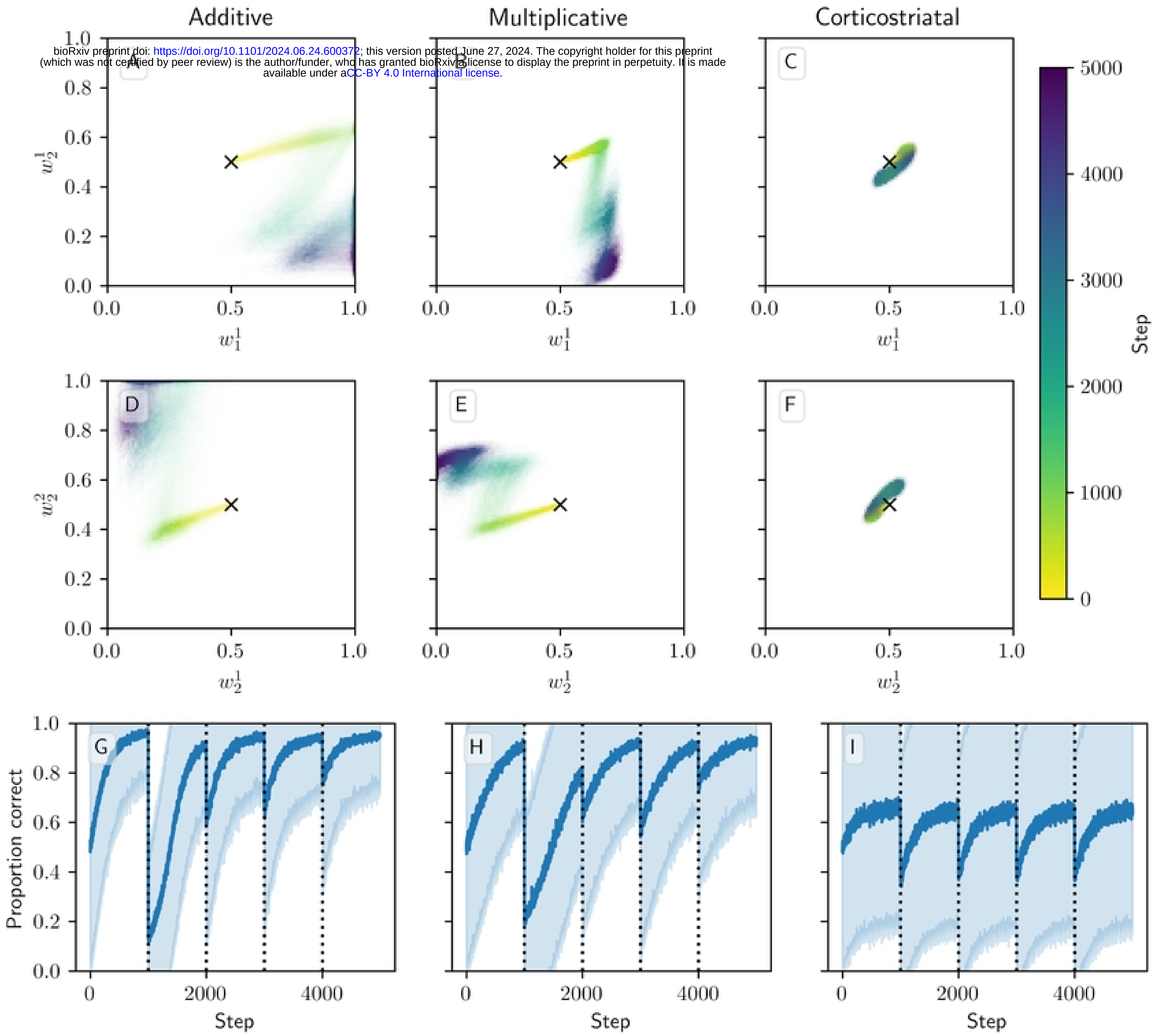

S2_fig

bioRxiv preprint doi: https://doi.org/10.1101/2024.06.24.600372; this version posted June 27, 2024. The copyright holder for this preprint (which was not certified by peer review) is the author/funder, who has granted bioRxiv a license to display the preprint in perpetuity. It is made available under aCC-BY 4.0 International license.

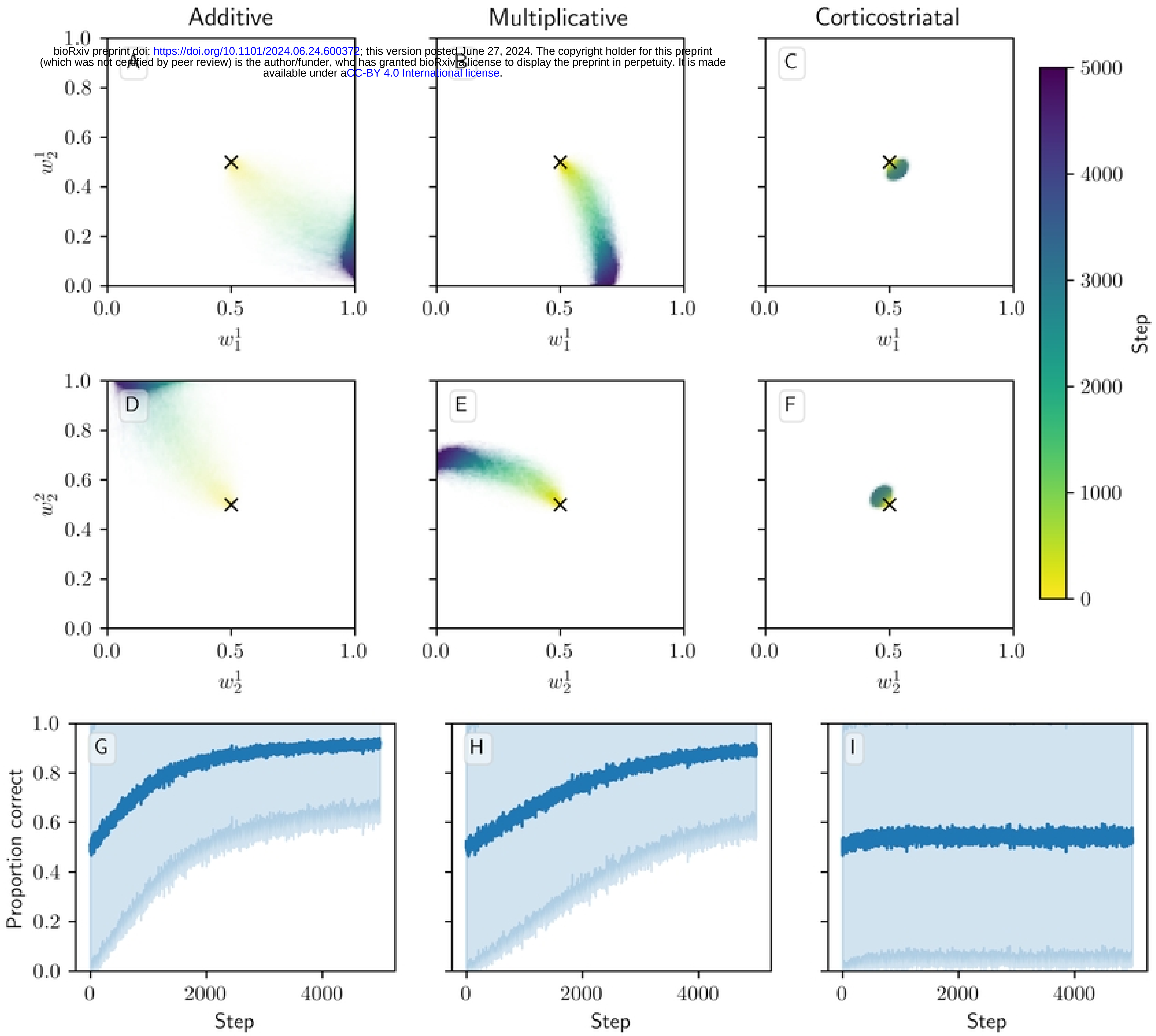

S3_fig

bioRxiv preprint doi: https://doi.org/10.1101/2024.06.24.600372; this version posted June 27, 2024. The copyright holder for this preprint (which was not certified by peer review) is the author/funder, who has granted bioRxiv a license to display the preprint in perpetuity. It is made available under aCC-BY 4.0 International license.

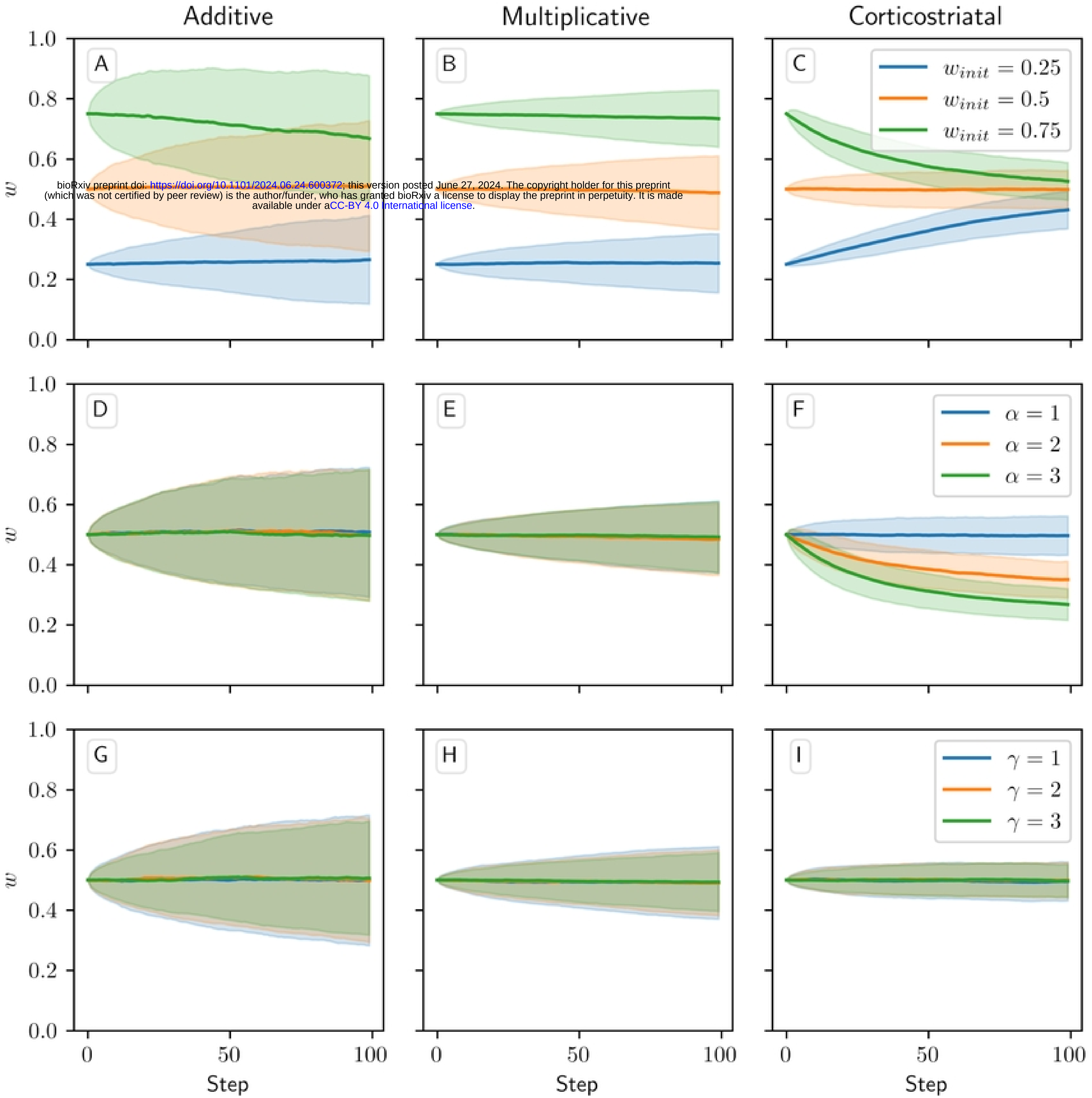

S4_fig

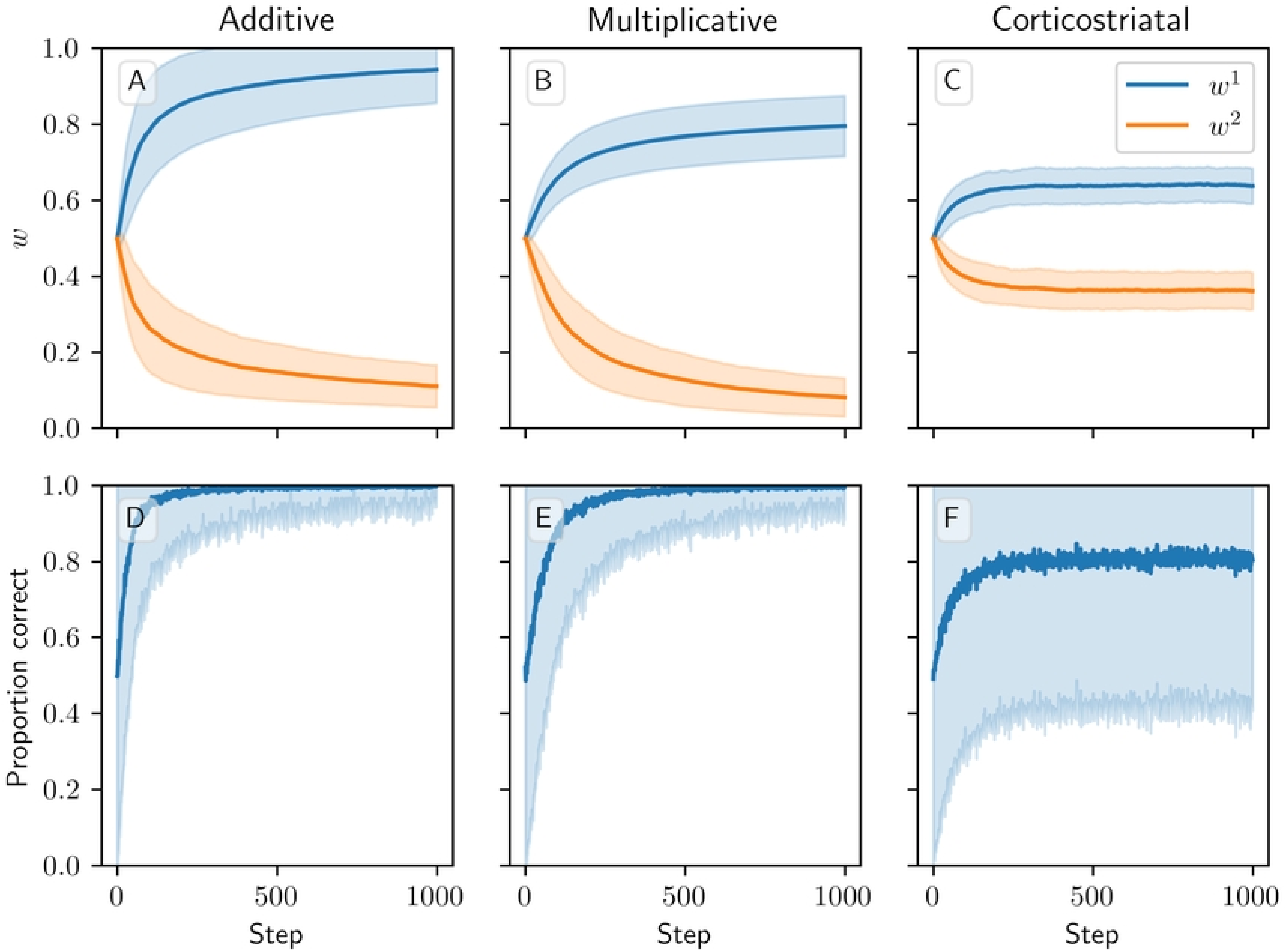

Additive
Multiplicative
Corticostriatal
A
B
C
D
E
F
w^1
w^2
w
Proportion correct
Step
0
500
1000
0.0
0.2
0.4
0.6
0.8
1.0

S5_fig

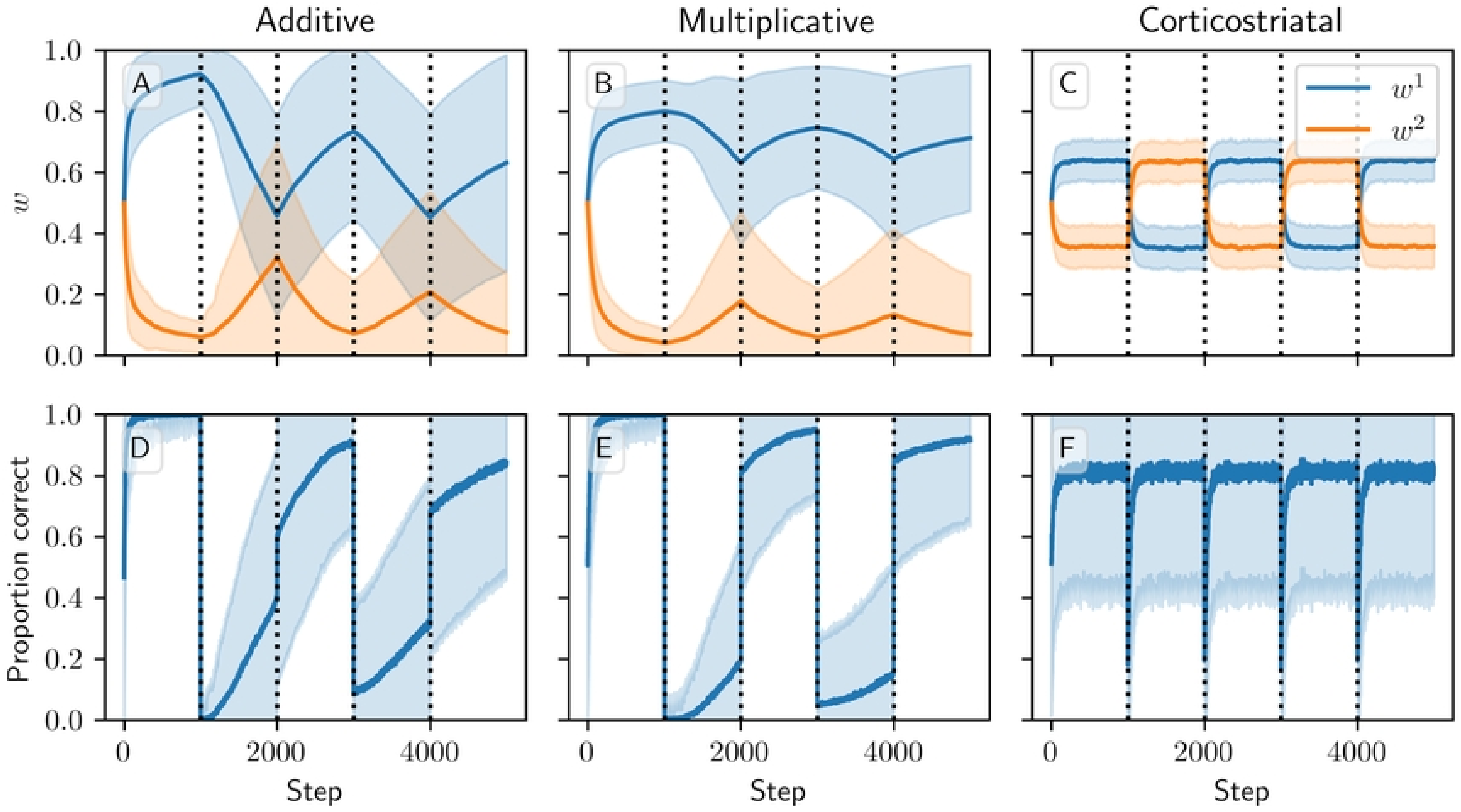

Additive
Multiplicative
Corticostriatal
A
B
C
D
E
F
w^1
w^2
w
Proportion correct
1.0
0.8
0.6
0.4
0.2
0.0
0
2000
4000
Step

S6_fig
